# Bond orders for intermolecular interactions in crystals: charge transfer, ionicity and the effect on intramolecular bonds

**DOI:** 10.1107/S2052252518010758

**Published:** 2018-08-29

**Authors:** Khidhir Alhameedi, Amir Karton, Dylan Jayatilaka, Sajesh P. Thomas

**Affiliations:** a School of Molecular Sciences, University of Western Australia, 35 Stirling Highway, Perth 6009, Australia; b College of Education for Pure Science, University of Karbala, Karbala, Iraq; c Centre for Materials Crystallography, Department of Chemistry and iNano, Aarhus University, Langelandsgade 140, Aarhus 8000, Denmark

**Keywords:** intermolecular interactions, bond order, ionicity, hydrogen bonding, halogen bonding, crystal engineering, computational modelling, molecular crystals

## Abstract

The question of whether intermolecular interactions in crystals originate from localized atom⋯atom interactions or as a result of holistic molecule⋯molecule close packing is a matter of continuing debate. In this context, the newly introduced Roby–Gould bond indices are reported for intermolecular ‘σ-hole’ interactions, such as halogen bonding and chalcogen bonding, and compared with those for hydrogen bonds. A series of 97 crystal systems exhibiting these interaction motifs obtained from the Cambridge Structural Database (CSD) has been analysed. In contrast with conventional bond-order estimations, the new method separately estimates the ionic and covalent bond indices for atom⋯atom and molecule⋯molecule bond orders, which shed light on the nature of these interactions. A consistent trend in charge transfer from halogen/chalcogen bond-acceptor to bond-donor groups has been found in these intermolecular interaction regions *via* Hirshfeld atomic partitioning of the electron populations. These results, along with the ‘conservation of bond orders’ tested in the interaction regions, establish the significant role of localized atom⋯atom interactions in the formation of these intermolecular binding motifs.

## Abstract 2

Roby–Gould bond orders for intermolecular interactions such as hydrogen bonds, halogen bonds and chalcogen bonds in molecular crystals have been explored. Bond-order values place these interactions on a scale representing their relative strengths, in conjunction with a chemist’s notion of bonds.

## 1. Introduction   

The identification and characterization of novel intermolecular interactions and the probing of their contribution to crystal packing are topics of interest in crystal engineering (Desiraju, 2013 ▸). In recent years, non-bonding interactions such as halogen bonds (XBs) (Cavallo *et al.*, 2016 ▸; Politzer *et al.*, 2013 ▸; Desiraju *et al.*, 2013 ▸; Bui *et al.*, 2009 ▸), chalcogen bonds (YBs) (Brezgunova *et al.*, 2013 ▸; Wang *et al.*, 2009 ▸; Thomas *et al.*, 2015 ▸), carbon bonds (Mani & Arunan, 2013 ▸; Thomas *et al.*, 2014 ▸; Escudero-Adán *et al.*, 2015 ▸) and pnicogen bonds (Bauzá *et al.*, 2013 ▸; Scheiner, 2013 ▸; Sarkar *et al.*, 2015 ▸) have attracted significant attention from both experimental and computational chemists. These interactions, broadly known as σ-hole interactions, have been identified as originating from the electropositive regions (σ-holes) around atoms which are directed to nucleophilic atoms such as O, N, F *etc.* (Clark *et al.*, 2007 ▸). While the occurrence of prominent σ-hole interactions such as XBs and YBs is increasingly being reported in the solid and solution states, it is clear that a more quantitative understanding of these interactions is needed to assess their significance in supramolecular chemistry and crystal engineering.

Spackman and co-workers have pointed out that, irrespective of their classifications, all these σ-hole interactions have a common origin of electrostatic complementarity (Edwards *et al.*, 2017 ▸). Furthermore, in a recent essay Dunitz questioned the validity of such atom⋯atom ‘bonds’, arguing that they were seldom structure-determining and needed to be regarded as a result of holistic molecule⋯molecule interactions in crystal packing (Dunitz, 2015 ▸). Alternative arguments were raised by Desiraju, who noted that short atom⋯atom contact distances observed in crystals could indeed be linked to kinetically derived structural units and that they were most often found to be ‘bonding’ (Thakur *et al.*, 2015 ▸). Lecomte *et al.* (2015 ▸) argued that such contacts could be characterized by electron-density bond paths indicating stabilizing interactions and they could have the potential to determine crystal structures.

In the light of this atom⋯atom *versus* molecule⋯molecule interaction debate, and in the general interest of exploring the nature and relative strengths of such intermolecular inter­actions, we asked these simple and rather fundamental questions: ‘How much of a bond is an intermolecular interaction?’, and ‘How ionic or covalent is a σ-hole interaction?’. To answer these, we estimated the bond orders of a series of 97 molecular complexes selected from the Cambridge Structural Database (CSD; Groom *et al.*, 2016 ▸) which exhibit halogen bonds, chalcogen bonds and the well known classes of hydrogen bonds, using the Roby–Gould bond indices recently introduced by us (Gould *et al.*, 2008 ▸). While the commonly employed approaches to quantifying such interactions are based on interaction energy (Mackenzie *et al.*, 2017 ▸) (a molecule⋯molecule descriptor) and electron-density topology (Johnson *et al.*, 2010 ▸; Grabowski, 2011 ▸; Zou *et al.*, 2017 ▸) (essentially an atom⋯atom descriptor), the Roby–Gould approach covers both these aspects in terms of separate atom⋯atom and molecule⋯molecule bond indices. As opposed to simplistic chemical descriptors such as bond-valence models (Brown, 2009 ▸), the Roby–Gould method uniquely and separately defines the ionic and covalent bond indices, which add up in a Pythagorian fashion to provide the total bond order (Gould *et al.*, 2008 ▸).

Our previous work has demonstrated that the Roby–Gould bond indices (RGBIs) correlate well with a chemist’s notion of bonding, in line with the Lewis picture and Pauling’s percentage ionicity estimates (Gould *et al.*, 2008 ▸). Hence, the RGBIs evaluated in this study provide a means of comparing intermolecular interactions with well known classes of bonds on a relative scale. Our recent study showed that RGBIs could be used to predict the fragmentation of molecules and thereby the base peaks in mass spectra (Alhameedi *et al.*, 2018 ▸).

Although we have recently applied the Roby–Gould method to quantum crystallographic X-ray wavefunctions to analyse chemical bonding (Grabowsky *et al.*, 2012 ▸; Thomas *et al.*, 2015 ▸), the bond orders of intermolecular interactions remain largely underexplored. The few computational studies in the literature probing the bond orders of intermolecular interactions are based on the natural bond orbital (NBO) approach (Shahi & Arunan, 2014 ▸). However, the NBO approach has major limitations in its applicability to intermolecular interactions, as it is based on the projection of the electron population onto Lewis-like orbitals (Stone, 2017 ▸). In the Roby–Gould method, the electronic population is projected onto occupied atomic natural orbitals and the bond-order estimation does not depend on the criteria of whether the orbitals are Lewis-like or not. This makes the Roby–Gould method a superior approach in studying intermolecular interactions, as intermolecular interactions do not obey Lewis-type bonding.

Here, we evaluate the extent of electron sharing and charge transfer in σ-hole interactions and hydrogen bonds using RGBIs. We have also examined the correlations between bond order, interaction distance, charge transfer and intermolecular interaction energy. In addition, the ‘conservation of bond order’ in the interaction regions (as a result of the formation of a *D*—*X*⋯*A* interaction and the weakening of the *D*—*X* covalent bond) has been tested in a selected subset of examples.

## 2. Methods and materials   

### 2.1. Roby–Gould bond indices   

The key advantages of the Roby–Gould method, and the reason we have applied this to study intermolecular inter­actions, are the following:

(i) The Roby–Gould method produces two independent covalent and ionic bonds using the expectation values of quantum mechanical operators, and which are furthermore derived from well known ideas of bonding and antibonding orbitals.

(ii) It is well defined for any quantum chemical method and using any kind of basis set. In particular, the results converge when using large basis sets.

(iii) It produces bond indices not only between atoms but also between groups of atoms.

(iv) It produces reasonable results (according to the Lewis theory) for bond indices at transition states *i.e.* ‘half’ bonds (Gould *et al.*, 2008 ▸).

(v) The RGBIs agree with the indices from classical Lewis structures which are widely used in organic chemistry, and for classic ionic bonds such as Li—F and Na—F we obtain ionic bond indices of nearly 1, with a percentage ionicity of around 90%.

Despite the fact that the RGBIs correlate very well with the standard Lewis picture of chemical bonding for intramolecular bonds, one should be reminded of the non-uniqueness of bond-order estimation approaches such as the Roby–Gould method, as they depend upon the partitioning of either real space or Hilbert space (in the case of RGBIs), which is non-unique. Hence, we recommend that the RGBI values be used to understand trends and relative strengths rather than as absolute indications of bonding; for the latter, bond-dissociation energies may be more appropriate.

Apart from RGBIs, a number of different bond-order definitions have been reported to characterize the nature of a chemical bond: the quantum theory of atoms in molecules delocalization index (QTAIM DI) (Bader & Stephens, 1974 ▸, 1975 ▸), the shared-electron distribution index (SEDI) (Ponec & Cooper, 2005 ▸), Hirshfeld-I SEDI [applying the iterative Hirshfeld-I approach (Bultinck *et al.*, 2007 ▸) to define the atomic domains], Mayer bond order (MBO) (Mayer, 1983 ▸), bond orders derived from natural bond orbital (NBO) analysis and NBO bond order are the most popular examples. These bond orders are generally considered to be descriptors of electron sharing, so these approaches are sometimes referred to as covalent bond orders, whereas RGBIs have a separate definition for the ionic operator, in addition to the covalent operator, which can be applied for polar and nonpolar chemical bonds.

The Roby covalent bond index is defined based on the shared electron population *s*_*AB*_ between any two atoms *A* and *B* as 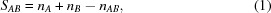
where the electron populations for the subspaces of atoms *A*, *B* and the diatom *AB* are given by *n*_*A*_, *n*_*B*_ and *n*_*AB*_, respectively: 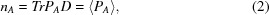


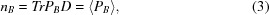


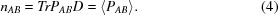
Here, 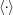 is the usual expectation value with respect to a molecular wavefunction, *D* is the corresponding one-electron reduced density operator, *Tr* denotes the trace, and *P_A_* and *P_B_* are projection operators (see Gould *et al.*, 2008 ▸). In this study, we have used the extended Roby analysis and the new definitions for covalent, ionic and total bond indices, respectively (Gould *et al.*, 2008 ▸), 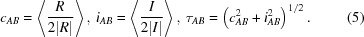

*R* and *I* are the Roby covalent operator and the corresponding ionic operator, respectively, 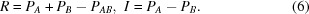
Thus, in this approach, a chemical bond is regarded as a two-dimensional quantity characterized by a pair of numbers (*c*, *i*) obtained quantum mechanically as the expectation value of two operators, and whose magnitude is τ. The paper by Gould *et al.* justifies the form of these operators, which turn out to be constructed as a sum of terms over certain ‘angle’ subspaces, *e.g.*

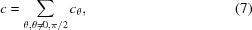
and likewise 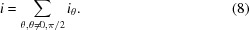
The angles θ characterize either the degree of overlap, or alternatively the angle between the orbitals on the two atom centres which have ‘maximum or minimum overlap’ (for the covalent bond index) or ‘maximum or minimum electron transfer’ (for the ionic bond index). Within each of the angle subspaces, the bond index is calculated as half the number of electrons in the bonding orbitals minus half the number of electrons in the antibonding orbitals, 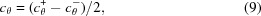


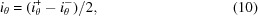

*i.e.* Coulson’s rule, but in the Roby–Gould theory this is generalized to any pair of atoms in a molecule. Having defined the covalent and ionic indices, the percentage covalency and ionicity of a chemical bond may be defined as 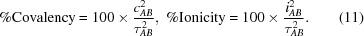
For more details, see Gould *et al.* (2008 ▸).

### 2.2. Selection of the data set   

**Figure 1:**
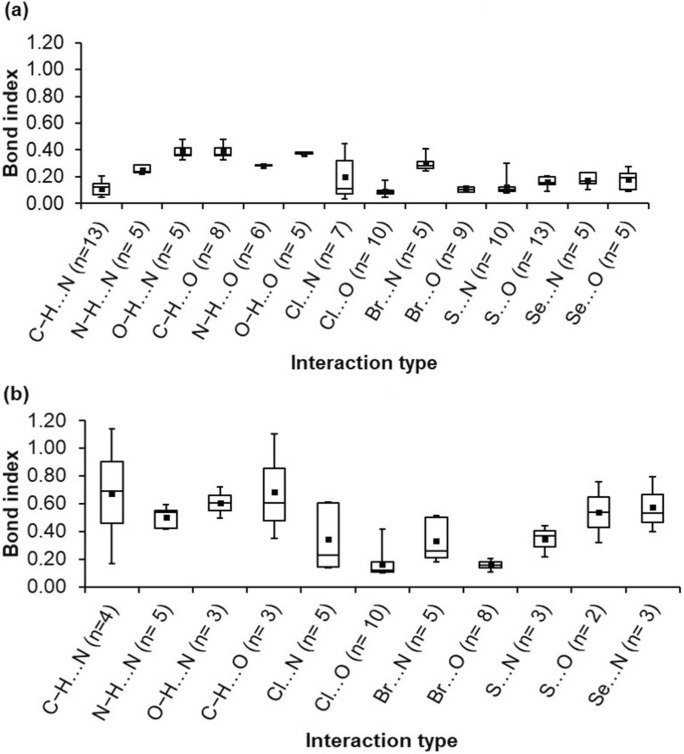
Distributions of RGBI values for (*a*) atom⋯atom bond indices and (*b*) molecule⋯molecule bond indices for different interaction types, represented in box-and-whisker plots. The whiskers represent the range of RGBI values, the height of the boxes represents the interquartile range, and the dots inside the boxes represent the median for each interaction type. For molecule⋯molecule bond indices, only dimers with single atom⋯atom short contacts are included in plot (*b*). The number of interactions (*n*) in each class is given in parentheses.

We have restricted our attention to short intermolecular atom⋯atom distances *D*—*X*⋯*A* between a donor-atom pair *D*—*X* (bond donor) and an acceptor atom *A*. Specifically, we considered hydrogen bonds with *D*—*X* = C—H, N—H, O—H with acceptor atoms *A* = O, N, and we also considered weak halogen and chalcogen atom⋯atom distances *D*⋯*A* between all donor atoms *D* = Cl, Br, S, Se. For this purpose, we searched the CSD using geometric and structural constraints as follows: (i) no disorder, only organic molecules, only single-crystal data; (ii) the number of atoms in the asymmetric unit was less than 20; (iii) interaction distances were less than the sum of the van der Waals radii by at least 0.2 Å; and (iv) for examples of hydrogen-bonded complexes only neutron diffraction structures were chosen, and for XBs and YBs we have reset the *X*—H (*X* here is a C, N or O atom) bond lengths to the normalized neutron diffraction distances. The number of interactions of each type is also given beside each class on the abscissa of Fig. 1 ▸. The molecular structures of all selected dimers with their interaction distances are given in Section S8 of the supporting information.

### 2.3. Wavefunctions and interaction energies   

Cartesian geometries for selected monomers and dimers involved in the short atom⋯atom contacts under study were obtained from the crystallographic information files (CIFs) in the CSD. Wavefunctions were calculated at the single-point crystal geometry M062x/Def2TZVP level with Cartesian Gaussian basis sets, using the *GAUSSIAN09* program (Frisch *et al.*, 2009 ▸). The interaction-energy calculations for the molecular dimers were performed without BSSE correction, considering the large basis set used. Further, for testing the idea of ‘conservation of bond orders’ a series of 15 dimers were selected based on their small molecular size, and they were optimized at the M062x/Def2SVP level for the bond-order analysis.

### 2.4. Bond indices and Hirshfeld charges   

RGBIs were calculated using the method explained by Gould *et al.* (2008 ▸) using the freely available *TONTO* program package (Jayatilaka & Grimwood, 2003 ▸) with the *GAUSSIAN* FChk files (the text version of the *GAUSSIAN* checkpoint file). Hirshfeld charges (Hirshfeld, 1977 ▸) were calculated from the *GAUSSIAN* FChk files using *TONTO*.

## 3. Results and discussion   

### 3.1. Bond indices for different classes of non-covalent interactions   

We have analysed the RGBIs for the data set of 106 interacting molecular dimers, with the atom⋯atom and mol­ecule⋯mol­ecule RGBI values calculated separately. The results for halogen- and chalcogen-bonding interactions are presented in Tables 1 ▸ and 2 ▸. The RGBI values for a set of 42 molecular pairs formed by hydrogen bonds are given in Table S1 of the supporting information for comparison, and only summary statistics are presented here.

#### 3.1.1. Atom⋯atom bond indices   

Atom⋯atom RGBI values were calculated by considering the projection into the atomic natural orbitals of the two atoms involved in the intermolecular interaction (atoms *X* and *A* in an interaction *X*⋯*A*). The distribution of total atom⋯atom RGBI values (τ) is shown in Fig. 1 ▸(*a*). For the hydrogen-bond (HB) interactions (Table S1 in the supporting information), we generally observe that a hierarchy of RGBI values can be shown as
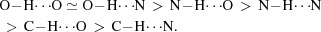
RGBI values for each type of interaction were averaged to find this hierarchy. The order is in line with the chemical wisdom derived from crystal structural analyses over the years and from crystal-engineering experiments. This result shows that a *D*—H bond donor group with a higher electronegativity atom *D* leads to stronger HBs. The RGBI values of strong HBs such as O—H⋯O, N—H⋯O and O—H⋯N are in the range 0.22–0.48, and those for weak HBs like C—H⋯O and C—H⋯N are in the range 0.05–0.21. For σ-hole interactions, the RGBI value ranges are 0.04–0.45 for halogen bonds (XBs) and 0.08–0.3 for chalcogen-bond (YB) interactions (Tables 1 ▸ and 2 ▸, respectively). For XBs, a hierarchy of RGBI values can be shown as
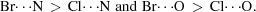
This may be rationalized based on the higher polarizability of a Br atom compared with a Cl atom when acting as halogen-bond donors. Similarly, the following trend is observed for YBs: 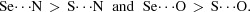
The hierarchies of the various interaction types discussed above are based on average values of the total RGBIs. It may be noted that the RGBI values of some interaction types show a wide range of values, as represented by the whiskers in Fig. 1 ▸. In general, a hierarchy of HB > XB > YB is observed in terms of atom⋯atom bond orders.

#### 3.1.2. Molecule⋯molecule bond indices   

Mol­ecule⋯mol­ecule RGBI values were calculated by considering the projection into two groups of the atomic natural orbitals belonging to all atoms in the interacting molecules. The distribution of total molecule⋯molecule RGBI values (τ) is presented in Fig. 1 ▸(*b*) (only dimers with a single atom⋯atom short contact are included in the plot). The RGBI values obtained for interactions between groups of atoms are typically higher than those for localized atom⋯atom values (with a few exceptions; see Section 3.3). This might be expected, as there is more sharing and transfer of electrons from one group to another, as shown in Tables 1 ▸ and 2 ▸. We observe that strong HBs such as N—H⋯O, O—H⋯N and O—H⋯O are characterized by the highest molecule⋯molecule bond-index values, ranging from 0.42 to 1.42. Weak HBs such as C—H ⋯N and C—H⋯O show RGBI values ranging from 0.17 to 1.14. For XBs, the molecule⋯molecule bond-index values range from 0.10 to 0.73. For YBs, the corresponding RGBI range is 0.22–1.52. When the bond orders are averaged for each class of interaction, a hierarchy of HB > XB > YB is observed in terms of molecule⋯molecule bond indices, as was the case with the atom⋯atom bond indices.

### 3.2. Visualization of Roby–Gould hybrid orbitals   

As explained above, the Roby–Gould indices are constructed as the sum of terms from individual covalent bonding and antibonding orbitals, and ionic bonding and antibonding orbitals. It is useful to examine ionic and covalent orbitals separately, to see if they have any relevance to the orbitals that are used in the theory of homonuclear diatomics. It is straightforward to conceive a covalent bonding orbital (an orbital with a maximum shared population in the interaction region between atoms *A*⋯*B*) and a covalent antibonding orbital (an orbital with a minimum shared population between atoms *A* and *B* and a maximum population away from the *A*⋯*B* interaction region).

**Figure 2:**
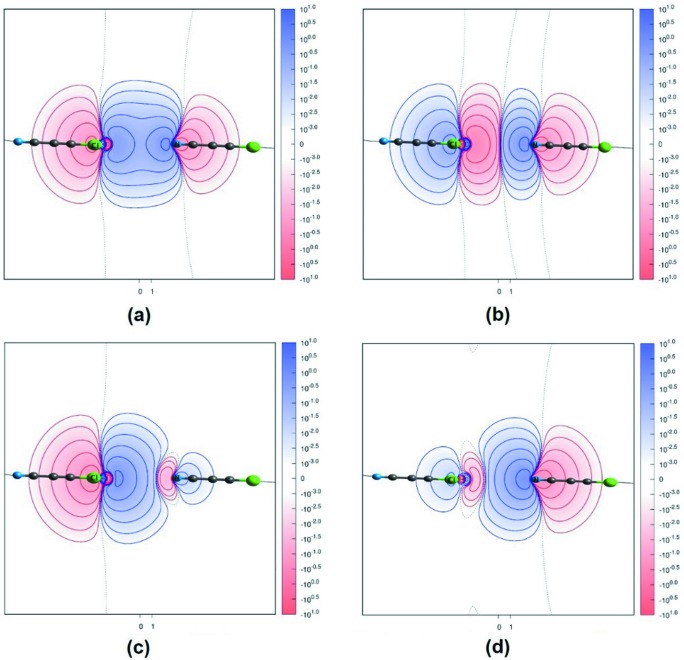
Roby–Gould orbitals used in the estimation of Cl⋯N atom⋯atom bond orders. (*a*) Covalent bonding, (*b*) covalent antibonding, (*c*) ionic bonding and (*d*) ionic antibonding for the Cl⋯N interaction in the CCACEN dimer, θ = 83°.

The ionicity in an intermolecular interaction can be attributed to a putative charge transfer from atom *A*→*B*, resulting in a favourable interaction between partially charged atoms *A* ^(δ+)^⋯*B* ^(δ−)^. Hence, the ionic bonding orbitals correspond to those orbitals representing a lower charge density around *A* and an accumulated charge density around *B*. Similarly, orbitals with an opposite charge-density distribution (accumulated population around *A* and depletion around *B*) represent ionic antibonding orbitals. This is demonstrated in a representative example of Cl⋯N halogen bonding in the crystal structure of CSD refcode CCACEN. Table 3 ▸ shows the covalent and ionic Roby–Gould populations in the bonding and antibonding modes for main three paired orbitals that are involved in the Cl⋯N interaction of the CCACEN dimer. We see that the shared population with θ = 83° makes a greater contribution to the covalent bond index than do the other angles, while the transferred population with θ = 89° makes a greater contribution to the ionic bond index. Fig. 2 ▸ presents the Roby–Gould hybrid orbitals for one of these three covalent and ionic pairs with θ = 83° as an example of halogen bonding and antibonding orbitals for the Cl⋯N interaction.

### 3.3. Insights into the nature of interactions: breakdown of bond index into ionic and covalent bond indices   

**Figure 3:**
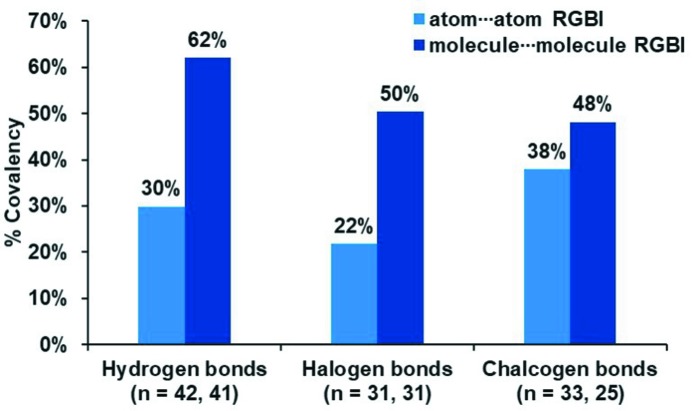
Average covalency percentages for atom⋯atom bond indices (light blue) and molecule⋯molecule bond indices (dark blue) for hydrogen, halogen and chalcogen bonds. The number of interactions and molecular dimers studied for each type are given in parentheses. (See Fig. S2 in the supporting information for bond-order-weighted covalency percentages).

One of the interesting questions about intermolecular interactions is how ionic or covalent they are. We address this in terms of the percentage ionicity and covalency from the RGBI values. Fig. 3 ▸ shows the percentage of the covalent RGBIs evaluated on the data set of 106 interactions (in 97 unique molecular dimers) with all considered interaction types.

For atom⋯atom bond indices, the average covalency percentages (light-blue column) are estimated for 42 HBs, 31 XBs and 33 YBs. Based on these average values, it is clearly seen that these intermolecular interactions are dominated by ionicity, as the ionic indices are consistently higher than the covalent indices. The average %ionicity values observed for different classes of interaction are 70% for HBs, 78% for XBs and 62% for YBs. It must be cautioned that these averaged percentage values need not be taken as typical of each interaction type, as the values of the interactions within a given type vary over a wide range (as shown in Table S1 in the supporting information, and Tables 1 ▸ and 2 ▸). The observation that these interactions are more ionic than covalent in their bond-order components supports the reported experimental and computational studies which suggest their predominant electrostatic nature, based on electrostatic potentials, experimental deformation densities (Grabowski, 2011 ▸; Edwards *et al.*, 2017 ▸; Bui *et al.*, 2009 ▸; Mani & Arunan, 2013 ▸) *etc*.

For molecule⋯molecule bond indices, these percentages (dark-blue column) are averaged with data sets of 41, 31 and 25 dimers for HBs, XBs and YBs, respectively. We observe that the covalent percentage is higher for mol­ecule⋯mol­ecule interactions than for atom⋯atom interactions, as presented in Tables 1 ▸ and 2 ▸. It may be noted that the %ionicity and %covalency values vary significantly for different mol­ecular dimers of the same interaction type, as found in Tables 1 ▸ and 2 ▸, especially when the total bond orders are very small (*i.e.* for very weak interactions). Hence, we recommend drawing only qualitative conclusions from these trends. An important insight is that there is a balance of both ionic and covalent contributions in most of the intermolecular interactions.

Further, we analysed the values of the ionic and covalent bond indices. As expected, the molecule⋯molecule covalent indices are higher than the corresponding atom⋯atom covalent indices in all 106 dimers. This is because the mol­ecule⋯mol­ecule indices account for the shared electron population from all the atoms in a molecular dimer. However, this is not the case with the ionic bond indices. The atom⋯atom ionic bond indices are higher than corresponding molecule⋯molecule indices for 34 out of 106 dimers. Out of these 34 dimers, there are 15 dimers with HBs and four dimers with YBs, for which the molecule⋯molecule ionic indices are reduced to zero. This is due to the net cancellation of electronic transfer occurring in opposite directions.

**Figure 4:**
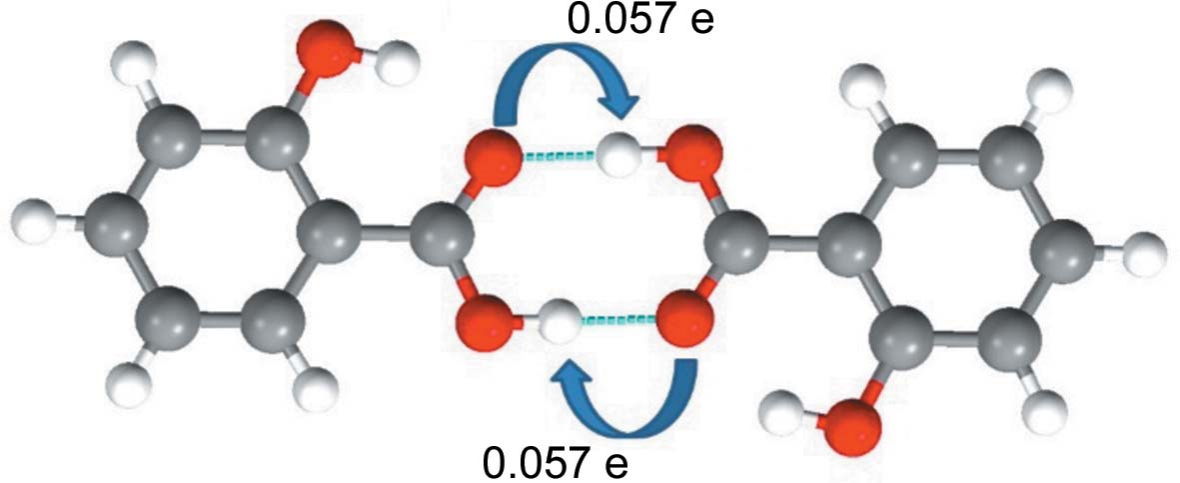
The reverse charge transfer and complete cancellation of mol­ecule⋯mol­ecule ionicity in a carb­oxy­lic acid dimer (SALIAC12) related by inversion symmetry. The values of charge (electron) transfer along the (O—H⋯O) hydrogen bonds are given, with their directions. Grey atoms are C, red O and white H.

**Figure 5:**
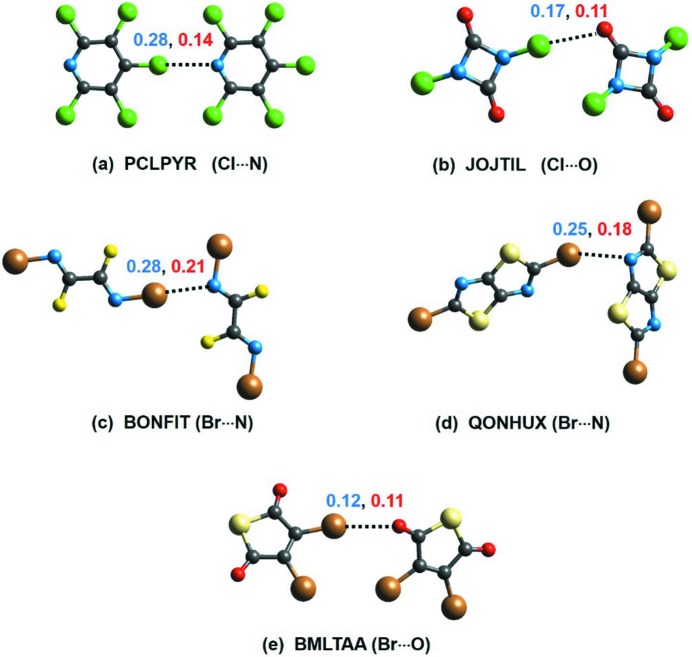
The molecular structures of the five dimers that exhibit atom⋯atom bond indices (blue) higher than their molecule⋯molecule bond indices (red). The CSD refcodes and interaction type are denoted below each dimer. Grey atoms are C, green Cl, blue N, red O, gold Br and yellow S.

A typical example of such a case is shown in Fig. 4 ▸, where a symmetric carboxylic acid dimer forms two strong O—H⋯O HBs which are related by inversion. This leads to the mutual cancellation of effective charge transfer through these HBs and results in a net molecule⋯molecule ionicity bond index of zero. This is significant as it implies that, in such cases, molecule⋯molecule bond indices are devoid of the ionicity component and hence can be biased. Such reverse charge transfer also leads to partial cancellation of the ionicity. This may also be conceived as the contribution of ‘ionic antibonding orbitals’ (as shown in Fig. 2 ▸). As a result, mol­ecule⋯mol­ecule ionic indices in 15 examples analysed in this study are found to be lower than the corresponding atom⋯atom values (see Table S2 in the supporting information). In the five dimers shown in Fig. 5 ▸, such partial cancellation of the ionicity leads to molecule⋯molecule total bond indices lower than the corresponding atom⋯atom bond indices.

These examples clearly demonstrate that, when we consider certain aspects of interactions such as ionicity or charge transfer, localized atom⋯atom considerations or localized moiety–moiety estimates are indeed necessary, as opposed to molecule⋯molecule estimates for the whole molecule.

### 3.4. Estimating charge transfer in hydrogen-bonding and σ-hole interactions *via* Hirshfeld atom partitioning   

A widely accepted picture of intermolecular interactions such as HBs, XBs and YBs is that of an *n* → σ* interaction, where often the occupied nonbonding molecular orbital (or NBMO) of the bond acceptor (*A*) is directed towards the σ* (*D*—*X*) antibonding molecular orbital. Hence, these inter­actions may be associated with an electron transfer from bond acceptor to bond donor (*A* → *X*, or nucleophile to electrophile). To probe this, we have analysed the Hirshfeld charge for a data set of 194 systems (97 dimers and the corresponding 97 monomers). The change in Hirshfeld charges on the acceptor and donor atoms from the monomer state to the corresponding dimer state is estimated as the charge transfer due to the intermolecular interaction. Hence, we calculate the Hirshfeld charge transfer for acceptor and donor atoms as follows
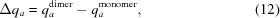
where 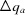 is the difference in Hirshfeld charge, and 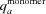 and 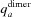 refer to the Hirshfeld charge on the acceptor and donor atoms for the monomer and dimer, respectively. The charge transfer is evident from the Δ*q* values, which are in the ranges 0.023–0.135 a.u. (a.u. = atomic unit) for strong HBs, 0.004–0.046 a.u. for weak HBs, 0.001–0.035 a.u. for XBs and 0.001–0.043 a.u. for YBs. These values of the charge transfer compare well with those previously reported for such interactions (Legon, 2010 ▸; Řezáč & Lande, 2017 ▸).

The full set of Δ*q* results for donor and acceptor atoms is presented in Tables S3–S5 of the supporting information. We can clearly see that the charges on acceptor atoms are increased after forming an interaction, with just a few exceptional cases (one out of 30 in XBs and five out of 33 in YBs). In contrast, the charges on donor atoms are decreased after forming a bonding interaction, and the exceptions are two out of 42 in HBs, eight out of 30 in XBs and 13 out of 33 in YBs. This means that, as a general trend, there is electron transfer from *A* → *X*, *i.e.* from bond acceptor to donor. These results substantiate the *n* → σ* charge-transfer picture for HBs, XBs and YBs.

**Figure 6:**
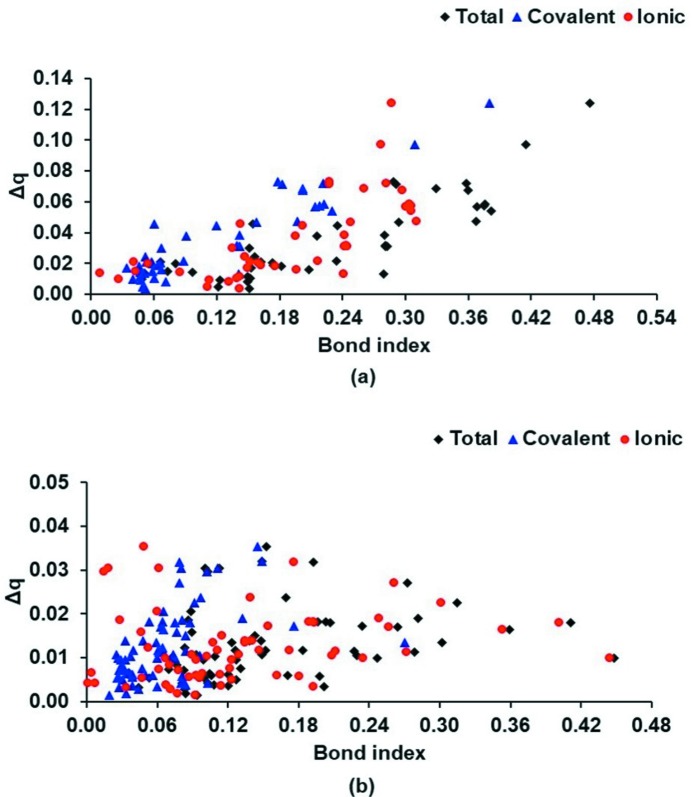
Atom⋯atom RGBIs, including covalent, ionic and total, *versus* the difference in Hirshfeld charge of the acceptor atoms (absolute values in a.u.) between dimers and monomers (Δ*q*) for (*a*) hydrogen bonds, and (*b*) halogen- and chalcogen-bonding interactions. For ionic bond indices, we plot the absolute values.

Fig. 6 ▸ shows the correlations of the covalent, ionic (absolute values) and total atom⋯atom bond indices with the changes in Hirshfeld charge (Δ*q_a_*) for HBs, XBs and YBs. For HBs (Fig. 6 ▸ *a*), we observe a correlation between the bond indices and charge transfer in terms of a change in the Hirshfeld charge. This demonstrates the role of charge transfer in HBs, although a clear correlation between charge transfer and ionic bond order is not observed. In particular, for halogen- and chalcogen-bonding interactions (Fig. 6 ▸ *b*), we find very poor correlation between RGBI values and Δ*q* values. The reason for this may be that, in many of these molecular dimers, the atoms involved in the interactions (atoms *X* and *A* in a *D*—*X*⋯*A* interaction) possess significant partial opposite charges, even in the monomer state (as given in Tables S3–S5), and the charge transfer Δ*q* may be only a component of the inter­action. Another possible origin of this discrepancy could be the difference in the definitions of atoms used in the calculation of Hirshfeld charge and in the RGBI scheme for transfer population (in the calculation of ionic bond order).

### 3.5. Distance and directional dependence of bond indices   

**Figure 7:**
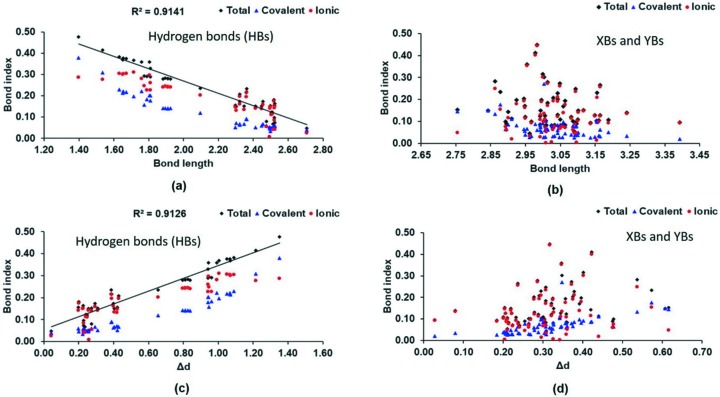
Atom⋯atom RGBIs *versus* distance and the van der Waals interpenetration (Δ*d*) for (*a*) and (*c*), respectively, hydrogen bonds (HBs), and (*b*) and (*d*), respectively, halogen- and chalcogen-bonding interactions (XBs and YBs, respectively). For ionic bond indices, we plot the absolute values. Correlation coefficients *R* ^2^ are given for (*a*) and (*c*) HBs.

Fig. 7 ▸ shows the correlations of the covalent, ionic (absolute values) and total atom⋯atom bond indices with the inter­action distances (*d*) and the interpenetration of the van der Waals (vdW) spheres [the difference between the interaction distance and the sum of the vdW radii (Δ*d*) for HBs, XBs and YBs].

For the HBs (Fig. 7 ▸ *a*), we observe a rough distance dependence with a correlation coefficient *R* ^2^ = 0.91 for the total bond indices. A similar trend in the opposite direction is observed for Δ*d*.

Such a trend is not found for the halogen and chalcogen interactions (Fig. 7 ▸ *b*). A wide distribution of bond indices for a small window of interaction distances suggests a higher directional dependence of XBs and YBs compared with the distance dependence.

**Figure 8:**
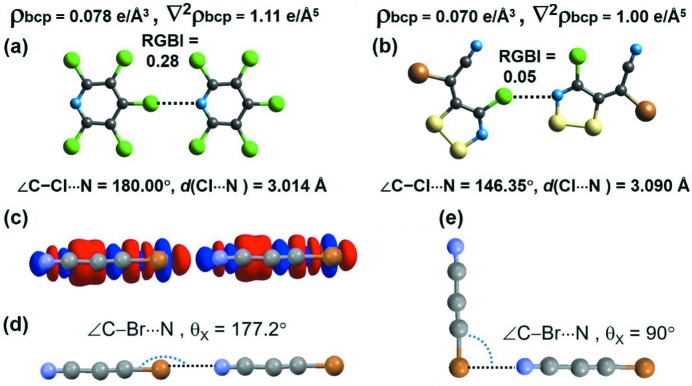
Atom⋯atom RGBIs for (*a*) the PCLPYR dimer and (*b*) the XIZPON dimer that exhibit very similar interaction distances and different interaction angles. The interaction regions are marked with their RGBIs and the AIM topological parameters evaluated at the bcps. (*c*) The deformation electron-density map (0.005 a.u. surface) plotted for the linear dimer shows the effective interaction between the charge-depleted region of Br and the charge-concentrated region of N (lone-pair density). (*d*) The linear ∠C—Br⋯N experimental geometry in BCACEN (θ_*x*_ = 177.2°). (*e*) The perpendicular ∠C—Br⋯N hypothetical geometry in BCACEN (θ_*x*_ = 90°). Grey atoms are C, green Cl, blue N, gold Br and yellow S.

An intriguing example where atom⋯atom RGBIs vary significantly with the difference in interaction angles (*D*—*X*⋯*A*) despite the very similar interaction distances (*d*) is presented in Fig. 8 ▸. We see that the RGBIs of the Cl⋯N interaction for the PCLPYR dimer and for XIZPON are 0.28 (C—Cl⋯N = 180.00°, *d* = 3.014 Å) and 0.05 (C—Cl⋯N = 146.35°, *d* = 3.090 Å), respectively. This further confirms the higher directionality often associated with σ-hole interactions such as XBs and YBs compared with HBs. We also compared the atoms in molecules (AIM) topological properties of electron density (ρ) and its Laplacian (∇^2^ρ) at the bond-critical points (bcps) of these two interacting dimers. It is to be noted that the topological parameters for the Cl⋯N interaction in the PCLPYR dimer and in XIZPON are very similar, despite the remarkable difference in their XB angle (C—Cl⋯N). This suggests that bond-order estimations will be more sensitive to directional variations in intermolecular interactions than will electron-density topological parameters.

In order to verify this trend and the effect of interaction angles on bond orders, we need to have examples of dimers that show very similar interaction distances and very different angles. Unfortunately, we do not have such examples in the series of compounds studied in this paper, apart from the two examples presented in Figs. 8 ▸(*a*) and 8 ▸(*b*). Hence, we generated hypothetical molecular dimers, varying the interaction angles (*D*—*X*⋯*A*), for the linear molecules NC—CC—Br and NC—CC—Cl (CSD refcodes BCACEN and CCACEN), which exhibit Br⋯N and Cl⋯N interactions, keeping the Br⋯N and Cl⋯N distances fixed. The *D*—*X*⋯*A* angle is significant, as a nearly 180° angle is directly linked to the effective *n* → σ* interaction. Starting from the linear geometries in the crystal structures of BCACEN and CCACEN, we varied the *D*—*X*⋯*A* angle up to 90° (Figs. 8 ▸ *d* and 8 ▸ *e*). It may be noted that the most effective *n* → σ* interaction geometry also corresponds to the interaction between the charge-depleted (CD) region on the halogen atom and the charge-concentrated (CC) region on the nitro­gen atom (as seen in Fig. 8 ▸ *c*). Hence, a *D*—*X*⋯*A* angle of 90° corresponds to a lone-pair–lone-pair repulsion and can be destabilizing. These trends are clearly observed when we evaluate the interaction energies of these hypothetical dimers evaluated at the M062x/Def2TZVP level (Table 4 ▸). Interestingly, the decrease in stability of these dimers from linear to perpendicular geometries reflects well in their RGBI values, and notably in the ionic component of the RGBIs. However, the AIM topological parameters show the opposite trend (Table 4 ▸). These observations further verify that RGBI values are more sensitive to the directionality of intermolecular interactions than the AIM parameters.

### 3.6. Correlation between bond indices and intermolecular interaction energies   

**Figure 9:**
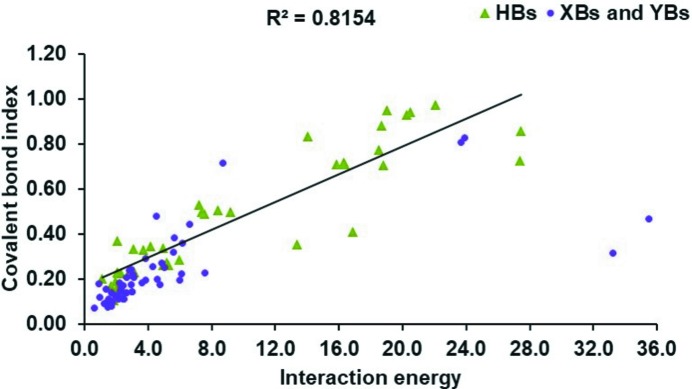
Molecule⋯molecule covalent RGBIs *versus* intermolecular interaction energies (in kcal mol^−1^ calculated at the M062x/def2TZVP level; 1 kcal mol^−1^ = 4.184 kJ mol^−1^) for hydrogen bonds (HBs), halogen-bonding interactions (XBs) and chalcogen-bonding interactions (YBs). The correlation coefficient *R* ^2^ is also given.

As interaction energy is a molecule⋯molecule descriptor, we restrict this discussion to the correlations between mol­ecule⋯mol­ecule RGBI and intermolecular interaction energies calculated at the M062x/def2TZVP level. The full set of results is presented in Tables S6–S8 of the supporting information. Since the ionic indices can be affected by a reverse charge-transfer contribution as detailed in a previous section (see Section 3.3), here we discuss the correlations between covalent molecule⋯molecule bond indices. A rough correlation (Fig. 9 ▸) is observed between covalent bond indices and interaction energies, with molecular dimers linked by HBs clearly clustered away from XBs and YBs. This suggests that RGBI values can indeed be used as indicators of interaction strength.

### 3.7. Testing the conservation of bond orders in the interaction region   

Finally, we set out to test the idea of bond-order conservation in the interaction region, as proposed recently by Shahi & Arunan (2014 ▸). They showed that the formation of a *D*—*X*⋯*A* intermolecular interaction results in a reduction in the bond order of the *D*—*X* covalent bond and this reduction is comparable in magnitude to the bond order of the *X*⋯*A* interaction. This proposition refers to the weakening of a chemical bond on the formation of an intermolecular interaction. Recently, Thomas and co-workers showed the weakening of the Se—N bond in the antioxidant ebselen caused by intermolecular Se⋯O chalcogen bonding, which could be related to the bond-cleavage mechanism in its drug action (Thomas *et al.*, 2015 ▸).

**Figure 10:**
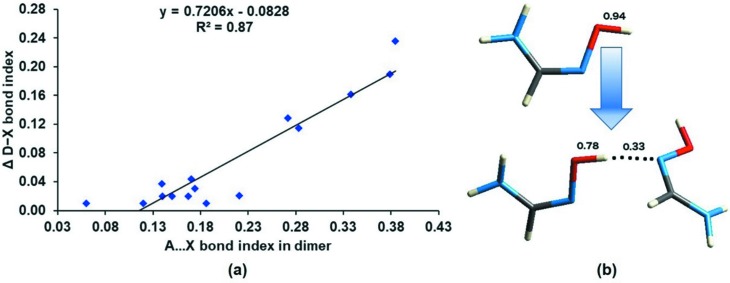
(*a*) The change in RGBI values of the bond donor (Δ*D*—*X*) from monomer molecule to dimer *versus* the RGBIs of the *A*⋯*X* interactions. (*b*) An illustrative example of *D*—*X* bond weakening upon formation of a *D*—*X*⋯*A* interaction; in this example, the RGBI value of the O—H bond decreases from 0.94 to 0.78 when an O—H⋯N hydrogen bond is formed. The correlation coefficient *R* ^2^ is also given for (*a*).

We tested bond-order conservation using atom⋯atom RGBIs in a selected set of 15 dimers including hydrogen, halogen and chalcogen interactions. These dimers were specifically selected from the 97 dimers studied here based on their small molecular size, as the calculations involved RGBI estimations on optimized geometries of both monomers and dimers. Further, the change in the total RGBI values of the *D*—*X* bonds from monomer molecule to dimer (Δ*D*—*X*) were compared with the RGBIs of the *A*⋯*X* interactions (Fig. 10 ▸ *a*). Although we found an interesting trend of *D*—*X* bond-order reduction (in terms of RGBI values) upon the formation of interactions, quantitative trends between RGBI (Δ*D*—*X*) and RGBI (*A*⋯*X*) do not show bond-order conservation. The full set of results is presented in Table S9 of the supporting information. A correlation coefficient *R* ^2^ = 0.87 with a slope of 0.72 for these 15 dimers shows that ‘bond-order conservation’ is not very well obeyed. Nevertheless, it shows that *an interaction formed is a bond weakened.* Our observations further underscore that characteristic trends in localized atom⋯atom properties such as bond order are associated with intermolecular interactions in crystals.

An example where the RGBI of a *D*—*X* bond reduces upon HB formation is shown in Fig. 10 ▸(*b*) for the dimer in the crystal structure FORAMO01. We can see that the RGBI in the bond donor (O—H) decreases from 0.94 in the monomer to 0.78 in the dimer. This reduction compares well with the bond order of the interaction formed (O—H⋯N) in the dimer.

## 4. Conclusions   

In summary, the RGBI values estimated in this study for the major classes of noncovalent interaction place them on a scale representing their relative strengths, in conjunction with a chemist’s notion of bonds. These bond orders may be superior to the electron-density topological parameters usually evaluated at bond-critical points, as they account for both electron sharing and charge transfer separately. Moreover, we have shown that the trends in angular dependence of the inter­action strengths are better reflected in their RGBI values than in the AIM topological parameters. We establish a clear trend of electron transfer from bond acceptor to donor (*i.e.* *A*→ *X*—*D* for HBs, XBs and YBs). Estimates of atom⋯atom and molecule⋯molecule bond orders and their ionic and covalent components clearly establish the occurrence of reverse charge transfer, either completely (with inversion symmetry between the interacting molecules) or partially (*via* ionic antibonding orbitals), in a series of examples. These results emphasize the significance of considering localized atom⋯atom interactions along with the holistic molecule⋯molecule picture for understanding supramolecular assembly in crystals. Further, the strong directionality associated with σ-hole interactions such as halogen and chalcogen bonds and the weakening of *D*—*X* covalent bonds upon the formation of such interactions are clearly evident from our RGBI estimates. This study also opens up the possibility of deriving RGBI values from quantum crystallographic X-ray wavefunctions (or ‘experimental wavefunctions’). Our future efforts will focus on the accurate determination of experimental bond orders, which will provide insight into intermolecular interactions and bonds in crystalline solids.

## Supplementary Material

Additional tables and figures. DOI: 10.1107/S2052252518010758/yc5015sup1.pdf

## Figures and Tables

**Table 1:** Atom⋯atom and molecule⋯molecule RGBIs, covalent index (*c*), ionic index (*i*) and total bond index (τ) for halogen-bonding interactions (*X*⋯*A*, *X* = Cl, Br, *A* = N, O) The distances *d* and interpenetration of the van der Waals spheres (Δ*d*) are given in ångström. Single- and multiple-contact interactions in the dimers are marked with the superscripts s and m, respectively.

*X*⋯*A*	*d*(*X*⋯*A*)	Δ*d*(*X*⋯*A*)	Atom⋯atom indices				Molecule⋯molecule indices			
CSD refcode	(Å)	(Å)	*c*	*i*	τ	%*c*	*c*	*i*	τ	%*c*
Cl⋯N										
CCACENN^s^	2.984	0.316	0.06	0.44	0.45	1.79	0.14	−0.60	0.61	5.52
DESKER01^s^	2.954	0.346	0.07	0.35	0.36	3.31	0.18	−0.58	0.61	8.69
NABZAS^s^	3.092	0.208	0.04	−0.09	0.09	16.45	0.11	0.10	0.15	57.89
PCLPYR^s^	3.014	0.286	0.06	0.27	0.28	4.87	0.14	−0.02	0.14	97.39
VUGSIZ^s^	3.100	0.200	0.04	−0.10	0.11	11.92	0.10	0.21	0.23	17.68
PALPAV^m^	3.097	0.203	0.04	0.00	0.04	98.61	0.17	0.07	0.18	84.87
XIZPON^m^	3.090	0.210	0.03	−0.05	0.05	23.21	0.16	−0.28	0.32	24.01
Cl⋯O										
BEDMONN^s^	3.033	0.237	0.03	−0.08	0.08	16.03	0.08	−0.18	0.19	15.52
BZQDCL11^s^	3.056	0.214	0.03	−0.08	0.08	10.90	0.09	0.06	0.11	70.69
CORDUI^s^	3.047	0.223	0.03	−0.07	0.07	14.15	0.07	0.11	0.13	28.29
DCLBZQ20^s^	3.006	0.264	0.03	−0.07	0.08	14.86	0.09	0.08	0.13	55.12
IRUFEH01^s^	2.966	0.304	0.03	−0.09	0.10	11.00	0.11	−0.40	0.42	6.84
JOJTIL^s^	2.948	0.322	0.06	−0.16	0.17	10.76	0.11	−0.01	0.11	99.37
RUBSUD^s^	2.949	0.321	0.04	−0.11	0.12	10.47	0.10	0.22	0.24	17.48
TCACAD01^s^	3.029	0.241	0.03	−0.06	0.07	21.01	0.09	0.08	0.12	54.42
GEXWUB^s^	3.002	0.268	0.03	−0.10	0.10	7.47	0.08	0.07	0.11	52.07
PEPFUL^s^	2.962	0.308	0.03	−0.03	0.04	40.53	0.09	−0.06	0.10	66.68
Br⋯N										
BCACENN^s^	2.978	0.422	0.09	0.40	0.41	4.53	0.19	−0.46	0.50	14.78
BONFIT^s^	2.863	0.537	0.13	0.25	0.28	22.17	0.21	−0.05	0.21	95.10
QONHUX^s^	3.093	0.307	0.07	0.24	0.25	9.23	0.16	−0.07	0.18	82.95
RIRFOON^s^	3.164	0.236	0.06	0.26	0.26	5.61	0.14	−0.22	0.26	27.91
KUYCUD^s^	2.999	0.401	0.09	0.30	0.32	8.40	0.24	0.46	0.51	21.20
Br⋯O										
BMLTAAN^s^	3.082	0.288	0.04	−0.11	0.12	10.36	0.11	0.00	0.11	99.81
CIRSONN^s^	3.149	0.221	0.03	−0.12	0.13	5.18	0.12	0.09	0.15	62.28
JEVVOW^s^	2.895	0.475	0.07	0.06	0.09	54.37	0.14	−0.08	0.16	77.75
VAQXUG^s^	3.160	0.210	0.03	−0.10	0.10	10.75	0.12	−0.04	0.13	92.26
VEWTAU^s^	2.893	0.477	0.06	0.07	0.10	42.44	0.14	0.00	0.14	99.99
VEWTEY^s^	3.164	0.206	0.03	−0.12	0.13	5.82	0.10	−0.16	0.19	28.93
VITVEZ^s^	3.063	0.307	0.04	−0.07	0.08	27.52	0.11	0.14	0.18	39.26
WADFIR^s^	3.009	0.361	0.06	0.05	0.08	60.60	0.13	0.16	0.21	38.82
ACETBR02^m^	2.755	0.615	0.14	0.05	0.15	89.93	0.32	−0.66	0.73	19.09

**Table 2:** Atom⋯atom and molecule⋯molecule RGBIs, covalent index (*c*), ionic index (*i*) and total bond index (τ) for chalcogen-bonding interactions (*Y*⋯*A*, *Y* = S, Se, *A* = N, O) The distances *d* and inter-penetration of the van der Waals spheres (Δ*d*) are given in ångström. Single- and multiple-contact interactions in the dimers are marked with the superscript s and m, respectively (and m* for those that have two identical interactions within one dimer due to symmetry).

*Y*⋯*A*	*d*_(*Y*⋯*A*)_	Δ*d*_(*Y*⋯*A*)_	Atom⋯atom indices				Molecule⋯molecule indices			
CSD refcode	(Å)	(Å)	*c*	*i*	τ	%*c*	*c*	*i*	τ	%*c*
S⋯N										
CEBYUD^s^	3.050	0.300	0.08	0.07	0.11	60.95	0.14	0.16	0.22	45.15
QOBFUI^s^	2.992	0.358	0.08	0.03	0.09	89.27	0.21	−0.39	0.44	21.44
SAZCEC^s^	3.096	0.254	0.08	0.05	0.09	73.53	0.18	0.32	0.37	24.27
GEDHAY^m^	2.910	0.440	0.11	0.02	0.11	97.25	0.36	0.23	0.43	70.46
GEDHAY^m^	3.086	0.264	0.08	−0.06	0.10	63.18				
IFULUQ04^m^	3.006	0.344	−0.06	−0.11	0.13	23.20	0.83	1.24	1.49	30.60
WASHEE^m^	3.003	0.347	0.27	−0.13	0.30	80.08	0.81	−1.28	1.52	28.31
WASHEE^m^	2.992	0.358	−0.06	−0.11	0.12	23.20				
WUXPAG^m^	3.008	0.342	0.10	0.00	0.10	99.98	0.31	−0.15	0.35	81.77
WUXPAG^m^	3.024	0.326	0.08	−0.01	0.08	99.28				
S⋯O										
PAFVEY^s^	3.029	0.291	0.07	−0.19	0.21	13.09	0.18	0.27	0.32	29.95
WOCQEK^s^	2.900	0.420	0.08	−0.12	0.14	34.90	0.19	0.73	0.76	6.56
IMTAZON^s^	3.097	0.223	0.05	−0.10	0.12	19.03				
ADOFEF^m^*	3.101	0.219	0.05	−0.14	0.15	10.52	0.25	0.00	0.25	100.0
ADOFEF^m^*	3.241	0.079	0.03	−0.13	0.14	5.64				
MAVRAD^m^	3.042	0.278	0.06	−0.19	0.20	8.79	0.22	0.50	0.55	16.67
MEHNIY^m^	3.042	0.278	0.05	−0.12	0.13	12.95	0.47	0.37	0.60	61.43
NAHMUE^m^	2.945	0.375	0.06	−0.19	0.20	10.09	0.18	0.46	0.49	13.64
NAHMUE^m^	2.995	0.325	0.05	−0.19	0.20	7.28				
PUDMUW^m^	2.993	0.327	0.07	−0.13	0.15	24.26	0.24	0.39	0.46	27.66
PUDMUW^m^	3.136	0.184	0.03	−0.09	0.09	7.53				
QELQEE^m^	3.013	0.307	0.06	−0.17	0.18	12.21	0.17	0.29	0.34	26.10
QELQEE^m^	3.117	0.203	0.04	−0.15	0.15	6.83				
ZAVHEJ^m^*	2.924	0.396	0.08	−0.18	0.20	16.93	0.25	0.00	0.25	100
Se⋯N										
BESEAZ01^s^	3.155	0.295	0.10	−0.21	0.23	17.54	0.22	0.34	0.40	29.56
FENFION^s^	3.154	0.296	0.10	−0.01	0.10	98.05	0.29	0.74	0.79	13.34
WERYAT^s^	2.843	0.607	0.15	0.00	0.15	99.98	0.48	0.24	0.53	79.09
NECZUQ^m^*	2.877	0.573	0.18	−0.15	0.23	56.55	0.71	0.00	0.71	100.00
SECNBZ^m^	3.058	0.392	0.10	0.14	0.17	32.62	0.44	−0.29	0.52	70.26
Se⋯O										
BOJCOS^m^*	3.042	0.378	0.08	−0.21	0.23	13.69	0.27	0.00	0.27	100.00
LEDGAD^m^	3.188	0.232	0.05	−0.09	0.11	22.64	0.22	0.05	0.23	94.68
LEDGAD^m^	3.393	0.027	0.20	−0.09	0.09	4.29				
LEVJOM^m^	3.049	0.371	0.08	−0.26	0.27	8.43	0.20	0.86	0.88	5.04
MUSCIM^m^	3.064	0.356	0.08	−0.18	0.19	16.60	0.38	0.60	0.71	28.71

**Table 3:** The covalent and ionic Roby–Gould populations for the three main paired orbitals of bonding and antibonding modes for the Cl⋯N interaction in the CCACEN dimer The angle (θ) values for each pair are given. The covalent (*c*) and ionic (*i*) parameters are also given for each pair of orbitals.

	Covalent population			Ionic population		
Angle θ (°)	Bonding	Antibonding	*c*	Bonding	Antibonding	*i*
83	1.870	1.797	0.036	1.932	1.735	0.098
89	1.694	1.671	0.011	1.411	1.954	−0.271
89	1.694	1.670	0.011	1.410	1.954	−0.271

**Table 4:** The angle dependence of RGBI values, interaction energies and AIM topological parameters for Cl⋯N and Br⋯N halogen-bonded dimers with hypothetical *D*—*X*⋯*A* angle geometries, except the first angle of each dimer has an experimental angle geometry

*X*⋯*A*	C—*X*⋯N angle θ_*x*_ (°)	Covalent index (*c*)	Ionic index (*i*)	Total RGBI (τ)	Interaction energy (kcal mol^−1^)	ρ_bcp_ (e Å^−3^)	∇^2^ρ_bcp_ (e Å^−5^)
Cl⋯N							
CCACEN	178.30	0.06	0.44	0.45	−2.99	0.074	1.148
	155.00	0.05	0.44	0.44	−2.21	0.078	1.177
	135.00	0.04	0.43	0.43	−0.91	0.085	1.229
	115.00	0.03	0.41	0.42	0.08	0.090	1.275
	90.00	0.03	0.40	0.40	−0.06	0.092	1.291
Br⋯N							
BCACEN	177.20	0.09	0.40	0.41	−3.88	0.090	1.301
	155.00	0.08	0.40	0.40	−2.55	0.096	1.323
	135.00	0.06	0.38	0.39	−0.51	0.105	1.377
	115.00	0.04	0.37	0.37	−0.51	0.111	1.427
	90.00	0.04	0.35	0.36	1.39	0.113	1.437
