# Supplementary material for: Bond orders for intermolecular interactions in crystals: charge transfer, ionicity and the effect on intramolecular bonds
Source: IUCrJ. 2018 Aug 29;5(Pt 5):635–46. doi: 10.1107/S2052252518010758 (PMC6126646; doi:10.1107/S2052252518010758)
Supplement: Supplementary file 1 [file m-05-00635-sup1.pdf]

IUCrJ

Volume 5 (2018)

Supporting information for article:

Bond orders for intermolecular interactions in crystals: Charge transfer, ionicity and the effect on intramolecular bonds

Khidhir Alhameedi, Amir Karton, Dylan Jayatilaka and Sajesh P. Thomas

Supporting Information

Bond orders for intermolecular interactions in crystals: Charge transfer, ionicity and the effect on intramolecular bonds

Khidhir Alhameedi,*[ab] Amir Karton,[a] Dylan Jayatilaka,*[a] and Sajesh P. Thomas*[a]

[a] School of Molecular Sciences, University of Western Australia, 35 Stirling Highway, Perth 6009, Australia.

[b] Department of Chemistry, College of Education for Pure Science, University of Karbala, Karbala, Iraq.

E-mail:

sajeshpthomas@gmail.com

khidhir.alhameedi@research.uwa.edu.au

• Please note that the all output files are deposited for open access under figshare.com website, see the link: https://figshare.com/s/538fcbb3898fc764a21b.

Contents

S1 Roby-Gould bond indices for hydrogen bonds **1**

S2 Atom···atom ionic index versus molecule···molecule ionic index **2**

S3 Hirshfeld atom charges **3**

S4 Interaction energies for hydrogen bonds, halogen bonds and chalcogen bonds **7**

S5 Testing the conservation of Roby-Gould bond bond order **11**

S6 The angle dependence of RGBI values **12**

S7 Percentage of covalency **14**

S8 Molecular structures of selected dimers **15**

S8.1 Hydrogen bonds . . . 16

S8.1.1 C–H···N interaction . . . 16

S8.1.2 N–H···N interactions . . . 21

S8.1.3 O–H···N interactions . . . 23

S8.1.4 C–H···O interactions . . . 25

S8.1.5 N–H···O interactions . . . 28

S8.1.6 O–H···O interactions . . . 31

S8.2 Halogen bonds . . . 34

S8.2.1 Cl···N interactions . . . 34

S8.2.2 Cl···O interactions . . . 37

S8.2.3 Br···N interactions . . . 41

S8.2.4 Br···O interactions . . . 43

S8.3 Chalcogen bonds . . . 47

S8.3.1 S···N interactions . . . 47

S8.3.2 S···O interactions . . . 51

S8.3.3 Se···N interactions . . . 57

S8.3.4 Se···O interactions . . . 60

S1 Roby-Gould bond indices for hydrogen bonds

Table S1: Atom$\cdots$atom and molecule$\cdots$molecule Roby-Gould bond indices, covalent index (c), ionic index (i), and total bond index (τ), for hydrogen bonds (D−H$\cdots$A, D= C, N, O, A= N, O). The distances d and the inter-penetration of van der Waals spheres (Δd) are given in Angstrom (Å). The single and multipliable contact interactions in dimers are marked with superscript s and m (and $m*$ for two identical interactions within one dimer).

D−H$\cdots$A	$d_{(H\cdots A)}$	$\Delta d_{(H\cdots A)}$	Atom-atom indices				molecule-molecule indices			
CSD code	/Å	/Å	c	i	τ	%c	c	i	τ	%c
C−H$\cdots$N										
CYACHZ01^s	2.476	0.274	0.06	0.05	0.08	53.75	0.23	0.79	0.83	7.75
DMSDIM01^s	2.522	0.228	0.05	0.08	0.10	23.19	0.16	0.06	0.17	87.53
MEADEN02^s	2.524	0.226	0.06	0.15	0.16	12.49	0.19	0.52	0.56	11.75
TRAZOL02^s	2.346	0.404	0.07	0.13	0.15	19.80	0.22	1.12	1.14	3.79
BUGKIX01^s	2.523	0.227	0.06	0.04	0.07	65.47	0.16	0.78	0.80	3.87
BUGKIX01^s	2.321	0.429	0.07	0.20	0.21	10.56	0.28	0.44	0.52	28.21
CYACHZ01^m	2.509	0.241	0.05	0.11	0.12	15.14	0.17	0.72	0.74	5.21
NURWOM02^{m*}	2.495	0.255	0.05	0.14	0.15	9.63	0.33	0.00	0.33	100.00
NURWOM02^m	2.456	0.294	0.07	0.16	0.17	14.76	0.33	0.12	0.35	89.18
TEPNIT04^{m*}	2.503	0.247	0.05	0.11	0.12	16.18	0.29	0.00	0.29	100.00
XEHMOM01^m	2.513	0.237	0.05	0.04	0.07	62.01	0.37	0.87	0.94	15.53
XEHMOM01^m	2.492	0.258	0.05	0.01	0.05	97.42	0.22	0.94	0.97	5.35
XEHMOM01^m	2.707	0.043	0.04	−0.03	0.05	69.86				
N−H$\cdots$N										
BAZGOY05^s	2.363	0.387	0.09	0.22	0.23	14.35	0.23	0.49	0.54	18.52
DMSDIM01^s	2.360	0.390	0.09	0.20	0.22	17.75	0.34	0.25	0.42	64.31
FORAMO01^s	2.096	0.654	0.12	0.20	0.23	25.80	0.35	0.25	0.43	65.65
IMAZOL06^s	1.808	0.942	0.18	0.23	0.29	38.04	0.50	0.23	0.55	82.46
TRAZOL02^s	1.788	0.962	0.18	0.23	0.29	39.18	0.51	0.31	0.60	72.60
O−H$\cdots$N										
FORAMO01^s	1.809	0.941	0.20	0.26	0.33	37.44	0.50	0.04	0.50	99.26
GLOXIM11^s	1.805	0.945	0.20	0.30	0.36	31.56	0.53	−0.29	0.61	76.44
MAMPOL02^s	1.758	0.992	0.22	0.28	0.36	38.18	0.49	0.53	0.72	46.52
LOLSUA^m	1.535	1.215	0.31	0.28	0.41	55.51	0.77	−1.19	1.42	29.70
ULAWAF05^m	1.398	1.352	0.38	0.29	0.48	63.64	0.86	−0.51	1.00	73.94
C−H$\cdots$O										
ABINOR04^s	2.519	0.201	0.05	0.18	0.18	6.41	0.23	0.56	0.60	13.95
NBONAN01^s	2.298	0.422	0.05	0.15	0.16	11.10	0.20	1.08	1.10	3.40
POKKAD01^s	2.497	0.223	0.03	0.15	0.15	4.88	0.11	0.33	0.35	9.25
CYACHZ01^{m*}	2.412	0.308	0.05	0.14	0.15	11.93	0.35	0.00	0.35	100.00
FACETA01^{m*}	2.447	0.273	0.06	0.14	0.15	15.61	0.26	0.00	0.26	100.00
GLYGLY04^m	2.521	0.199	0.06	0.14	0.15	14.98	0.41	0.37	0.55	55.32
GLYGLY04^m	2.301	0.419	0.07	0.13	0.15	22.48				
METURA01^m	2.322	0.398	0.06	0.16	0.17	13.24	0.26	0.62	0.67	15.35
N−H$\cdots$O										
BZAMID02^{m*}	1.910	0.810	0.14	0.24	0.28	25.60	0.71	0.00	0.71	100.00
CYURAC12^{m*}	1.774	0.946	0.16	0.25	0.29	28.76	0.83	0.00	0.83	100.00
FACETA01^{m*}	1.909	0.811	0.14	0.24	0.28	24.68	0.71	0.00	0.71	100.00
ULAWAF05^{m*}	1.894	0.826	0.14	0.24	0.28	25.18	0.72	0.00	0.72	100.00
UROXAL01^{m*}	1.924	0.796	0.14	0.24	0.28	25.50	0.71	0.00	0.71	100.00
ZECWUB01^{m*}	1.882	0.838	0.14	0.24	0.28	25.49	0.72	0.00	0.72	100.00
O−H$\cdots$O										
BESKAL10^{m*}	1.715	1.005	0.20	0.31	0.37	28.64	0.88	0.00	0.88	100.00
CUKCAM18^{m*}	1.630	1.090	0.23	0.31	0.38	36.20	0.97	0.00	0.97	100.00
SALIAC12^{m*}	1.651	1.069	0.21	0.30	0.37	33.59	0.95	0.00	0.95	100.00
SUCACB03^{m*}	1.670	1.050	0.22	0.31	0.38	33.72	0.93	0.00	0.93	100.00
ZZZEEU05^{m*}	1.653	1.067	0.22	0.30	0.38	34.80	0.94	0.00	0.94	100.00

S2 Atom··· atom ionic index versus molecule··· molecule ionic index

Table S2: Summarize table of Roby-Gould bond indices, covalent index (c), ionic index (i), and total bond index (τ), for selected 35 examples, where their ionic indices are higher in atom··· atom interaction comparing those in molecule··· molecule interactions.

D–H··· A	$d_{(\mathrm{H}\cdots\mathrm{A})}$	$\Delta d_{(\mathrm{H}\cdots\mathrm{A})}$	Atom-atom indices				molecule-molecule indices			
CSD code	/Å	/Å	c	i	τ	%c	c	i	τ	%c
C–H··· N										
DMSDIM01^{s}	2.522	0.228	0.05	0.08	0.10	23.19	0.16	0.06	0.17	87.53
NURWOM02^{m*}	2.495	0.255	0.05	0.14	0.15	9.63	0.33	0.00	0.33	100.00
NURWOM02^{m}	2.456	0.294	0.07	0.16	0.17	14.76	0.33	0.12	0.35	89.18
TEPNIT04^{m*}	2.503	0.247	0.05	0.11	0.12	16.18	0.29	0.00	0.29	100.00
O–H··· N										
FORAMO01^{s}	1.809	0.941	0.20	0.26	0.33	37.44	0.50	0.04	0.50	99.26
C–H··· O										
CYACHZ01^{m*}	2.412	0.308	0.05	0.14	0.15	11.93	0.35	0.00	0.35	100.00
FACETA01^{m*}	2.447	0.273	0.06	0.14	0.15	15.61	0.26	0.00	0.26	100.00
N–H··· O										
BZAMID02^{m*}	1.910	0.810	0.14	0.24	0.28	25.60	0.71	0.00	0.71	100.00
CYURAC12^{m*}	1.774	0.946	0.16	0.25	0.29	28.76	0.83	0.00	0.83	100.00
FACETA01^{m*}	1.909	0.811	0.14	0.24	0.28	24.68	0.71	0.00	0.71	100.00
ULAWAF05^{m*}	1.894	0.826	0.14	0.24	0.28	25.18	0.72	0.00	0.72	100.00
UROXAL01^{m*}	1.924	0.796	0.14	0.24	0.28	25.50	0.71	0.00	0.71	100.00
ZECWUB01^{m*}	1.882	0.838	0.14	0.24	0.28	25.49	0.72	0.00	0.72	100.00
O–H··· O										
BESKAL10^{m*}	1.715	1.005	0.20	0.31	0.37	28.64	0.88	0.00	0.88	100.00
CUKCAM18^{m*}	1.630	1.090	0.23	0.31	0.38	36.20	0.97	0.00	0.97	100.00
SALIAC12^{m*}	1.651	1.069	0.21	0.30	0.37	33.59	0.95	0.00	0.95	100.00
SUCACB03^{m*}	1.670	1.050	0.22	0.31	0.38	33.72	0.93	0.00	0.93	100.00
ZZZEEU05^{m*}	1.653	1.067	0.22	0.30	0.38	34.80	0.94	0.00	0.94	100.00
Cl··· N										
PCLPYR^{s}	3.014	0.286	0.06	0.27	0.28	4.87	0.14	−0.02	0.14	97.39
BZQDCL11^{s}	3.056	0.214	0.03	−0.08	0.08	10.90	0.09	0.06	0.11	70.69
JOJTIL^{s}	2.948	0.322	0.06	−0.16	0.17	10.76	0.11	−0.01	0.11	99.37
GEXWUB^{s}	3.002	0.268	0.03	−0.10	0.10	7.47	0.08	0.07	0.11	52.07
Br··· N										
BONFIT^{s}	2.863	0.537	0.13	0.25	0.28	22.17	0.21	−0.05	0.21	95.10
QONHUX^{s}	3.093	0.307	0.07	0.24	0.25	9.23	0.16	−0.07	0.18	82.95
RIRFOO^{s}	3.164	0.236	0.06	0.26	0.26	5.61	0.14	−0.22	0.26	27.91
Br··· O										
BMLTAA^{s}	3.082	0.288	0.04	−0.11	0.12	10.36	0.11	0.00	0.11	99.81
CIRSON^{s}	3.149	0.221	0.03	−0.12	0.13	5.18	0.12	0.09	0.15	62.28
VAQXUG^{s}	3.160	0.210	0.03	−0.10	0.10	10.75	0.12	−0.04	0.13	92.26
VEWTAU^{s}	2.893	0.477	0.06	0.07	0.10	42.44	0.14	0.00	0.14	99.99
S··· O										
ADOFEF^{m*}	3.101	0.219	0.05	−0.14	0.15	10.52	0.25	0.00	0.25	100.00
ZAVHEJ^{m*}	2.924	0.396	0.08	−0.18	0.20	16.93	0.25	0.00	0.25	100.00
Se··· N										
NECZUQ^{m*}	2.877	0.573	0.18	−0.15	0.23	56.55	0.71	0.00	0.71	100.00
Se··· O										
BOJCOS^{m*}	3.042	0.378	0.08	−0.21	0.23	13.69	0.27	0.00	0.27	100.00
LEDGAD^{m}	3.188	0.232	0.05	−0.09	0.11	22.64	0.22	0.05	0.23	94.68

S3 Hirshfeld atom charges

In this section we present the values of Hirshfeld atom charges in atomic unit (a.u.), which represents the partial charges of atoms in monomers and dimers. Charges are given for atoms X and A involved in in the hydrogen bonds (Table S3), halogen bonds (Table S4), and chalcogen bonds (Table S5). The difference in Hirshfeld charges for atoms X and A from monomer upon dimer formation are given as Δq.

Table S3: Hirshfeld charges of atoms involved in hydrogen bonds and the charge transfer due to intermolecular interactions.

D–H···A CSD code	A···H	q_A^{dimer} /a.u.	q_H^{dimer} /a.u.	$q_A^{monomer}$ /a.u.	$q_H^{monomer}$ /a.u.	Δq_A /a.u.	Δq_H /a.u.
C–H···N							
CYACHZ01^s	N6–H4	−0.185	0.061	−0.205	0.072	0.020	−0.011
DMSDIM01^s	N3–H3	−0.343	0.043	−0.357	0.054	0.014	−0.011
MEADEN02^s	N7–H5	−0.181	0.030	−0.201	0.050	0.021	−0.020
TRAZOL02^s	N5–H3	−0.132	0.046	−0.162	0.072	0.030	−0.026
BUGKIX01^s	N6–H1	−0.136	0.080	−0.150	0.099	0.015	−0.019
BUGKIX01^s	N5–H1	−0.134	0.078	−0.150	0.099	0.016	−0.021
CYACHZ01^m	N6–H5	−0.195	0.051	−0.205	0.063	0.009	−0.012
NURWOM02^{m*}	N1–H10	−0.126	0.035	−0.137	0.046	0.012	−0.011
NURWOM02^m	N4–H1	−0.119	0.030	−0.139	0.048	0.021	−0.018
TEPNIT04^{m*}	N1–H5	−0.191	0.054	−0.195	0.066	0.005	−0.012
XEHMOM01^m	N5–H1	−0.177	0.055	−0.198	0.068	0.021	−0.013
XEHMOM01^m	N5–H9	−0.184	0.034	−0.198	0.048	0.014	−0.013
XEHMOM01^m	N6–H8	−0.186	0.033	−0.195	0.046	0.010	−0.013
N–H···N							
BAZGOY05^s	N2–H2	−0.143	0.084	−0.165	0.112	0.022	−0.029
DMSDIM01^s	N3–H1	−0.320	0.061	−0.357	0.084	0.038	−0.023
FORAMO01^s	N4–H2	−0.122	0.078	−0.167	0.122	0.044	−0.045
IMAZOL06^s	N4–H1	−0.133	0.086	−0.206	0.156	0.073	−0.070
TRAZOL02^s	N6–H1	−0.137	0.089	−0.208	0.165	0.071	−0.076
O–H···N							
FORAMO01^s	N4–H4	−0.098	0.084	−0.167	0.164	0.068	−0.080
GLOXIM11^s	N3–H2	−0.014	0.092	−0.081	0.176	0.067	−0.085
MAMPOL02^s	N2–H5	−0.101	0.095	−0.173	0.177	0.072	−0.082
LOLSUA^m	N–H1	−0.060	0.093	−0.157	0.045	0.097	0.048
ULAWAF05^m	N1–H1	−0.026	0.103	−0.150	0.237	0.124	−0.135
C–H···O							
ABINOR04^s	O7–H6	−0.204	0.031	−0.223	0.046	0.018	−0.016
NBONAN01^s	O6–H3	−0.219	0.045	−0.243	0.056	0.024	−0.011
POKKAD01^s	O5–H3	−0.278	0.046	−0.295	0.052	0.017	−0.005
CYACHZ01^{m*}	O1–H9	−0.286	0.068	−0.290	0.072	0.004	−0.003
FACETA01^{m*}	O2–H2	−0.325	0.048	−0.335	0.061	0.010	−0.012
GLYGLY04^m	O6–H7	−0.376	0.046	−0.422	0.042	0.046	0.005
GLYGLY04^m	O4–H4	−0.315	0.048	−0.323	0.076	0.008	−0.028
METURA01^m	O4–H4	−0.301	0.034	−0.319	0.049	0.019	−0.016
N–H···O							
BZAMID02^{m*}	O2–H6	−0.290	0.096	−0.321	0.138	0.031	−0.042
CYURAC12^{m*}	O5–H2	−0.258	0.111	−0.305	0.171	0.047	−0.060
FACETA01^{m*}	O1–H7	−0.303	0.100	−0.335	0.144	0.031	−0.043
ULAWAF05^{m*}	O1–H11	−0.282	0.099	−0.313	0.143	0.031	−0.044
UROXAL01^{m*}	O2–H4	−0.356	0.097	−0.394	0.142	0.038	−0.045
ZECWUB01^{m*}	O1–H3	−0.270	0.103	−0.283	0.152	0.013	−0.049
O–H···O							
BESKAL10^{m*}	O3–H11	−0.213	0.133	−0.261	0.199	0.047	−0.067
CUKCAM18^{m*}	O8–H4	−0.225	0.129	−0.278	0.211	0.054	−0.083
SALIAC12^{m*}	O2–H7	−0.212	0.123	−0.269	0.200	0.057	−0.077
SUCACB03^{m*}	O1–H9	−0.237	0.122	−0.295	0.195	0.057	−0.072
ZZZEEU05^{m*}	O7–H4	−0.220	0.121	−0.278	0.200	0.059	−0.079

Table S4: Hirshfeld charges of atoms involved in halogen bonds and the charge transfer due to intermolecular interactions.

X···A CSD code	X···A	q_A^{dimer} /a.u.	q_H^{dimer} /a.u.	$q_A^{monomer}$ /a.u.	$q_H^{monomer}$ /a.u.	Δq_A /a.u.	Δq_H /a.u.
Cl···N							
CCACENN^s	Cl1–N2	0.086	−0.161	0.097	−0.171	−0.011	0.010
DESKER01^s	Cl1–N4	−0.021	−0.066	−0.009	−0.082	−0.012	0.016
NABZAS^s	Cl1–N2	0.005	−0.164	0.019	−0.169	−0.014	0.006
PCLPYR^s	Cl3–N2	−0.008	−0.122	0.002	−0.133	−0.011	0.011
VUGSIZ^s	Cl1–N5	−0.020	−0.139	−0.007	−0.150	−0.013	0.010
PALPAV^m	Cl1–N7	0.000	−0.152	−0.024	−0.159	0.024	0.007
XIZPON^s	Cl1–N3	0.014	−0.115	0.012	−0.120	0.002	0.005
Cl···O							
BEDMON^s	Cl1–O6	0.008	−0.241	0.010	−0.243	−0.002	0.002
BZQDCL11^s	Cl1–O3	−0.011	−0.203	−0.009	−0.210	−0.002	0.007
CORDUI^s	Cl1–O4	−0.054	−0.306	−0.056	−0.316	0.002	0.010
DCLBZQ20^s	Cl1–O4	−0.007	−0.201	−0.005	−0.210	−0.002	0.008
IRUFEH01^s	Cl1–O4	0.006	−0.231	0.015	−0.240	−0.009	0.010
JOJTIL^s	Cl1–O3	0.033	−0.210	0.044	−0.216	−0.011	0.006
RUBSUD^s	Cl1–O9	0.038	−0.153	0.057	−0.157	−0.019	0.004
TCACAD01^s	Cl2–O4	−0.040	−0.249	−0.039	−0.257	0.000	0.007
GEXWUB^s	Cl2–O4	0.003	−0.242	−0.002	−0.247	0.005	0.005
PEPFUL^s	Cl2–O1	0.002	−0.220	0.001	−0.223	0.001	0.003
Br···N							
BCACENN^s	Br1–N2	0.107	−0.155	0.129	−0.173	−0.021	0.018
BONFIT^s	Br2–N3	0.008	−0.107	0.035	−0.126	−0.027	0.019
QONHUX^s	Br2–N3	−0.001	−0.143	0.002	−0.153	−0.003	0.010
RIRFOON^s	Br1–N4	−0.034	−0.128	−0.024	−0.145	−0.010	0.017
KUYCUD^s	Br1–N7	0.028	−0.128	0.047	−0.151	−0.019	0.023
Br···O							
BMLTAA^s	Br1–O3	0.034	−0.198	0.040	−0.204	−0.006	0.006
CIRSONN^s	Br1–O4	−0.037	−0.125	−0.027	−0.135	−0.010	0.009
JEVVOW^s	Br1–O2	−0.037	−0.302	−0.031	−0.322	−0.006	0.020
VAQXUG^s	Br4–O2	0.058	−0.271	0.053	−0.277	0.005	0.006
VEWTAU^s	Br2–O4	−0.023	−0.275	−0.027	−0.269	0.003	−0.006
VEWTEY^s	Br1–O3	−0.025	−0.071	−0.027	−0.076	0.001	0.005
VITVEZ^s	Br2–O6	0.015	−0.176	0.024	−0.179	−0.010	0.003
WADFIR^s	Br1–O4	−0.028	−0.249	−0.023	−0.261	−0.005	0.012
ACETBR02^s	Br1–O	−0.002	−0.229	0.000	−0.265	−0.002	0.035

Table S5: Hirshfeld charges of atoms involved in chalcogen bonds and the charge transfer due to intermolecular interactions.

Y···A CSD code	Y···A	q_A^{dimer} /a.u.	q_H^{dimer} /a.u.	$q_A^{monomer}$ /a.u.	$q_H^{monomer}$ /a.u.	Δq_A /a.u.	Δq_H /a.u.
S···N							
CEBYUD^s	S1–N8	0.224	−0.145	0.231	−0.149	−0.007	0.004
QOBFUI^s	S1–N5	0.144	0.004	0.158	−0.014	−0.014	0.019
SAZCEC^s	S1–N4	0.097	−0.090	0.112	−0.105	−0.016	0.016
GEDHAY^m	S2–N8	0.081	−0.228	0.099	−0.259	−0.018	0.030
GEDHAY^m	S1–N8	0.282	−0.228	0.291	−0.259	−0.009	0.030
IFULUQ04^m	S1–N4	0.147	−0.184	0.120	−0.172	0.027	−0.012
WASHEE^m	S3–N1	0.174	−0.184	0.131	−0.171	0.043	−0.014
WASHEE^m	S4–N1	0.149	−0.184	0.120	−0.171	0.028	−0.014
WUXPAG^m	S2–N6	0.175	−0.176	0.175	−0.180	0.000	0.004
WUXPAG^m	S1–N6	0.192	−0.176	0.152	−0.180	0.040	0.004
S···O							
PAFVEY^s	S1–O4	0.151	−0.232	0.176	−0.250	−0.025	0.018
WOCQEK^s	S1–O4	0.049	−0.220	0.056	−0.242	−0.007	0.022
ADOFEF^{m*}	S4–O1	0.004	−0.291	−0.006	−0.305	0.011	0.014
ADOFEF^{m*}	S3–O1	0.419	−0.291	0.417	−0.305	0.001	0.014
MAVRAD^m	S1–O2	0.088	−0.245	0.089	−0.253	−0.001	0.008
MEHNIY^m	S4–O2	0.071	−0.282	0.047	−0.209	0.024	−0.073
NAHMUE^m	S2–O4	0.037	−0.249	0.049	−0.267	−0.013	0.018
NAHMUE^m	S1–O4	0.038	−0.249	0.049	−0.267	−0.011	0.018
PUDMUW^m	S5–O4	0.026	−0.225	0.033	−0.235	−0.007	0.011
PUDMUW^m	S1–O4	0.085	−0.225	0.078	−0.235	0.007	0.011
QELQEE^m	S1–O2	0.062	−0.237	0.072	−0.249	−0.010	0.012
QELQEE^m	S2–O2	0.073	−0.237	0.074	−0.249	−0.001	0.012
ZAVHEJ^{m*}	S1–O3	0.070	−0.235	0.060	−0.241	0.010	0.006
Se···N							
BESEAZ01^s	Se1–N4	0.250	−0.172	0.258	−0.182	−0.008	0.010
FENFIO^s	Se1–N4	0.109	−0.051	0.112	−0.080	−0.003	0.030
WERYAT^s	Se1–N3	0.131	−0.104	0.153	−0.136	−0.022	0.032
NECZUQ^{m*}	Se3–N1	0.148	−0.142	0.158	−0.159	−0.010	0.017
SECNBZ^m	Se1–N4	0.153	−0.174	0.158	−0.198	−0.006	0.024
Se···O							
BOJCOS^{m*}	Se1–O3	0.086	−0.266	0.080	−0.277	0.006	0.012
LEDGAD^m	Se1–O5	0.199	−0.219	0.192	−0.213	0.007	−0.006
LEDGAD^m	Se3–O1	0.199	−0.214	0.192	−0.213	0.007	−0.002
LEVJOM^m	Se1–O4	0.032	−0.278	0.050	−0.305	−0.019	0.027
MUSCIM^m	Se1–O2	0.071	−0.278	0.072	−0.310	−0.002	0.032

S4 Interaction energies for hydrogen bonds, halogen bonds and chalcogen bonds

In this section we present the values of interaction energies in kcal/mol for the hydrogen bonds (Table S6), halogen bonds (Table S7), and chalcogen bonds (Table S8). Interaction energies were calculated at M062x/Def2TZVP level. The atomic and molecular Roby-Gould bond indices are also given.

Table S6: Interaction energy (kcal/mol) for hydrogen bonds (D−H···A, D= C, N, O, A= N, O). Interaction energies were calculated at M062x/Def2TZVP level. The single and multipliable contact interactions in dimers are marked with superscript s and m respectively (and $m*$ for those that have two identical interactions within one dimer).

D−H···A<br>CSD code	Interaction energy<br>(kcal/mol)
C−H···N	
CYACHZ01^{s}	3.15
DMSDIM01^{s}	2.03
MEADEN02^{s}	1.96
TRAZOL02^{s}	2.76
BUGKIX01N^{m}	2.00
BUGKIX01^{m}	5.13
CYACHZ01^{m}	1.64
NURWOM02^{m*}	3.67
NURWOM02^{m}	3.04
TEPNIT04^{m*}	5.94
XEHMOM01^{m}	2.06
XEHMOM01^{m}	2.26
N−H···N	
BAZGOY05^{s}	2.03
DMSDIM01^{s}	4.91
FORAMO01^{s}	4.15
IMAZOL06^{s}	9.19
TRAZOL02^{s}	8.41
O−H···N	
FORAMO01^{s}	7.35
GLOXIM11^{s}	7.18
MAMPOL02^{s}	7.53
LOLSUA^{m}	18.50
ULAWAF05^{m}	27.45
C−H···O	
ABINOR04^{s}	2.12
NBONAN01^{s}	1.12
POKKAD01^{s}	1.83
CYACHZ01^{m*}	13.37
FACETA01^{m*}	5.27
GLYGLY04^{m}	16.86
METURA01^{m}	4.96
N−H···O	
BZAMID02^{m*}	15.86
CYURAC12^{m*}	14.02
FACETA01^{m*}	16.38
ULAWAF05^{m*}	16.28
UROXAL01^{m*}	18.79
ZECWUB01^{m*}	27.38
O−H···O	
BESKAL10^{m*}	18.65
CUKCAM18^{m*}	22.07
SALIAC12^{m*}	19.02
SUCACB03^{m*}	20.28
ZZZEEU05^{m*}	20.48

Table S7: Interaction energy (kcal/mol) for halogen bonds (X···A, X= Cl, Br, A= N, O). Interaction energies were calculated at M062x/Def2TZVP level. The single and multipliable contact interactions in dimers are marked with superscript s and m respectively (and $m*$ for those that have two identical interactions within one dimer).

X···A CSD code	Interaction energy (kcal/mol)
Cl···N	
CCACENN^s	2.99
DESKER01^s	0.91
NABZAS^s	2.44
PCLPYR^s	2.65
VUGSIZ^s	1.79
PALPAV^m	2.44
XIZPON^m	1.35
Cl···O	
BEDMONN^s	1.46
BZQDCL11^s	1.53
CORDUI^s	0.64
DCLBZQ20^s	1.48
IRUFEH01^s	1.56
JOJTIL^s	2.30
RUBSUD^s	1.56
TCACAD01^s	1.28
GEXWUB^s	1.74
PEPFUL^s	1.39
Br···N	
BCACENN^s	3.88
BONFIT^s	3.12
QONHUX^s	2.36
RIRFOON^s	1.71
KUYCUD^s	2.83
Br···O	
BMLTAAN^s	2.18
CIRSONN^s	0.98
JEVVOW^s	3.00
VAQXUG^s	2.11
VEWTAU^s	2.49
VEWTEY^s	1.46
VITVEZ^s	2.50
WADFIR^s	1.96
ACETBR02^m	5.59

Table S8: Interaction energy (kcal/mol) for chalcogen bonds (Y ⋯ A, Y= S, Se, A= N, O). Interaction energies were calculated at M062x/Def2TZVP level. The single and multipliable contact interactions in dimers are marked with superscript *s* and *m* respectively (and *m*∗ for those that have two identical interactions within one dimer).

Y ⋯ A<br>CSD code	Interaction energy<br>(kcal/mol)
S ⋯ N	
CEBYUD^{s}	2.29
QOBFUI^{s}	2.65
SAZCEC^{s}	2.23
GEDHAY^{m}	6.18
IFULUQ04^{m}	23.94
WASHEE^{m}	23.73
WUXPAG^{m}	33.27
S ⋯ O	
PAFVEY^{s}	2.93
WOCQEK^{s}	6.01
ADOFEF^{m*}	5.05
MAVRAD^{m}	7.57
MEHNIY^{m}	35.52
NAHMUE^{m}	3.61
PUDMUW^{m}	2.97
QELQEE^{m}	4.79
ZAVHEJ^{m*}	4.30
Se ⋯ N	
BESEAZ01^{s}	3.06
FENFION^{s}	3.87
WERYAT^{s}	4.53
NECZUQ^{m*}	8.75
SECNBZ^{m}	6.61
Se ⋯ O	
BOJCOS^{m*}	4.88
LEDGAD^{m}	6.11
LEVJOM^{m}	4.57
MUSCIM^{m}	5.67

S5 Testing the conservation of Roby-Gould bond bond order

Table S9: Total (D−X) and (A⋯X) atom⋯atom Roby-Gould bond indices (τ) for 15 selected dimers and corresponding monomers (τ(D−X) and τ(A⋯X) respectively). The sum of the D−X and A⋯X bond indices in dimers ($\tau(\mathrm{D{-}A} + \mathrm{A}\cdots\mathrm{X})_{Dimer}$) and the differences in D−X bond index between dimers and monomers ($\tau(\Delta_{\mathrm{D-X}})$) are also given. Wavefunctions optimized at the M062x/Def2SVP level.

CSD code	Interaction	$\tau(\mathrm{D{-}X})_{Monomer}$	$\tau(\mathrm{D{-}X})_{Dimer}$	$\tau(\mathrm{A}\cdots\mathrm{X})_{Dimer}$	$\tau(\mathrm{D{-}A} + \mathrm{A}\cdots\mathrm{X})_{Dimer}$	$\tau(\Delta_{\mathrm{D-X}})$
IMAZOL06	N−H⋯N	0.942	0.814	0.272	1.086	0.128
FORAMO01	O−H⋯N	0.945	0.784	0.338	1.121	0.162
ULAWAF05	O−H⋯N	0.930	0.694	0.385	1.079	0.236
METURA01	C−H⋯O	0.934	0.914	0.167	1.081	0.020
METURA01	C−H⋯O	0.922	0.891	0.174	1.065	0.031
BZAMID02	N−H⋯O	0.946	0.831	0.283	1.114	0.114
SUCACB03	O−H⋯O	0.927	0.738	0.379	1.117	0.190
DESKER01	Cl⋯N	1.210	1.200	0.060	1.260	0.010
BCACEN	Br⋯N	1.466	1.428	0.139	1.568	0.037
RIRFOO	Br⋯N	1.150	1.130	0.140	1.270	0.020
ACETBR02	Br⋯O	0.739	0.695	0.170	0.865	0.044
CIRSON	Br⋯O	1.140	1.130	0.120	1.250	0.010
JEVVOW	Br⋯O	1.120	1.100	0.150	1.250	0.020
ZAVHEJ	S⋯O	0.922	0.912	0.186	1.098	0.010
BOJCOS	Se⋯O	0.946	0.926	0.221	1.146	0.020

S6 The angle dependence of RGBI values

Two angles are most important in the discussion of the angle dependence of bond orders of a D−X···A intermolecular interaction, (i) ∠D−X···A angle (ii) ∠X···A−C angle. Among these, ∠D−X···A angle is more significant as a nearly 180 ∠D−X···A angle is directly linked to the effective $n \rightarrow \sigma^*$ interaction. To test these we calculated and plotted the bond indices, total, covalent and ionic bond indices along with the AIM topological parameters at the bond critical points for a selected subset of interactions with N atom as the lone pair donor or bond acceptor (Table S10 and Figure S1). We find that these results are insufficient to show the directional sensitivity of RGBIs as compared to AIM topology. We have plotted total, covalent and ionic RGBIs for a subset of halogen and chalcogen bonded complexes versus ∠D−X···A angle (Figure S1). No clear correlation could be found between these descriptors and the ∠D−X···A angle. It may also be noted that more complicated interaction angles around atom A and its hybridization state (as it is related to lone pair directionality) can also affect bond orders. The wide range of bond order values for interaction types such as XBs and YBs may be due to a variety of reasons and the bond angle directionality is only one of them. Chemical environment around the X and A atoms involved in the interaction, the partial charges of atoms A and X (even in monomer state) etc can influence the bond order values. However, we performed similar analysis on the hypothetical geometries for molecular dimers varying the interaction angles for the linear structures N≡C—C≡C—Br and N≡C—C≡C—Cl (CSD codes: BCACEN, and CCACEN respectively) which exhibit Br···N and Cl···N interactions. This analysis clearly brings out the differences in angle dependent trends in RGBIs vs AIM topological parameters.

Table S10: The angle dependence of total (τ), covalent (c), and ionic (i) Roby-Gould bond indices, and AIM topological parameters of a D−X···A intermolecular interaction with respect to D−X···A angle for a selected set of halogen and chalcogen bonded dimers. The distance (in Å) and EML interaction energy (in kJ/mol) are also given.

A···X CSD code	$d_{A\cdots X}$ /Å	Angle ($\theta°$) $\angle D-X\cdots A$	ρ /bcp	$\nabla^2\rho$	EML (kJ/mol) interaction energy	c	i	τ
Cl···A								
CCACEN^s	2.984	178.250	0.074	1.148	−9.243	0.060	0.440	0.450
DESKER01^s	2.954	163.710	0.091	1.275	−11.580	0.070	0.350	0.360
NABZAS^s	3.092	170.100	0.066	0.931	−7.627	0.040	0.090	0.090
PCLPYR^s	3.014	180.000						
VUGSIZ^s	3.100	172.910	0.065	0.928	−7.455	0.040	0.100	0.110
PALPAV^m	3.097	149.620	0.063	0.936	−7.395	0.040	0.000	0.040
Br···N								
BCACEN^s	2.978	177.230	0.090	1.301	−11.591	0.090	0.400	0.410
BONFIT^s	2.863	171.250	0.119	1.593	−16.264	0.130	0.250	0.280
QONHUX^s	3.093	153.630	0.080	1.026	−9.348	0.070	0.240	0.250
RIRFOO^s	3.164	167.460	0.072	0.920	−8.063	0.060	0.260	0.260
S···N								
CEBYUD^s	3.050	149.490	0.079	0.998	−9.037	0.080	0.070	0.110
QOBFUI^s	2.992	170.010	0.088	1.101	−10.443	0.080	0.030	0.090
SAZCEC^s	3.096	171.920	0.073	0.917	−8.126	0.080	0.050	0.090
GEDHAY^m	2.910	169.730	0.095	1.270	−11.998	0.110	0.020	0.110
GEDHAY^m	3.086	153.830	0.075	1.011	−8.762	0.080	0.060	0.100
IFULUQ04^m	3.006	159.870	0.091	1.230	−11.383	0.060	0.110	0.130
WASHEE^m	3.003	161.620	0.089	1.205	−11.022	0.270	0.130	0.300
WASHEE^m	2.992	161.140	0.093	1.184	−11.296	0.060	0.110	0.120
WUXPAG^m	3.008	161.480	0.082	1.141	−10.003	0.100	0.000	0.100
Se···N								
BESEAZ01^s	3.155	169.160	0.075	0.857	−8.015	0.100	0.21	0.230
FENFIO^s	3.154	175.810	0.082	0.890	−8.887	0.100	0.01	0.100
WERYAT^s	2.843	173.990	0.139	1.543	−18.647	0.150	0.000	0.150
NECZUQ^{m*}	2.877	168.200	0.124	1.379	−15.953	0.180	0.150	0.230
SECNBZ^m	3.058	174.740	0.083	1.050	−9.718	0.100	0.140	0.170

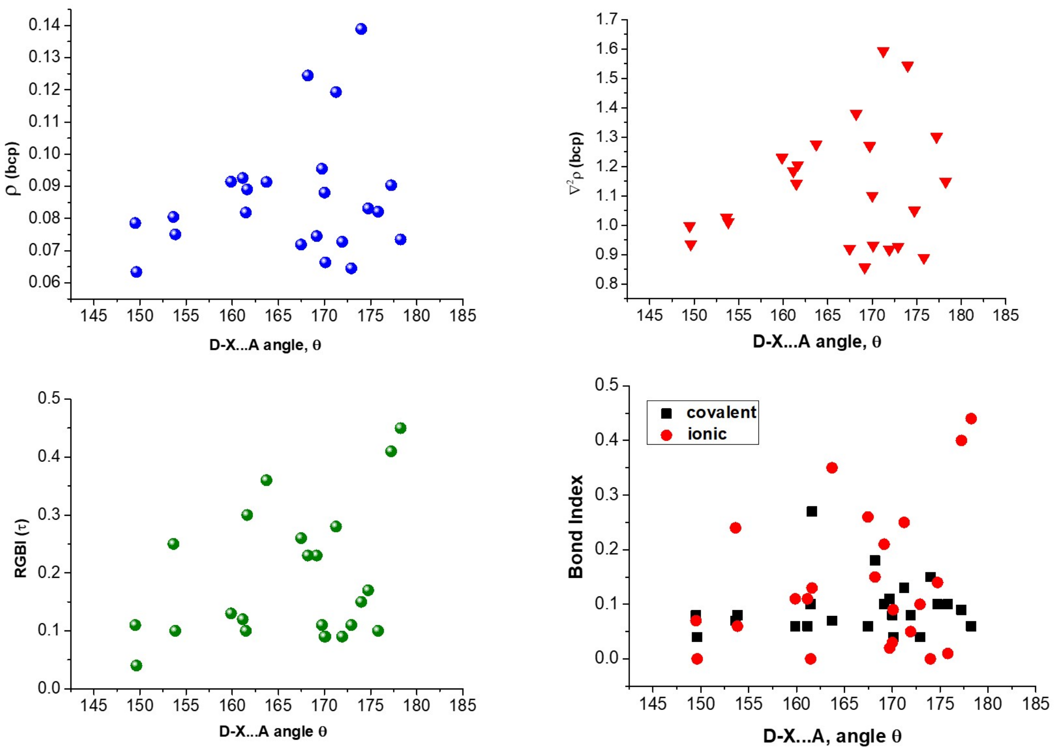

Figure S1: The angle dependence of total (τ), covalent (c), and ionic (i) Roby-Gould bond indices and AIM topological parameters of a D$-$X$\cdots$A intermolecular interaction with respect to D$-$X$\cdots$A angle for a selected set of halogen and chalcogen bonded dimers given in the Table S10.

S7 Percentage of covalency

Figure S2 shows bond order-weighted average covalency percentages ($\%c_W$) for atom$\cdots$atom (light blue) and molecul$\cdots$molecule bond indices (dark blue) for hydrogen, halogen and chalcogen bonds. For each interaction type, the average weighted covalency percentages were calculate using the formula:

$$\%c_W = \frac{\sum \tau_i.\%c_i}{\sum \tau_i},$$

where τ_i and $\%c_i$ are the bond orders and percentage covalency of each interaction under XB, YB and HB.

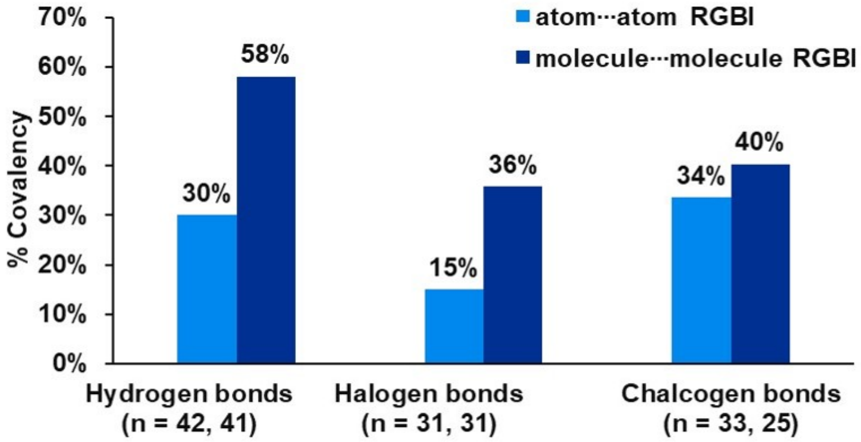

Figure S2: Bond order-weighted average covalency percentages ($\%c_W$) for atom$\cdots$atom (light blue) and molecul$\cdots$molecule bond indices (dark blue) for hydrogen, halogen and chalcogen bonds. Number of interactions and molecular dimers studied for each type are given in parentheses.

S8 Molecular structures of selected dimers

In this section we present the molecular structures of all dimers considered in this study with the hydrogen bonds, halogen bonds and chalcogen bonds. The distances in Angstrom are given for only those interactions concerned in this study.

These figures were generated from the crystallographic information files (CIF) in the Cambridge Crystallographic Structure Database (CSD) using CrystalExplorer program.

Please note that the molecular structures of all dimers are listed according to the same order where their CSD codes are listed in the Tables 2, 3 and S1.

The atoms of each dimer are labeled with different colored spheres. as follows:

- Hydrogen atom: yellow (small ball).
- Carbon atom: dark gray.
- Oxygen atom: red.
- Nitrogen atom: blue.
- chlorine atom: green.
- Bromine atom: gold.
- Sulfur atom: yellow (big ball).
- Selenium atom: light gray (big ball).

S8.1 Hydrogen bonds

S8.1.1 C–H···N interaction

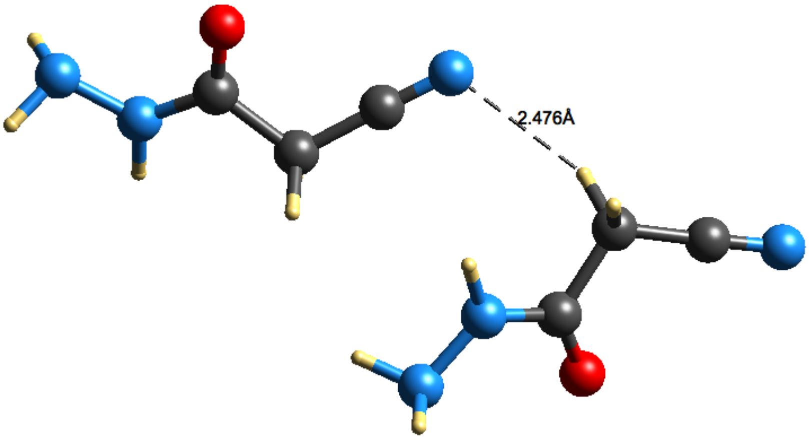

Figure S3: The molecular structure of CYACHZ01^{s}.

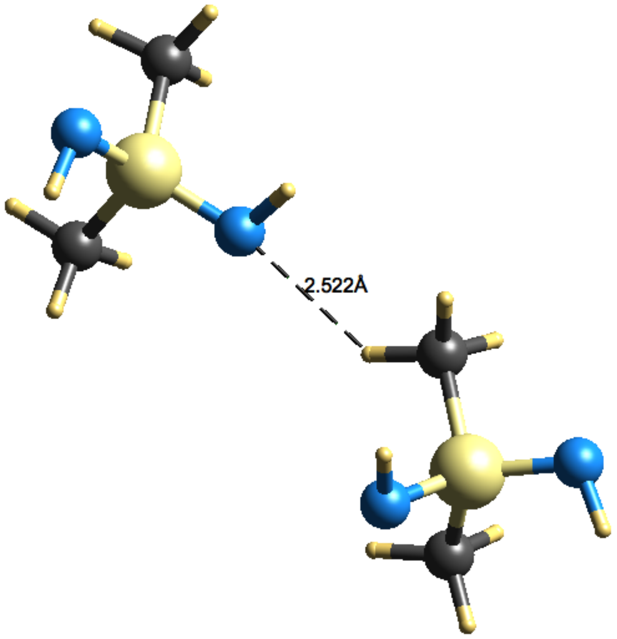

Figure S4: The molecular structure of DMSDIM01^{s}.

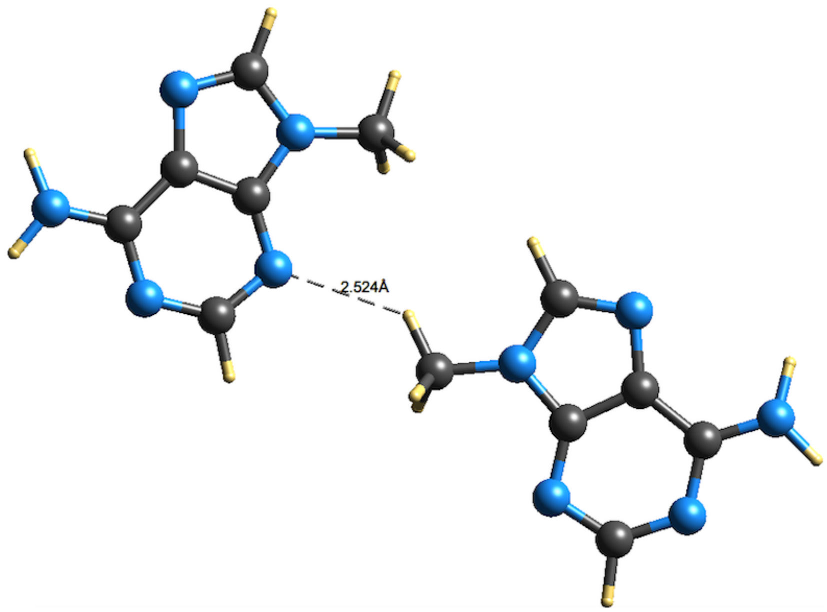

Figure S5: The molecular structure of MEADEN02^s.

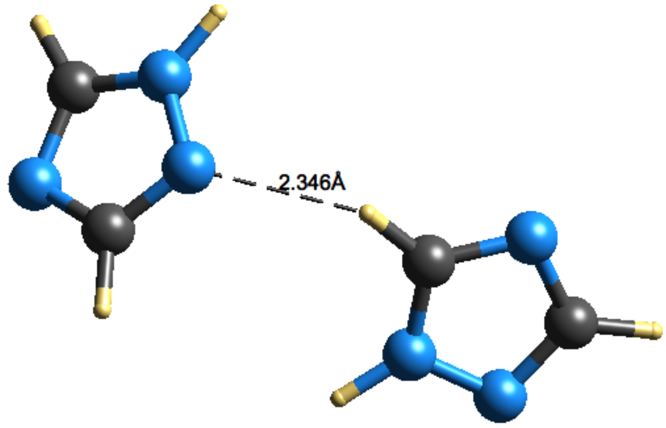

Figure S6: The molecular structure of TRAZOL02^s.

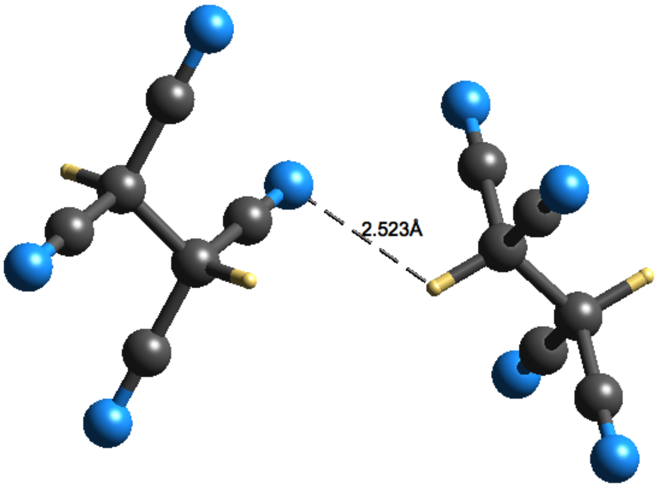

Figure S7: The molecular structure of BUGKIX01^{s}.

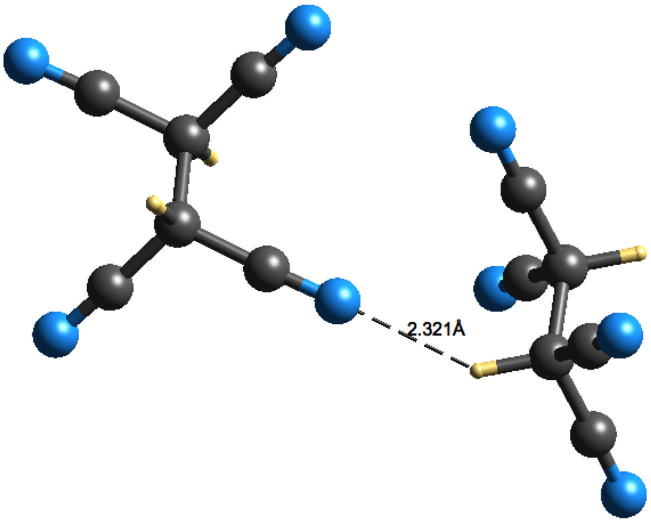

Figure S8: The molecular structure of BUGKIX01^{s}.

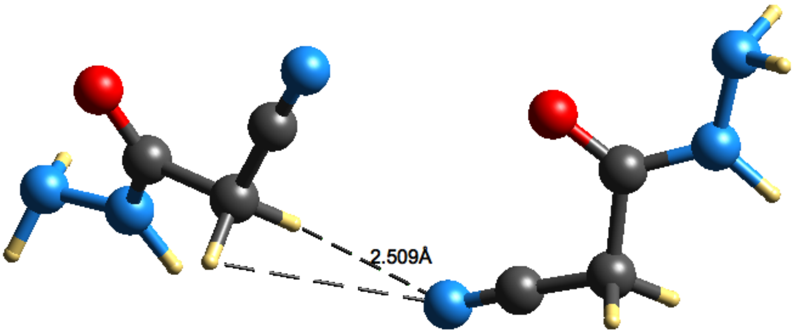

Figure S9: The molecular structure of CYACHZ01^{m}.

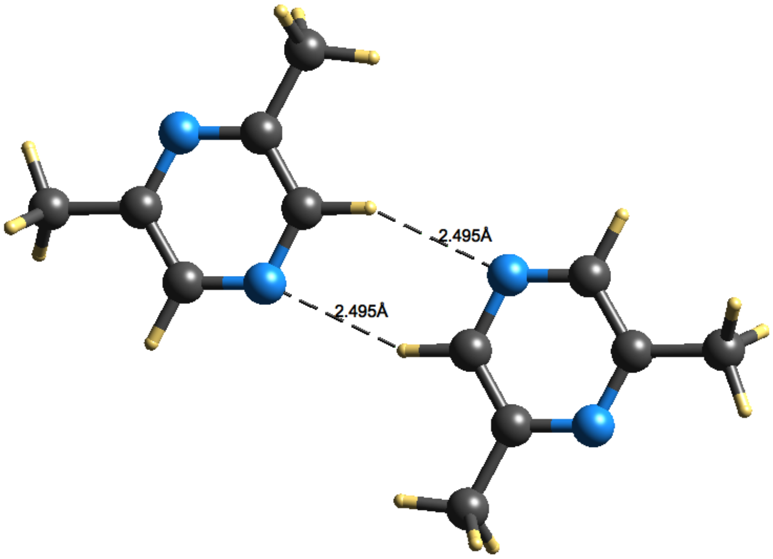

Figure S10: The molecular structure of NURWOM02^{m*}.

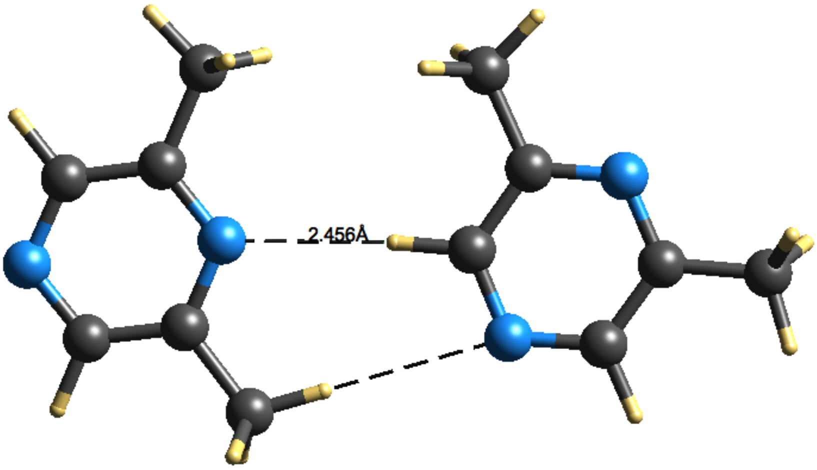

Figure S11: The molecular structure of NURWOM02^{m}.

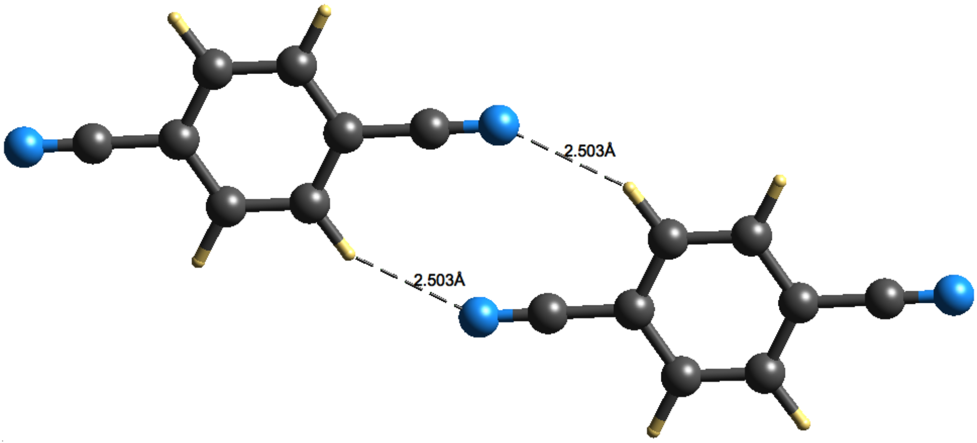

Figure S12: The molecular structure of TEPNIT04^{m*}.

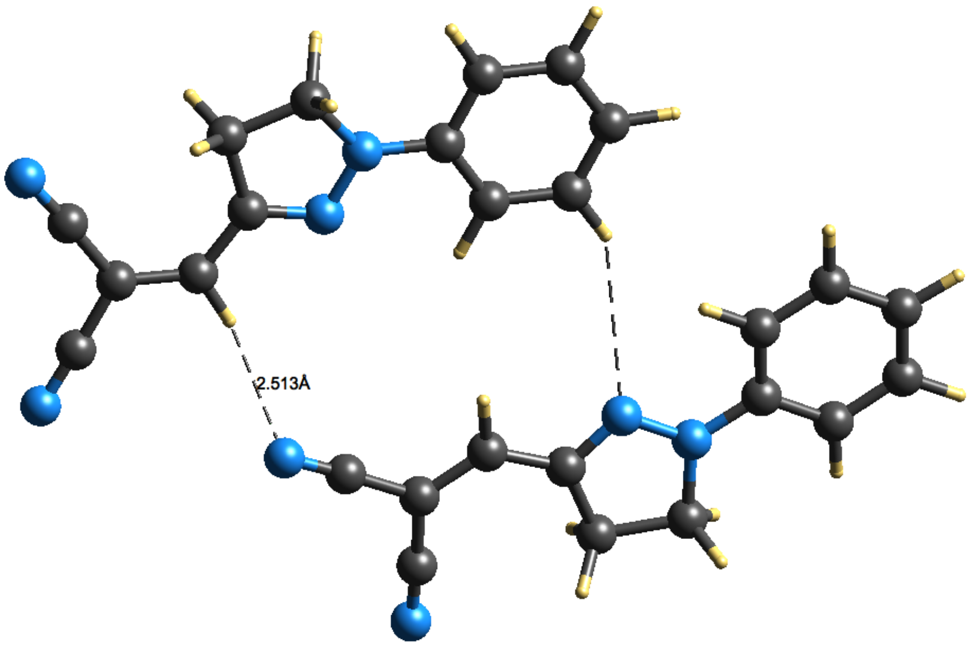

Figure S13: The molecular structure of XEHMOM01^{m}.

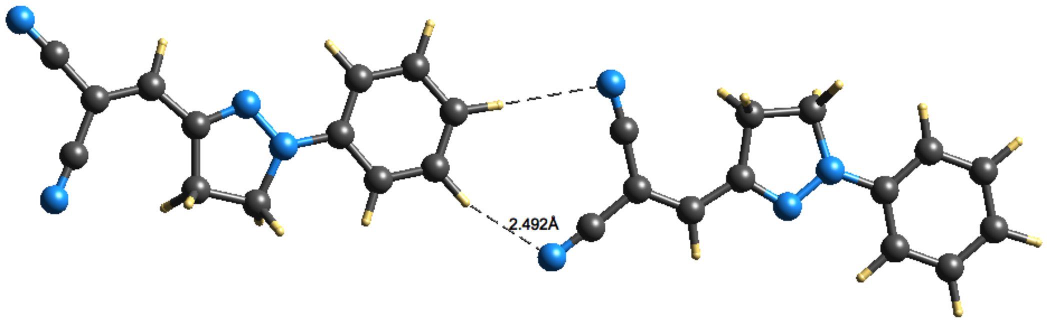

Figure S14: The molecular structure of XEHMOM01^{m}.

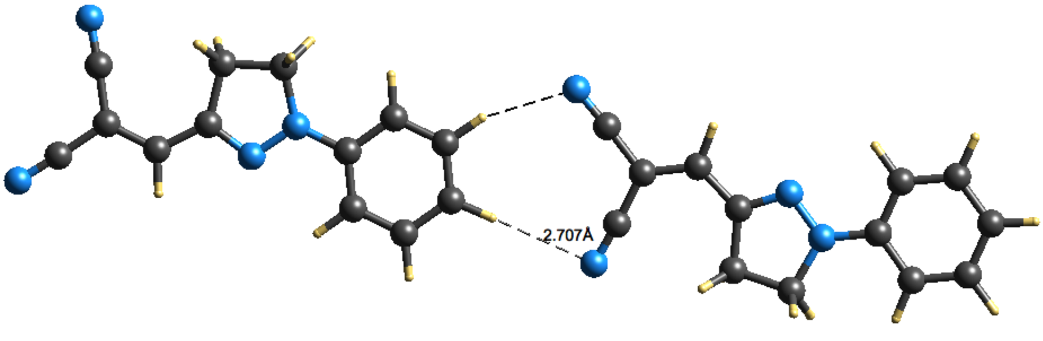

Figure S15: The molecular structure of XEHMOM01^{m}.

S8.1.2 N–H···N interactions

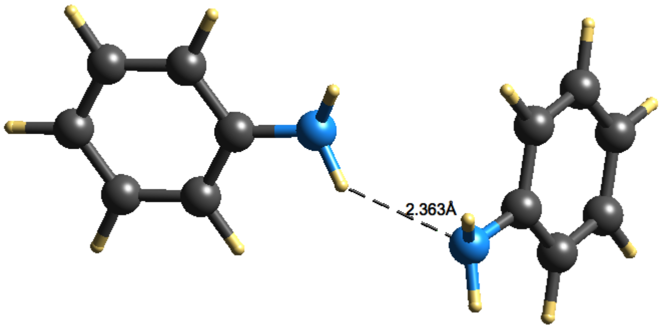

Figure S16: The molecular structure of BAZGOY05[s].

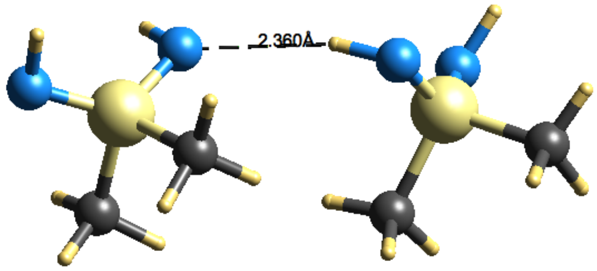

Figure S17: The molecular structure of DMSDIM01[s].

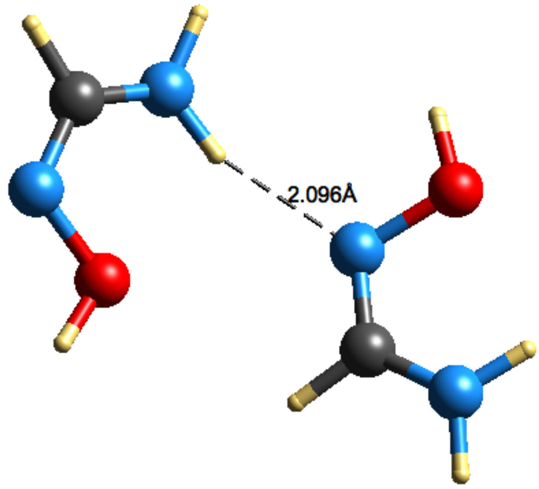

Figure S18: The molecular structure of FORAMO01[s].

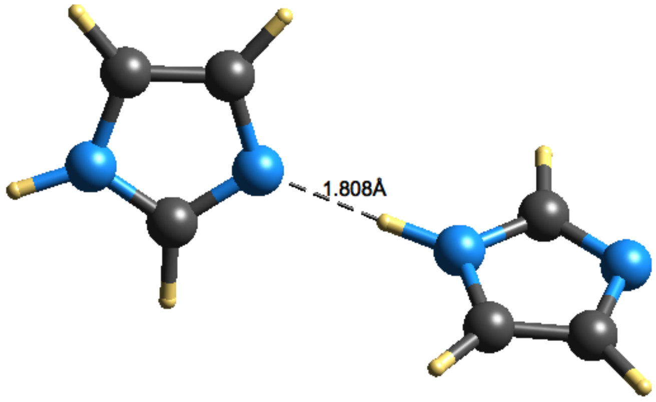

Figure S19: The molecular structure of IMAZOL06^s.

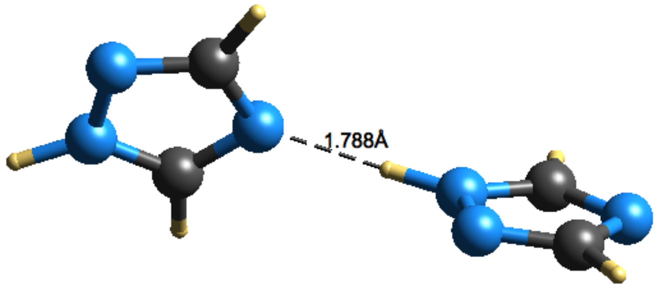

Figure S20: The molecular structure of TRAZOL02^s.

S8.1.3 O–H···N interactions

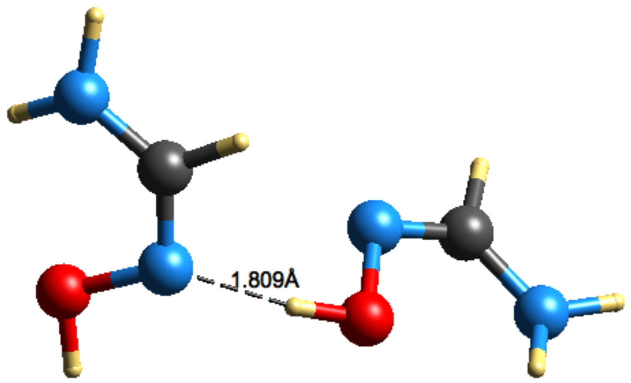

Figure S21: The molecular structure of FORAMO01^s.

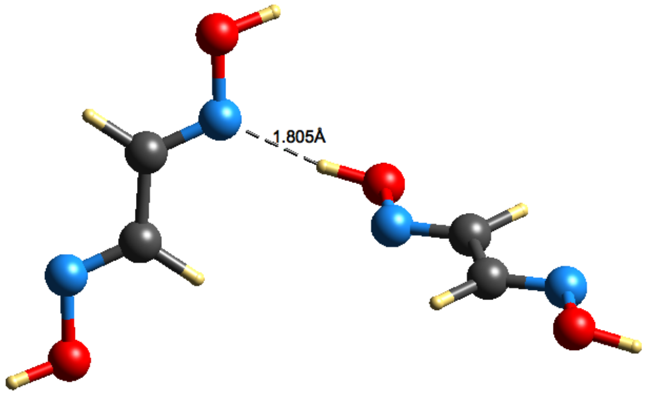

Figure S22: The molecular structure of GLOXIM11^s.

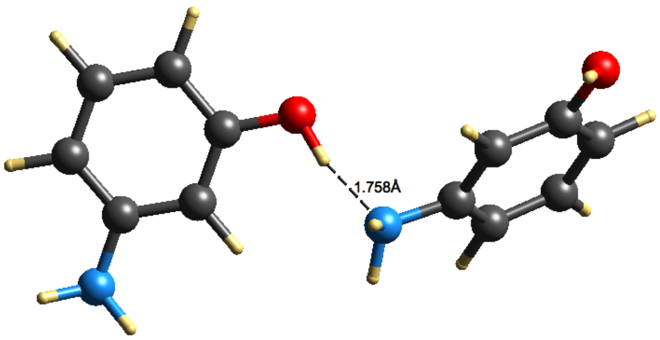

Figure S23: The molecular structure of MAMPOL02^s.

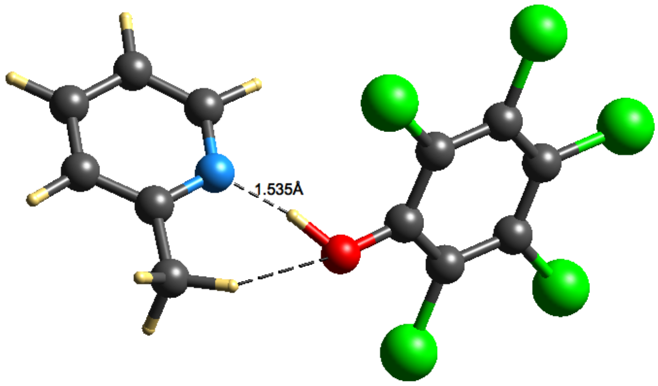

Figure S24: The molecular structure of LOLSUA^{m}.

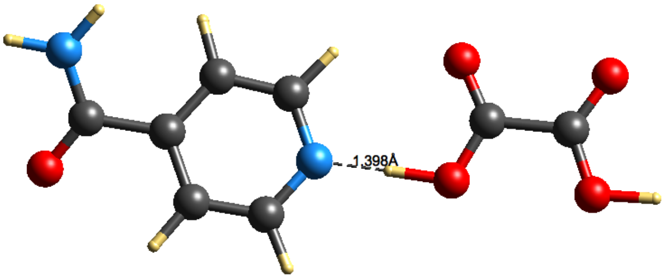

Figure S25: The molecular structure of ULAWAF05^{m}.

S8.1.4 C–H···O interactions

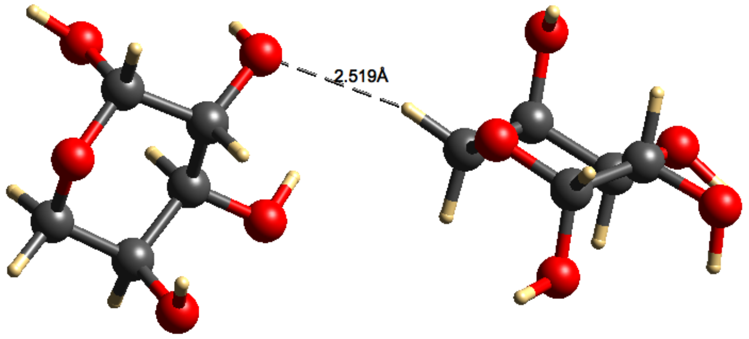

Figure S26: The molecular structure of ABINOR04^s.

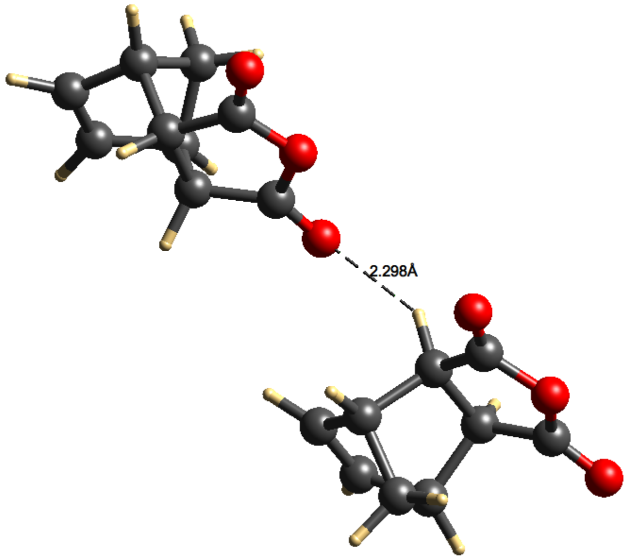

Figure S27: The molecular structure of NBONAN01^s.

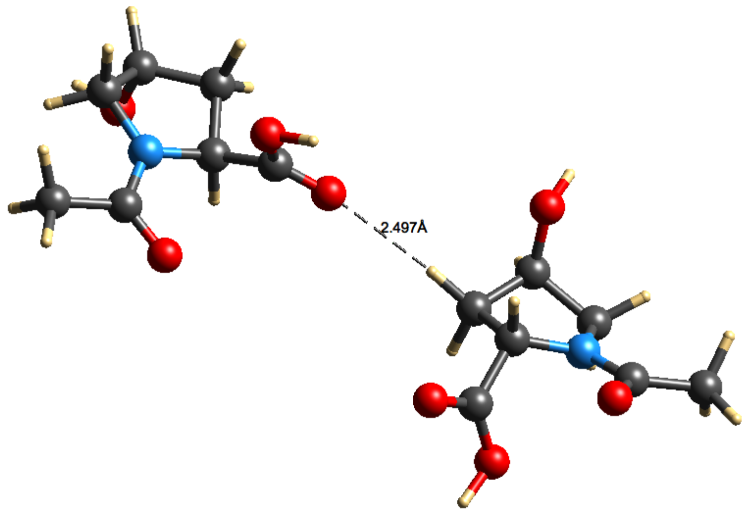

Figure S28: The molecular structure of POKKAD01^s.

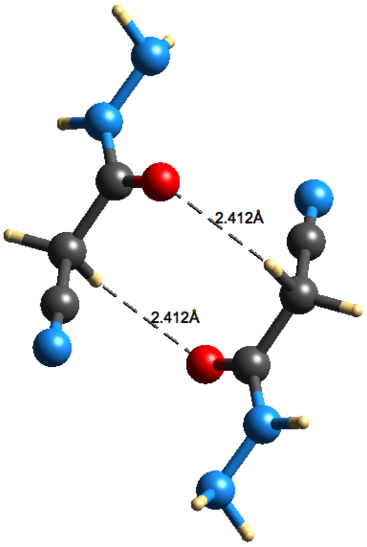

Figure S29: The molecular structure of CYACHZ01^{m*}.

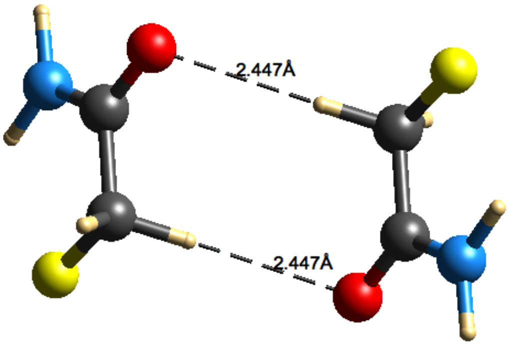

Figure S30: The molecular structure of FACETA01^{m*}.

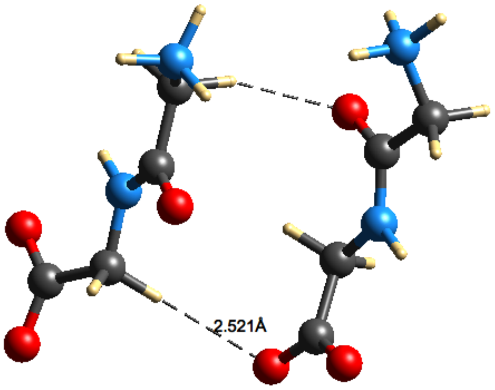

Figure S31: The molecular structure of GLYGLY04^{m}.

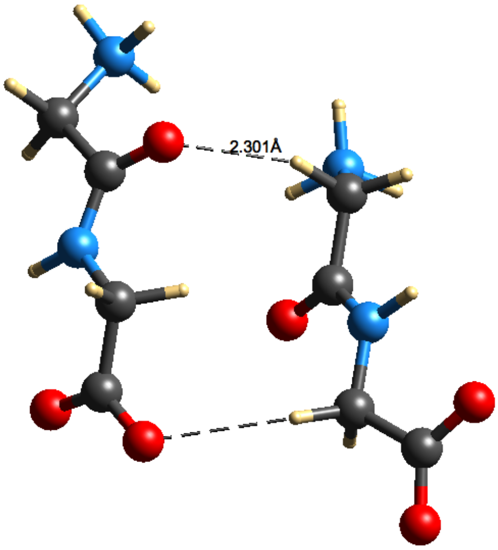

Figure S32: The molecular structure of GLYGLY04^{m}.

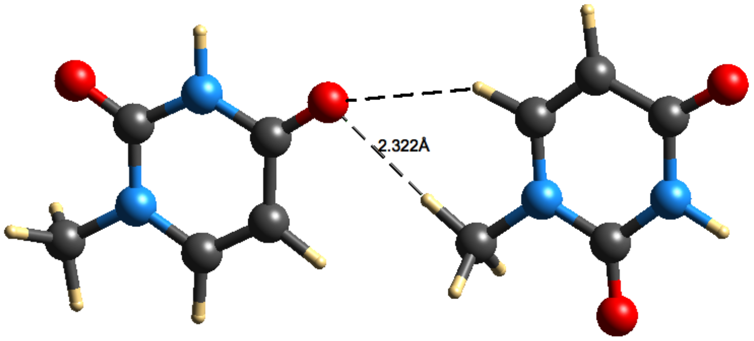

Figure S33: The molecular structure of METURA01^{m}.

S8.1.5 N–H···O interactions

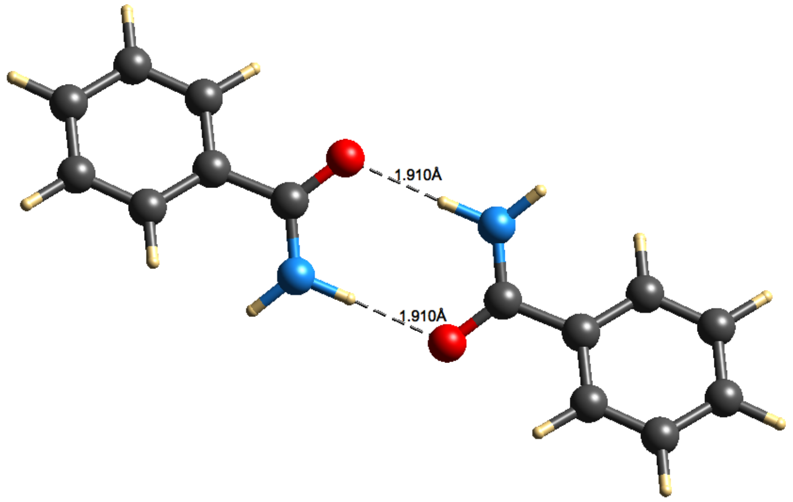

Figure S34: The molecular structure of BZAMID02^{m*}.

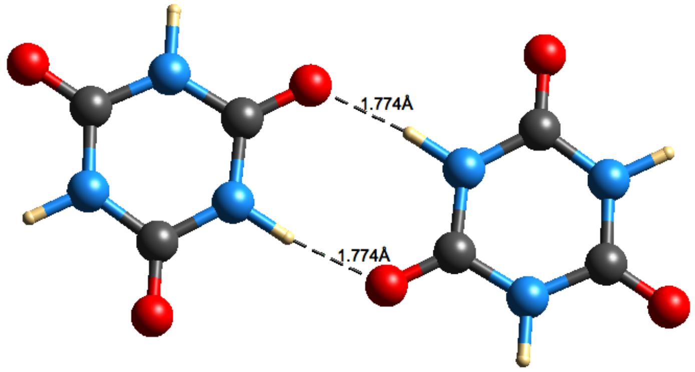

Figure S35: The molecular structure of CYURAC12^{m*}.

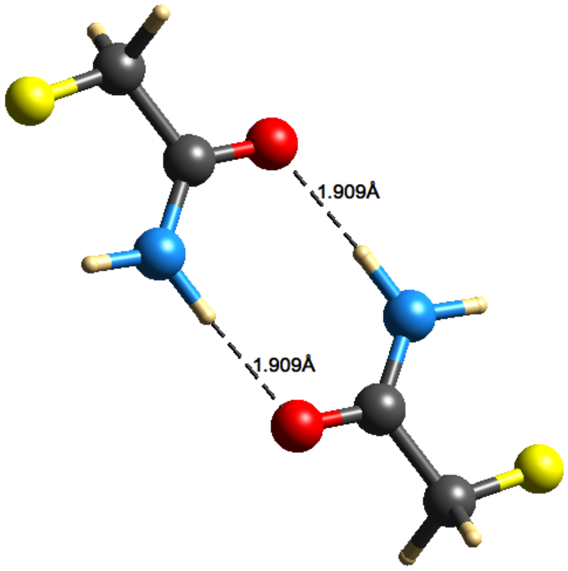

Figure S36: The molecular structure of FACETA01^{m*}.

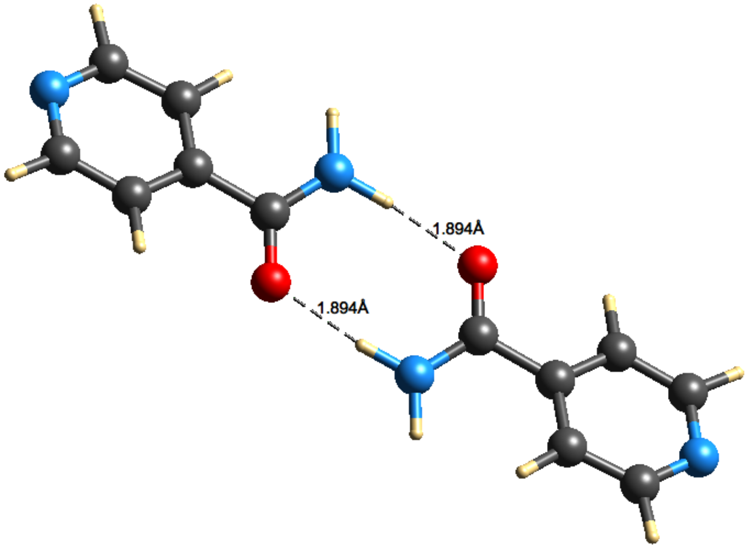

Figure S37: The molecular structure of ULAWAF05^{m*}.

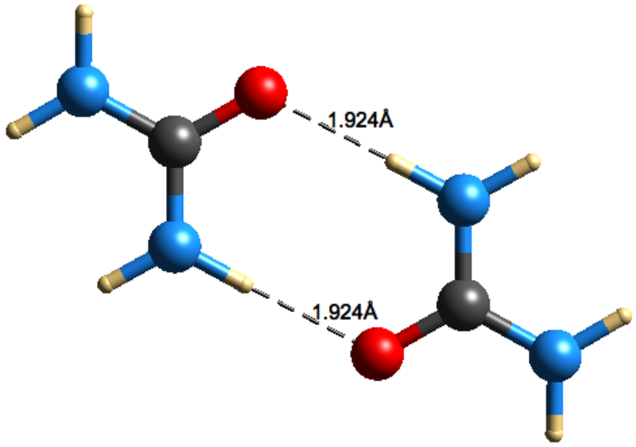

Figure S38: The molecular structure of UROXAL01^{m*}.

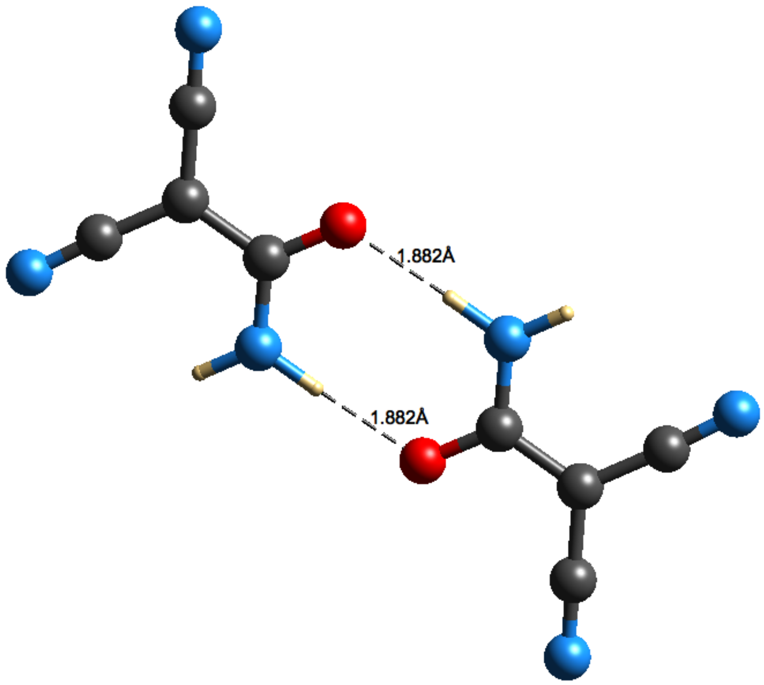

Figure S39: The molecular structure of ZECWUB01^{m*}.

S8.1.6 O–H···O interactions

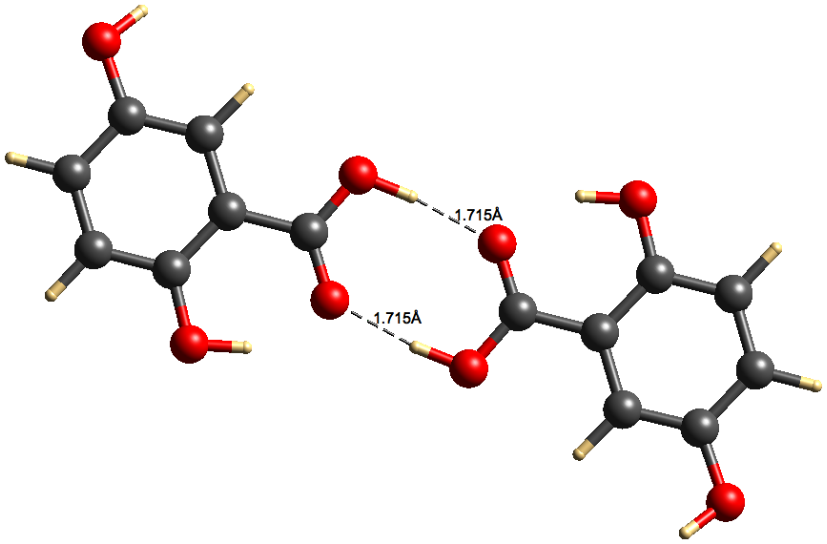

Figure S40: The molecular structure of BESKAL10^{m*}.

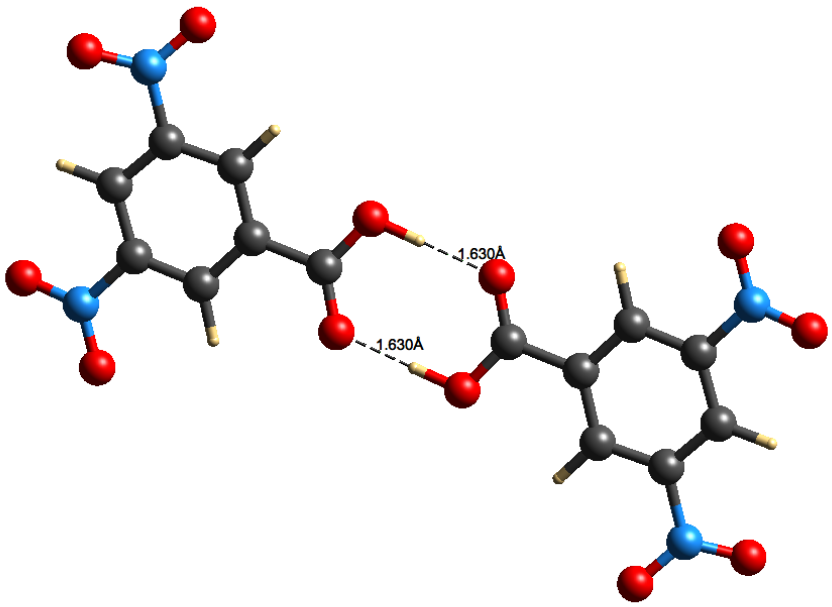

Figure S41: The molecular structure of CUKCAM18^{m*}.

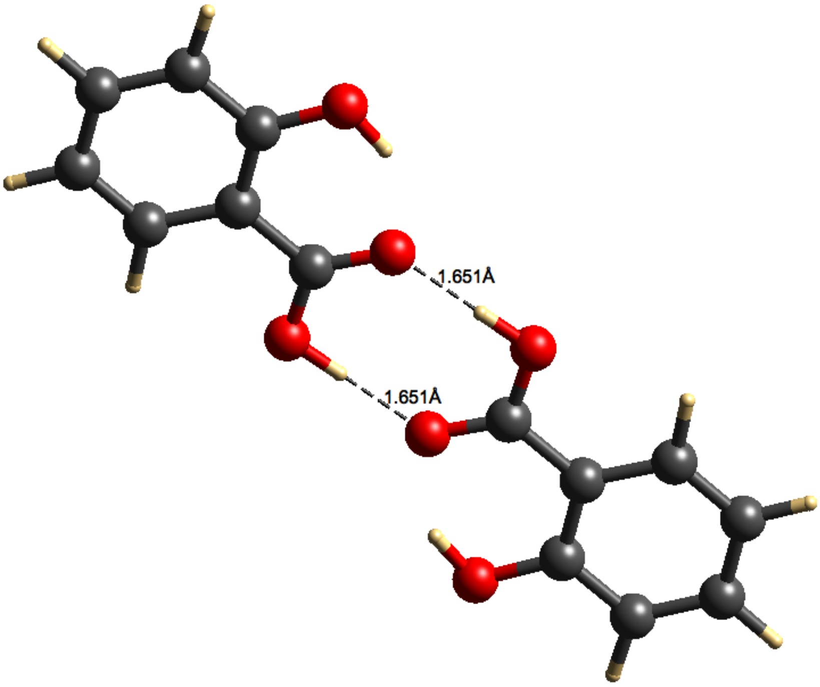

Figure S42: The molecular structure of SALIAC12^{m*}.

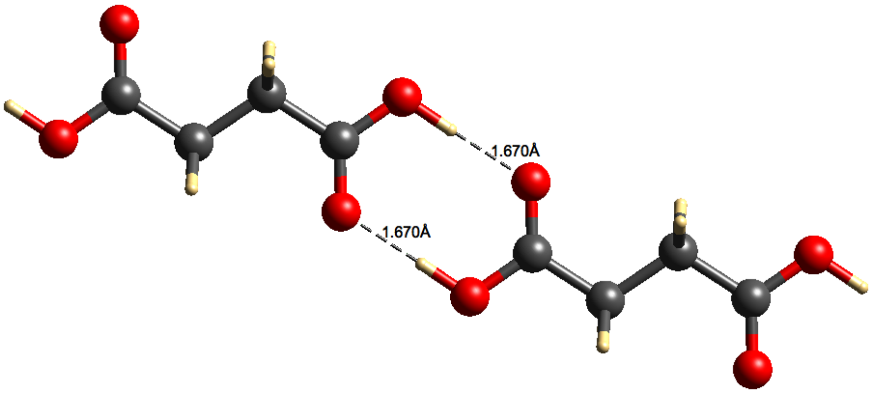

Figure S43: The molecular structure of SUCACB03^{m*}.

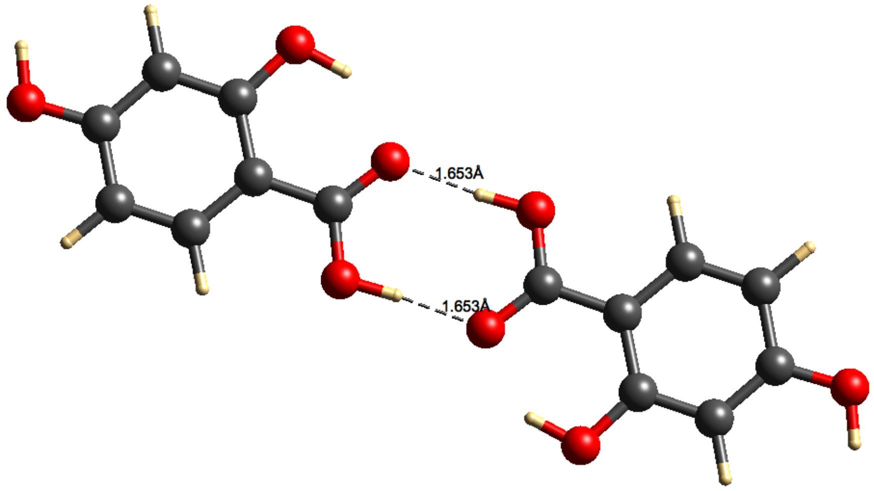

Figure S44: The molecular structure of ZZZEEU05^{m*}.

S8.2 Halogen bonds

S8.2.1 Cl$\cdots$N interactions

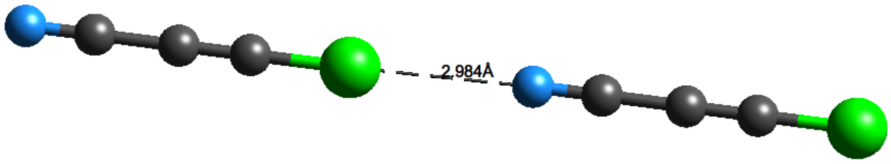

Figure S45: The molecular structure of CCACENN^{s}.

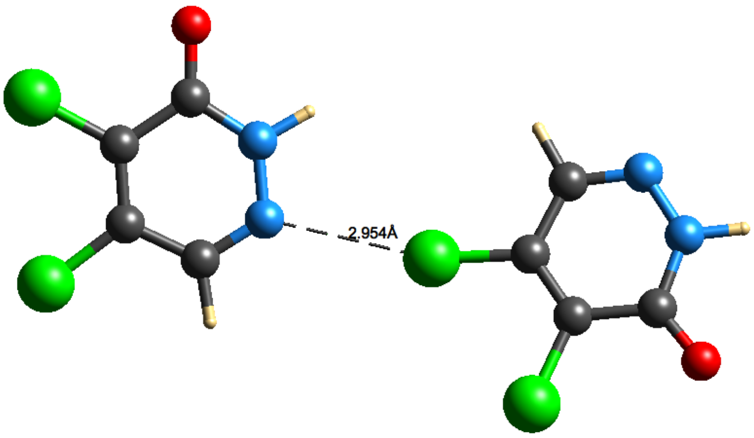

Figure S46: The molecular structure of DESKER01^{s}.

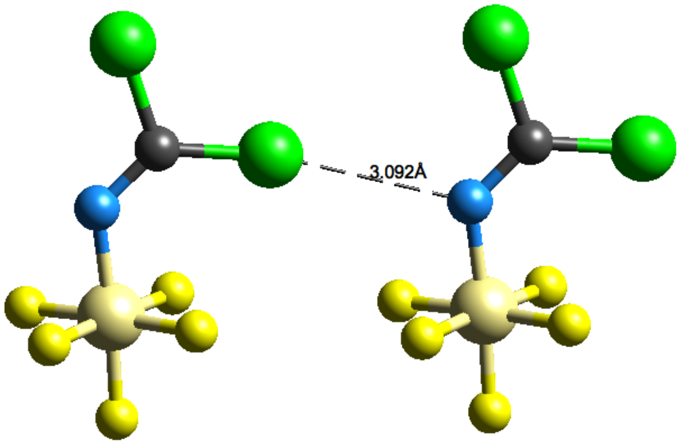

Figure S47: The molecular structure of NABZAS^{s}.

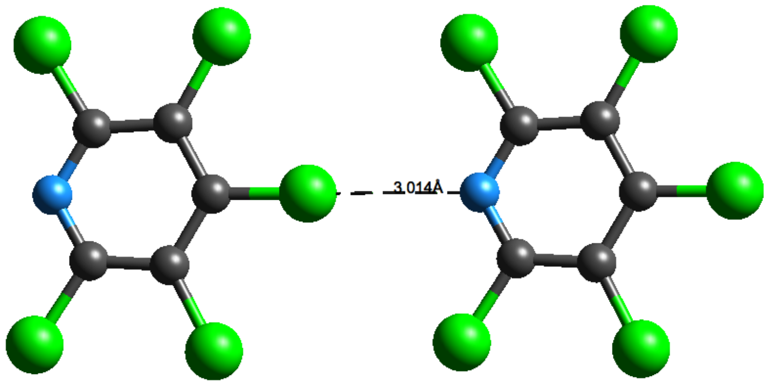

Figure S48: The molecular structure of PCLPYR^s.

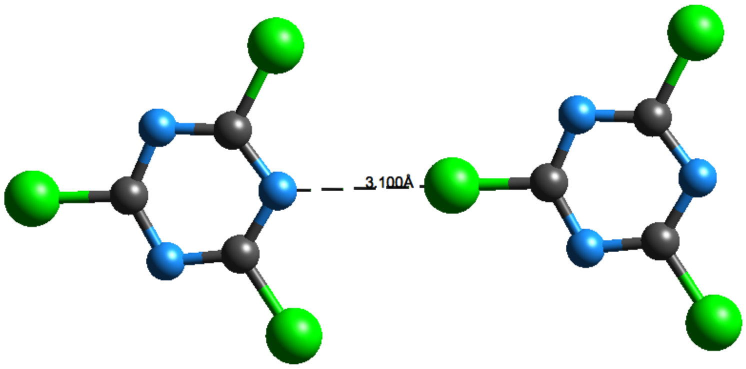

Figure S49: The molecular structure of VUGSIZ^s.

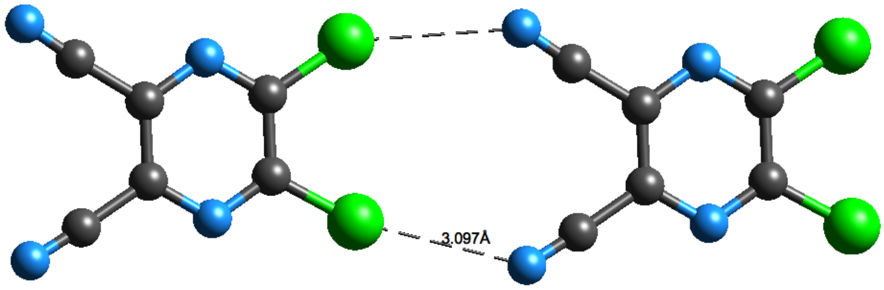

Figure S50: The molecular structure of PALPAV^m.

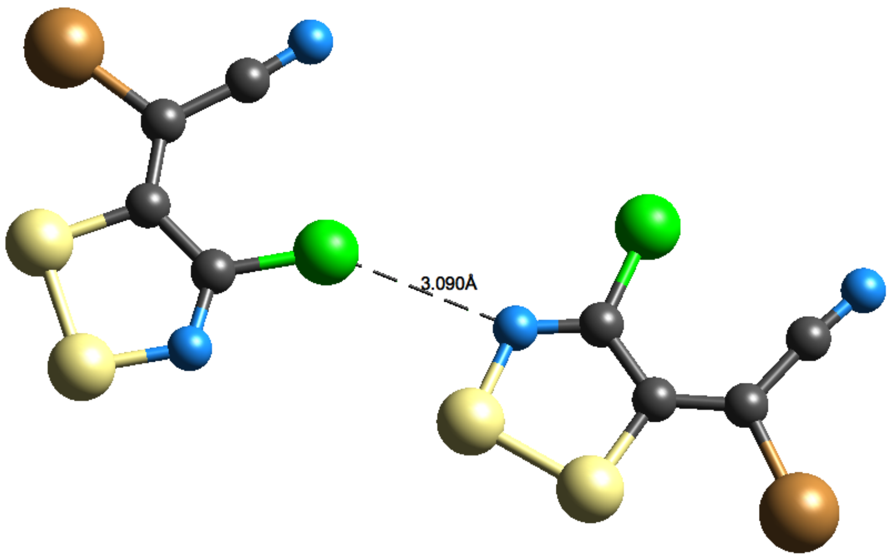

Figure S51: The molecular structure of XIZPON^{s}.

S8.2.2 Cl···O interactions

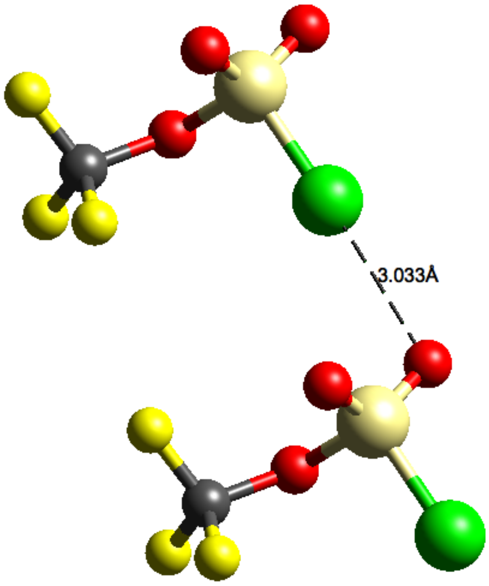

Figure S52: The molecular structure of BEDMON^s.

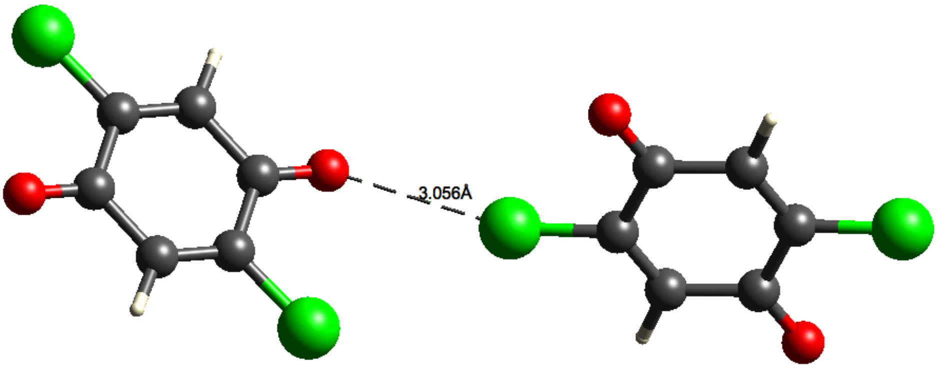

Figure S53: The molecular structure of BZQDCL11^s.

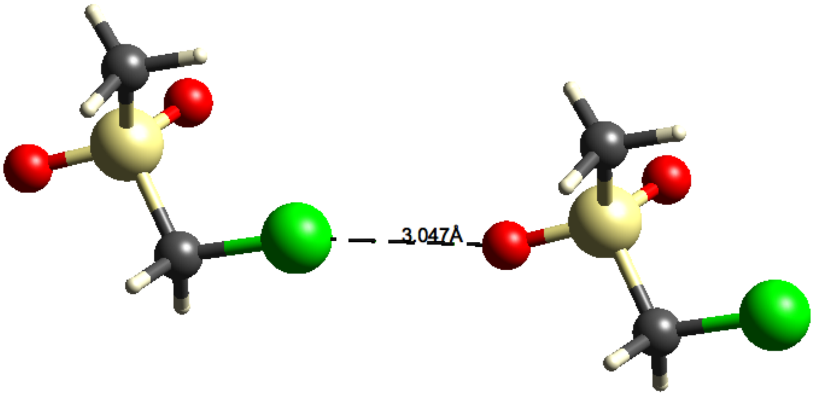

Figure S54: The molecular structure of CORDUI^s.

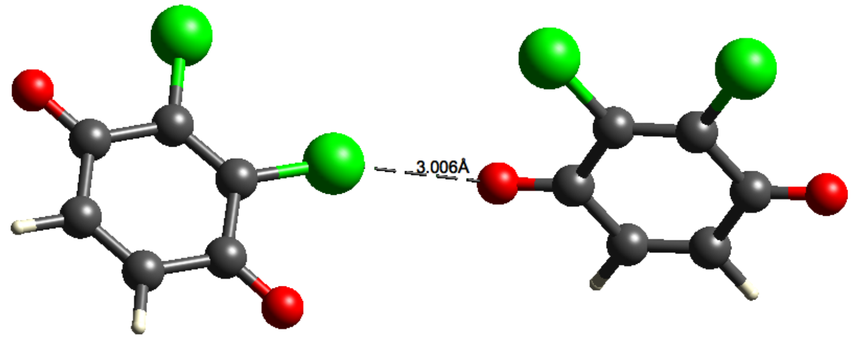

Figure S55: The molecular structure of DCLBZQ20^s.

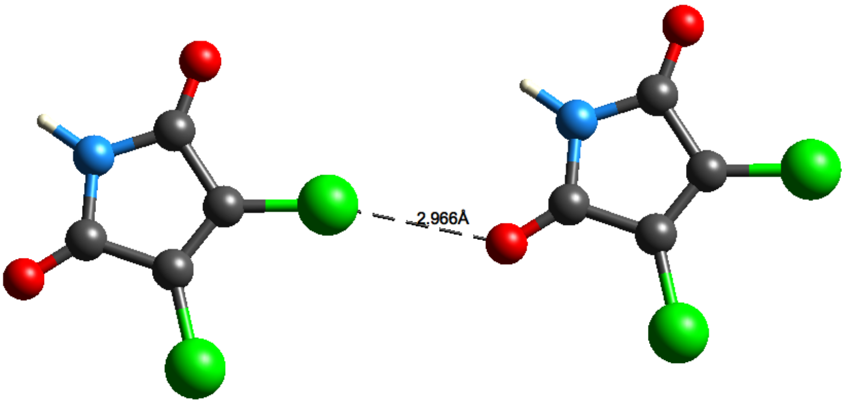

Figure S56: The molecular structure of IRUFEH01^s.

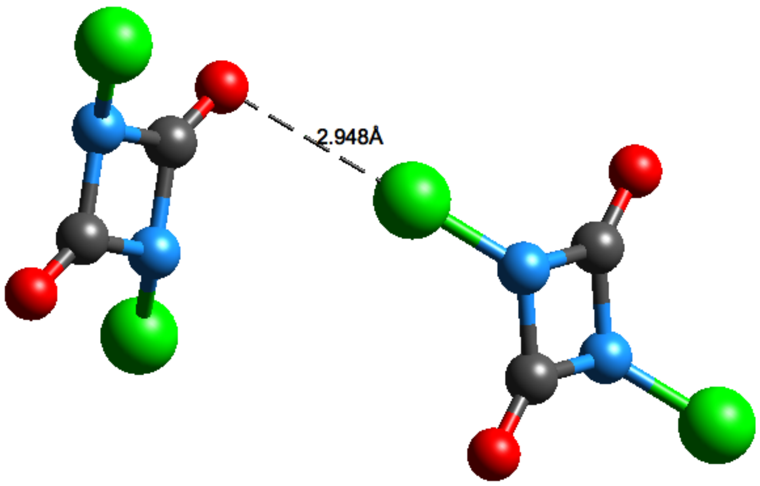

Figure S57: The molecular structure of JOJTIL^s.

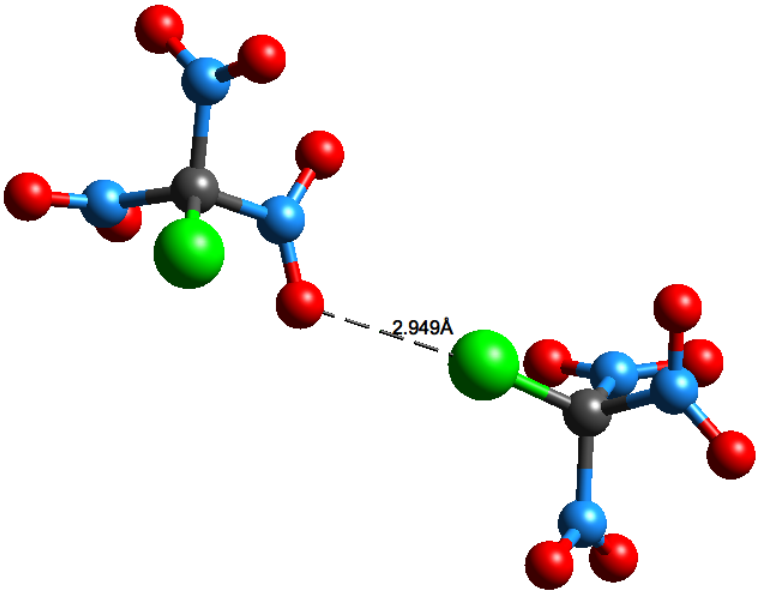

Figure S58: The molecular structure of RUBSUD^{s}.

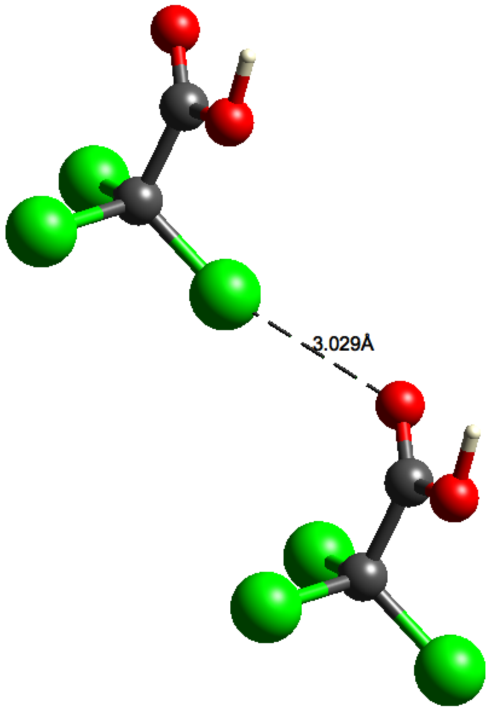

Figure S59: The molecular structure of TCACAD01^{s}.

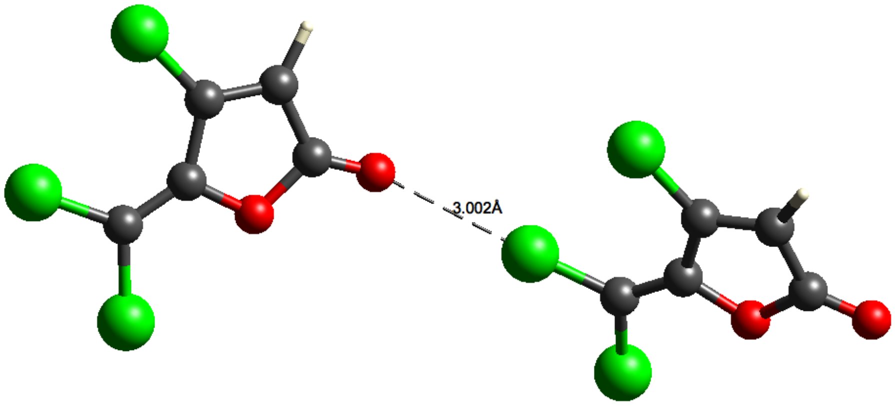

Figure S60: The molecular structure of GEXWUB^s.

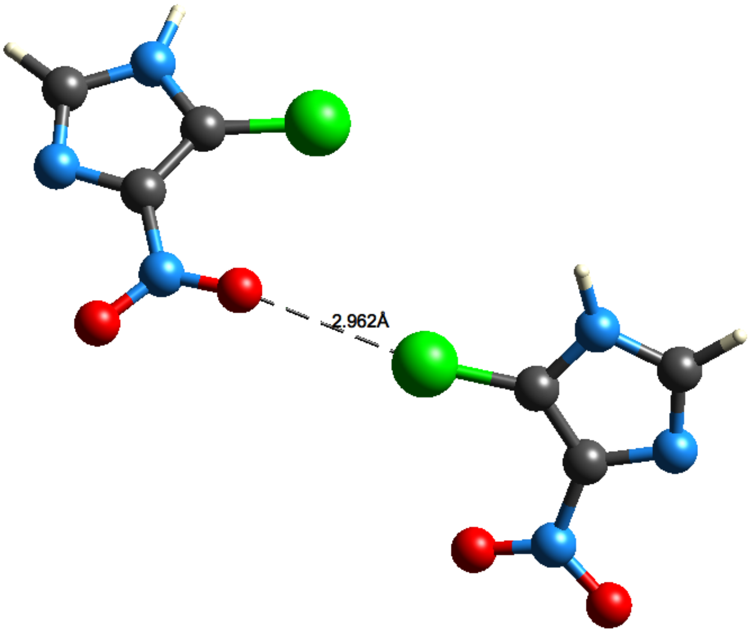

Figure S61: The molecular structure of PEPFUL^s.

S8.2.3 Br · · · N interactions

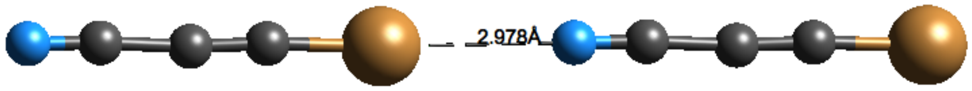

Figure S62: The molecular structure of BCACENN^s.

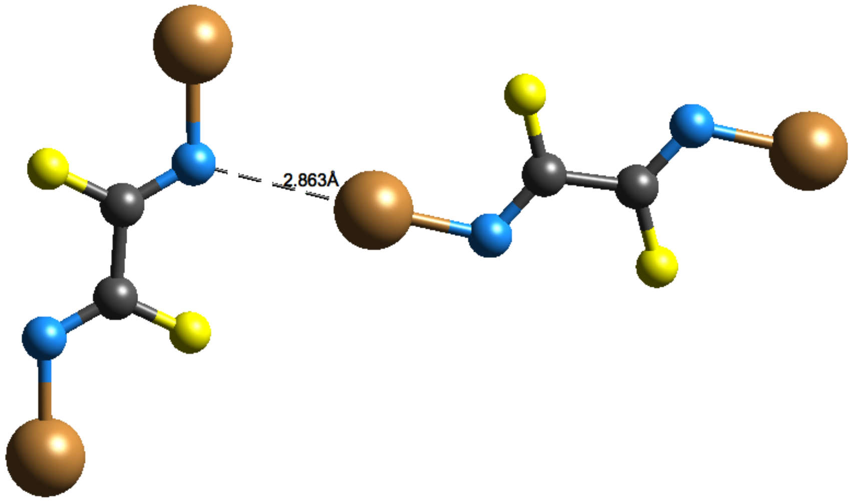

Figure S63: The molecular structure of BONFIT^s.

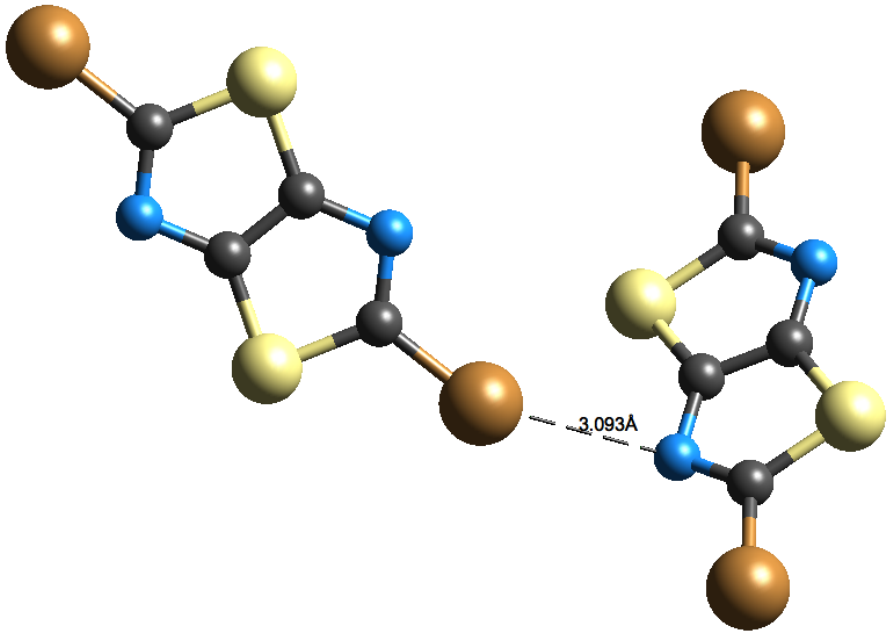

Figure S64: The molecular structure of QONHUX^s.

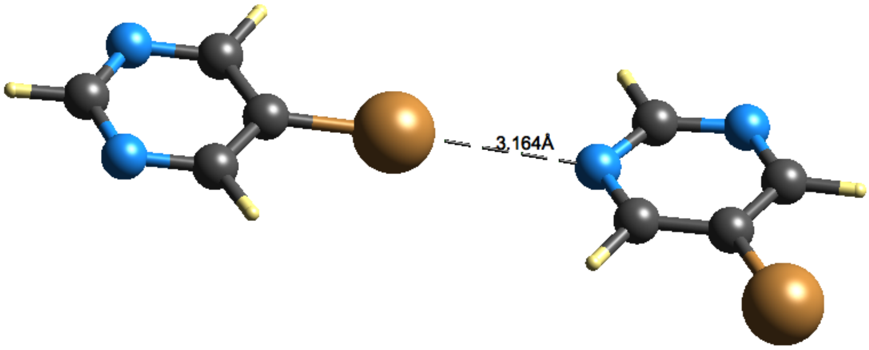

Figure S65: The molecular structure of RIRFOON^s.

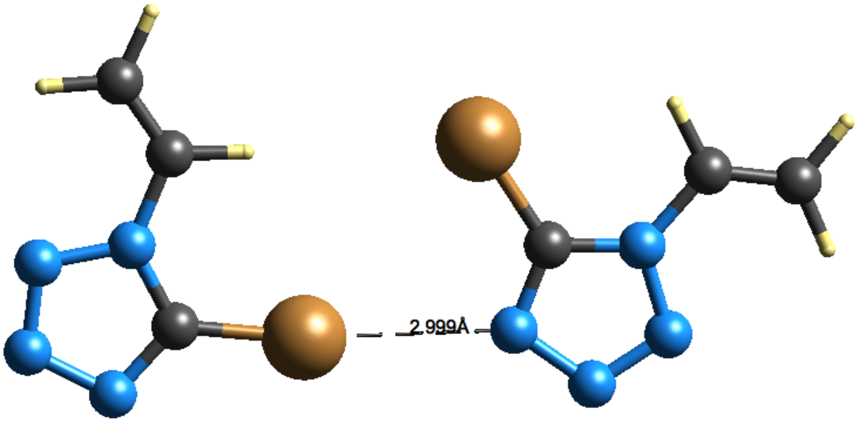

Figure S66: The molecular structure of KUYCUD^s.

S8.2.4 Br···O interactions

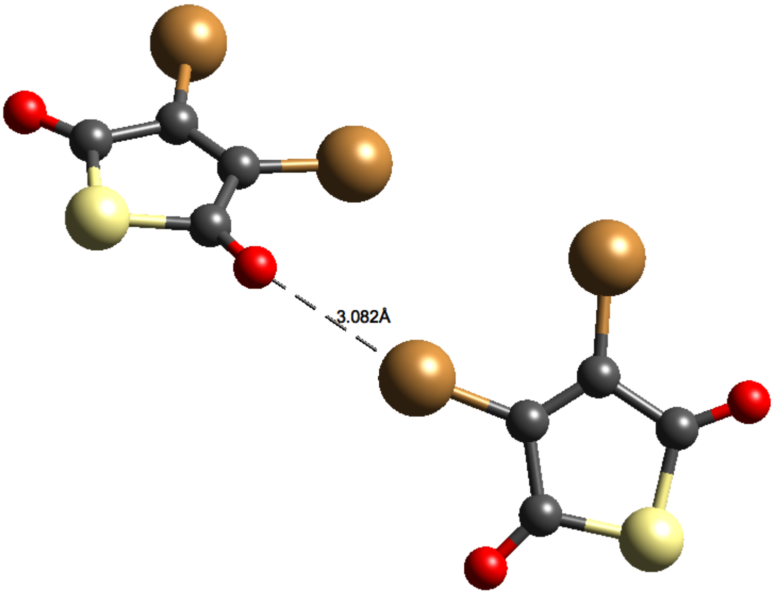

Figure S67: The molecular structure of BMLTAAN^{s}.

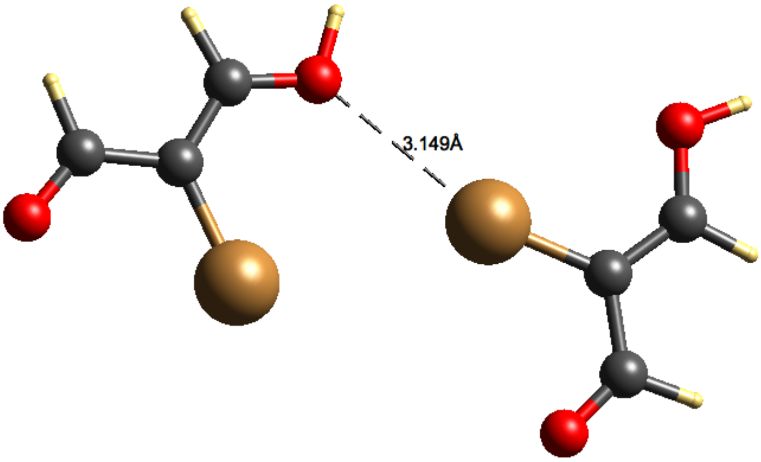

Figure S68: The molecular structure of CIRSONN^{s}.

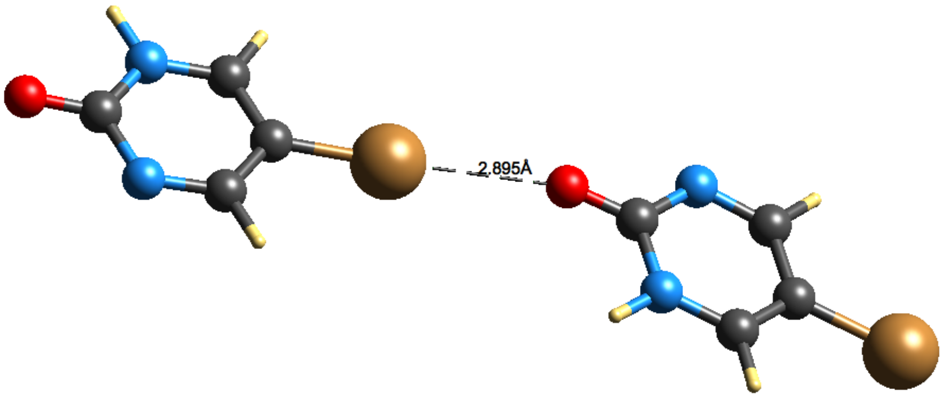

Figure S69: The molecular structure of JEVVOW^s.

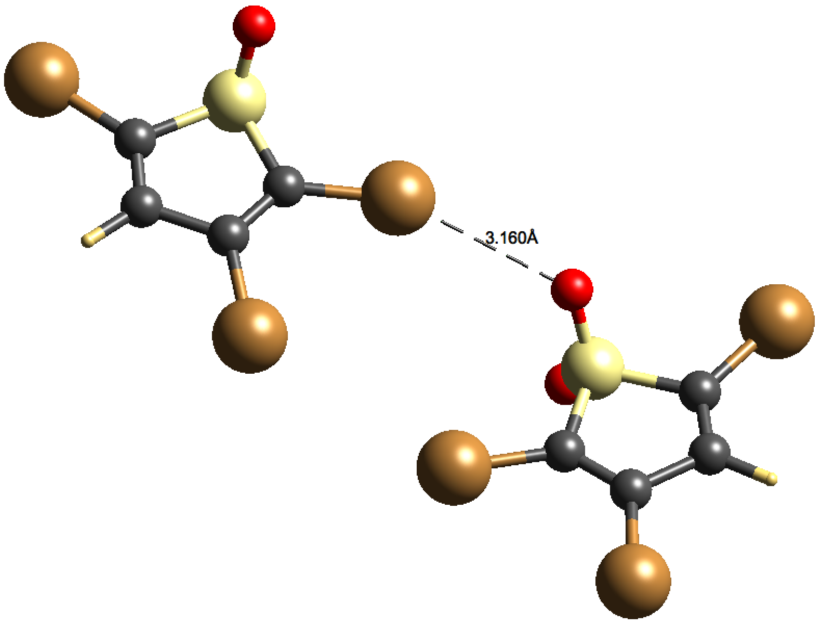

Figure S70: The molecular structure of VAQXUG^s.

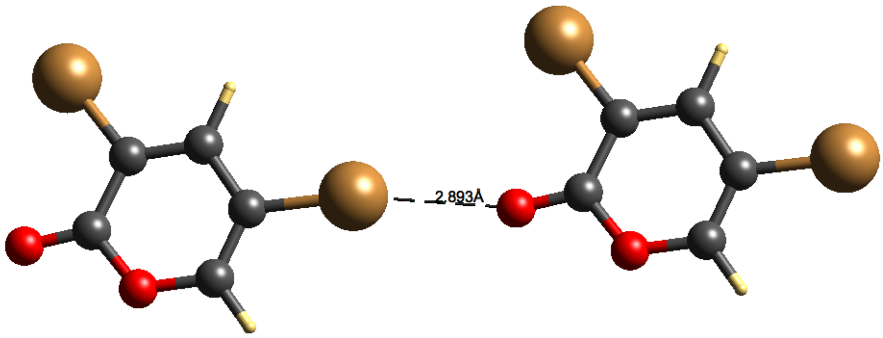

Figure S71: The molecular structure of VEWTAU^s.

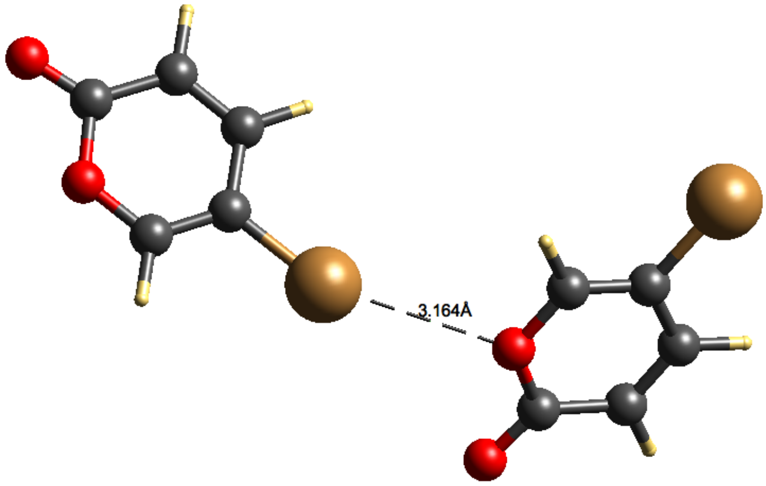

Figure S72: The molecular structure of VEWTEY^s.

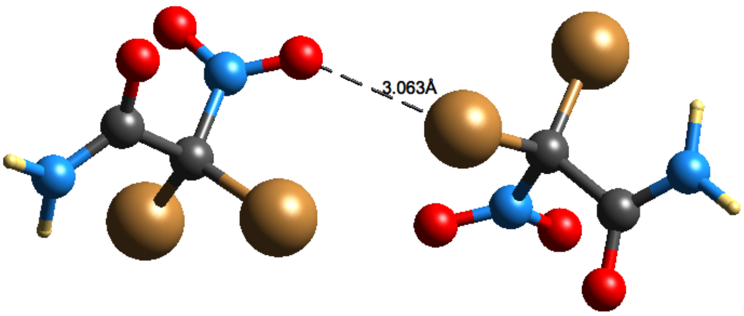

Figure S73: The molecular structure of VITVEZ^s.

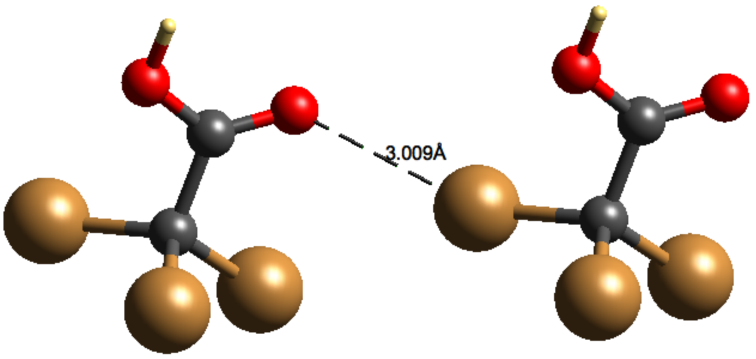

Figure S74: The molecular structure of WADFIR^s.

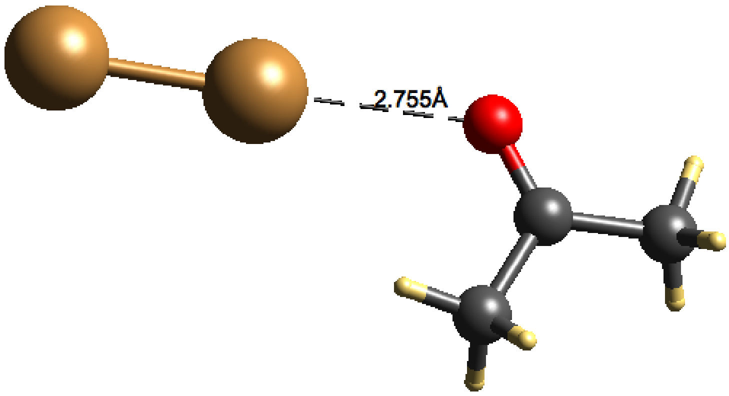

Figure S75: The molecular structure of ACETBR02^s.

S8.3 Chalcogen bonds

S8.3.1 S···N interactions

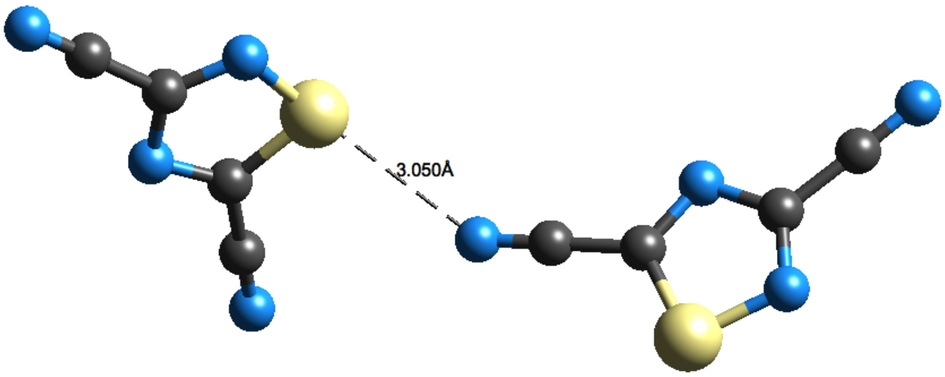

Figure S76: The molecular structure of CEBYUD^s.

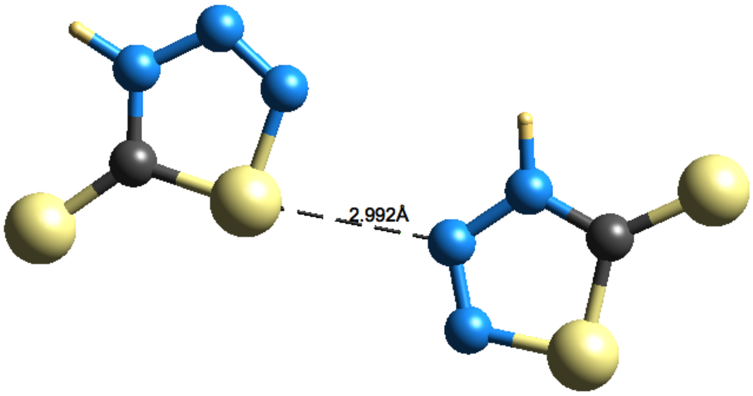

Figure S77: The molecular structure of QOBFUI^s.

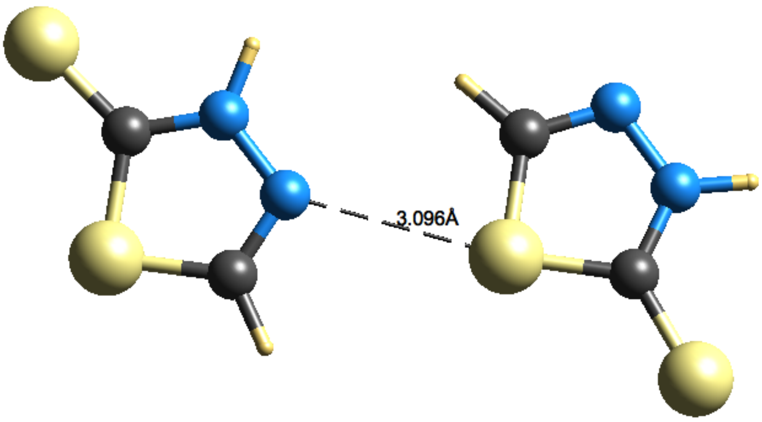

Figure S78: The molecular structure of SAZCEC^s.

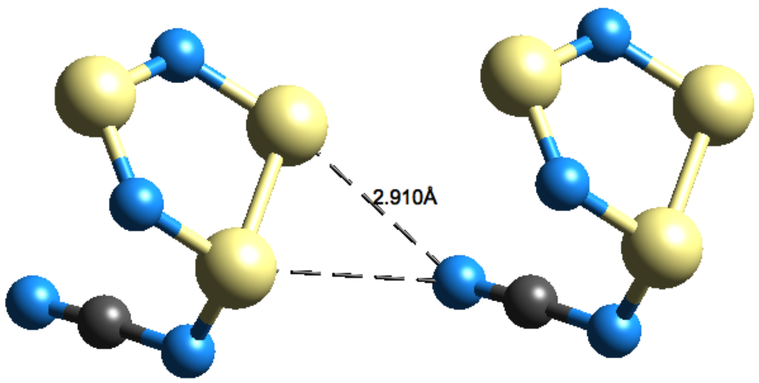

Figure S79: The molecular structure of GEDHAY^m.

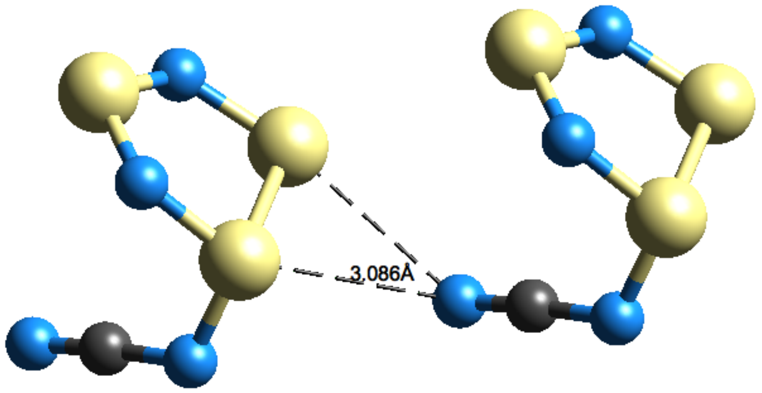

Figure S80: The molecular structure of GEDHAY^m.

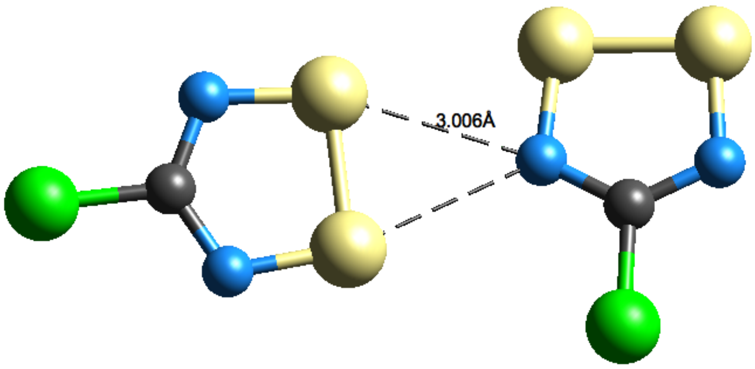

Figure S81: The molecular structure of IFULUQ04^m.

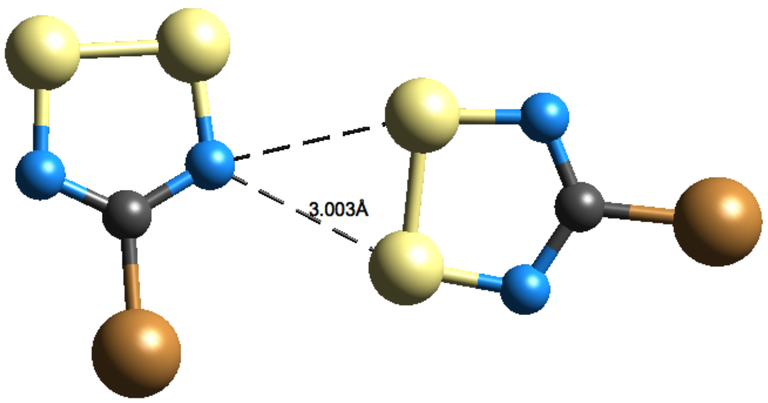

Figure S82: The molecular structure of WASHEE^m.

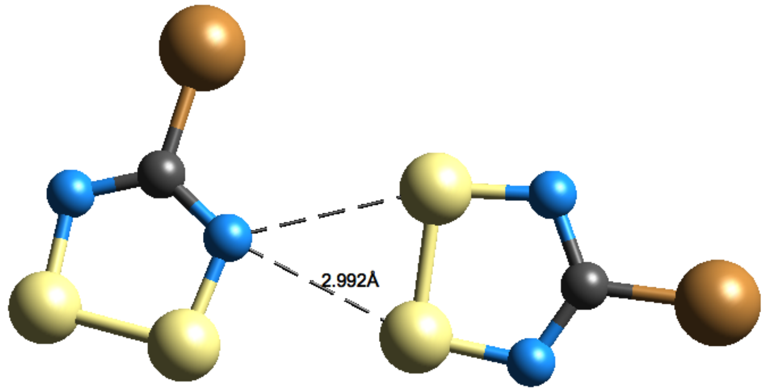

Figure S83: The molecular structure of WASHEE^m.

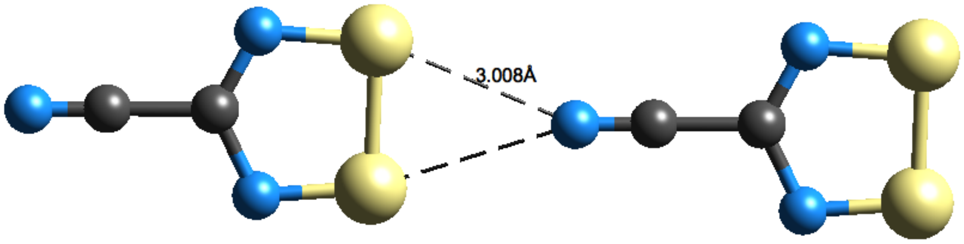

Figure S84: The molecular structure of WUXPAG^m.

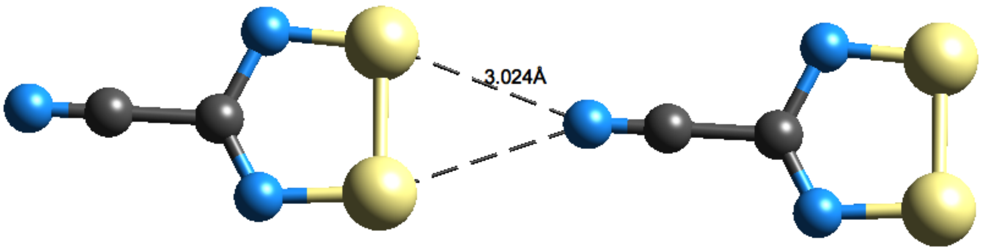

Figure S85: The molecular structure of WUXPAG^m.

S8.3.2 S$\cdots$O interactions

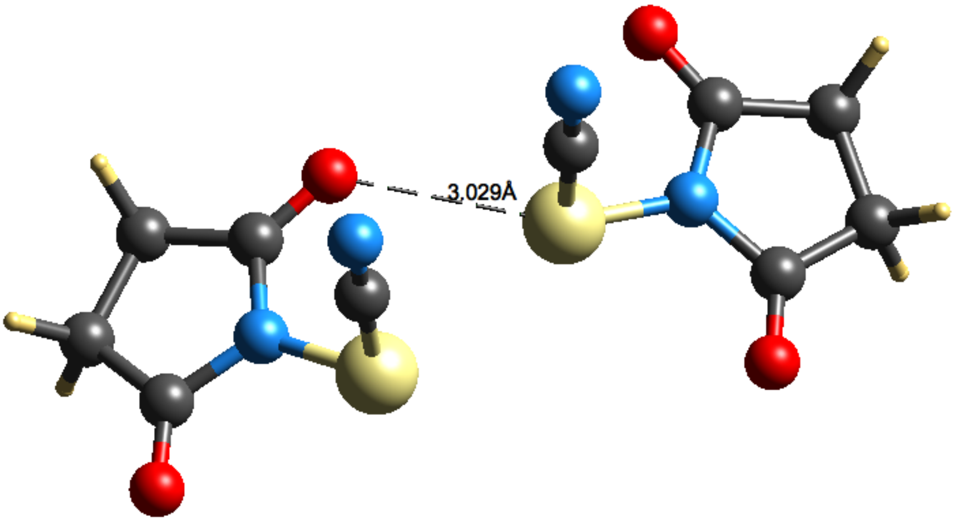

Figure S86: The molecular structure of PAFVEY^s.

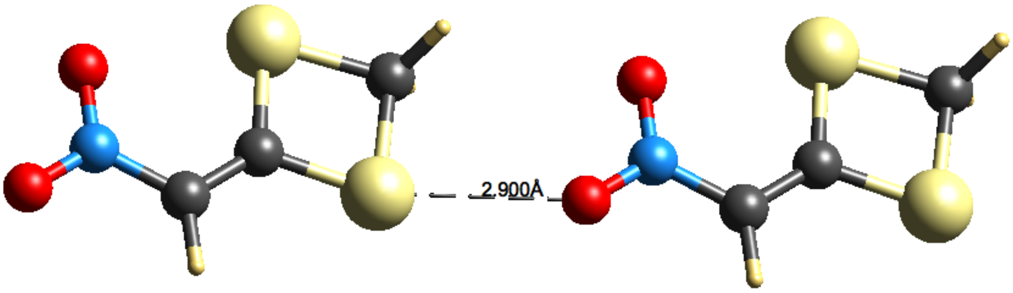

Figure S87: The molecular structure of WOCQEK^s.

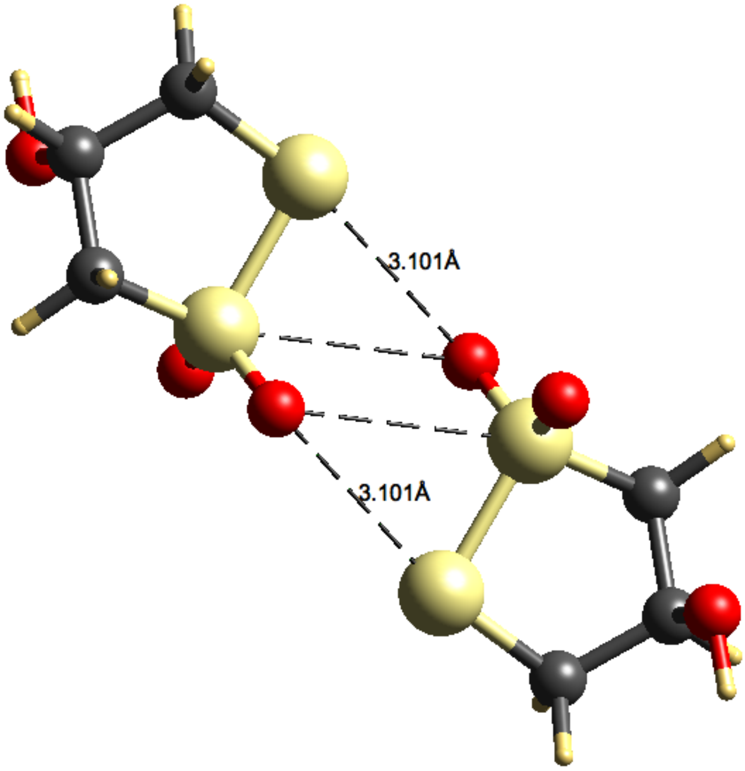

Figure S88: The molecular structure of ADOFEF^{m*}.

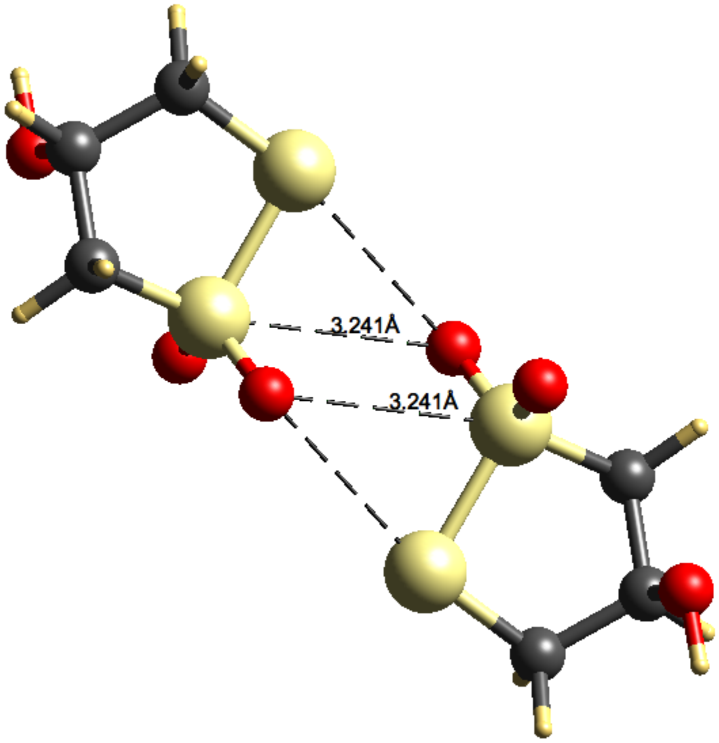

Figure S89: The molecular structure of ADOFEF^{m*}.

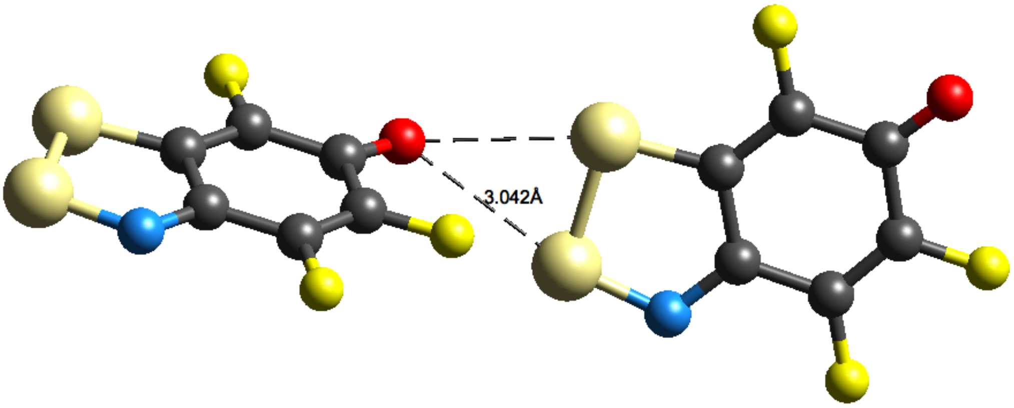

Figure S90: The molecular structure of MAVRAD^m.

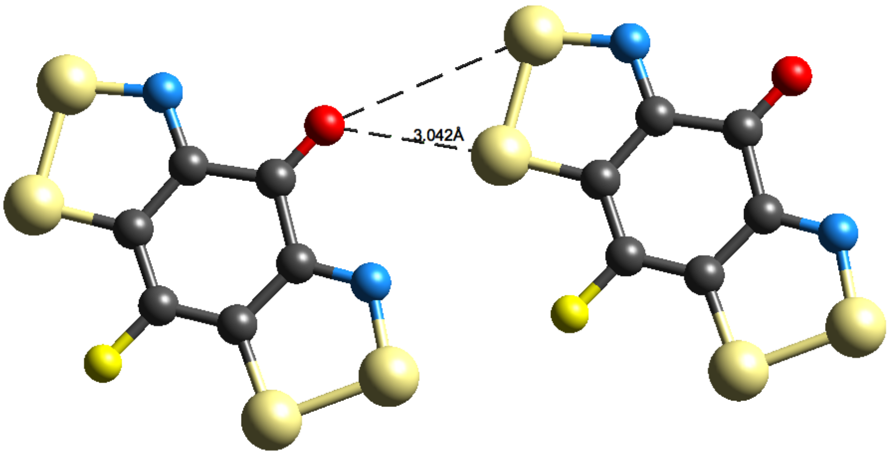

Figure S91: The molecular structure of MEHNIY^m.

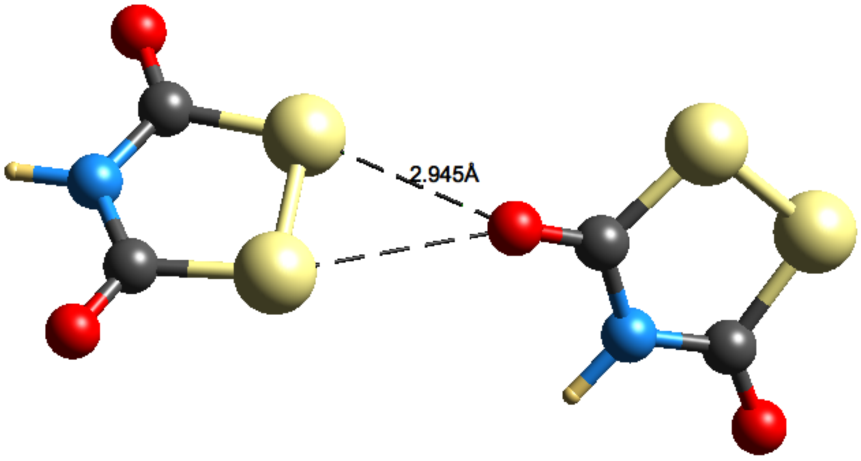

Figure S92: The molecular structure of NAHMUE^m.

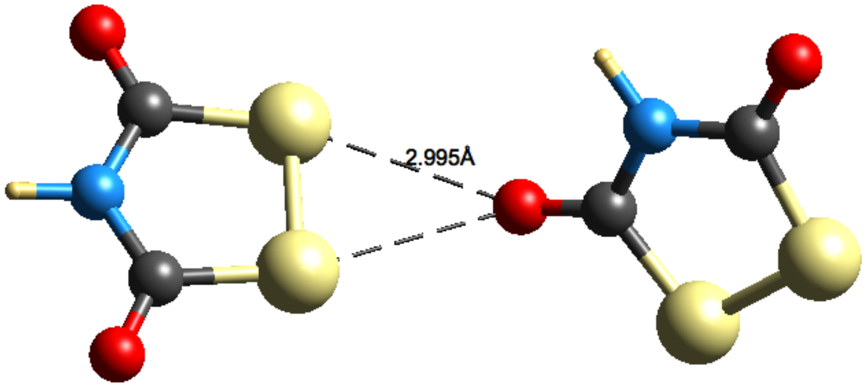

Figure S93: The molecular structure of NAHMUE^m.

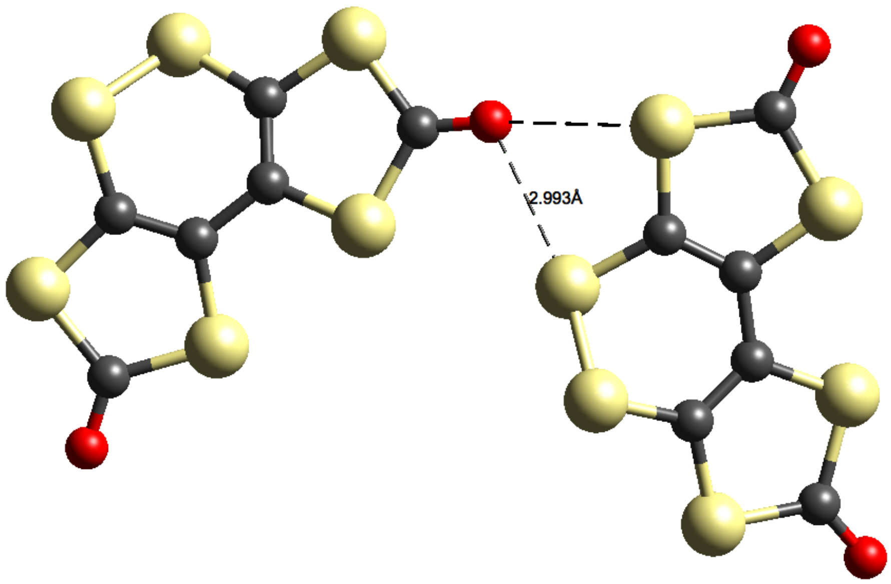

Figure S94: The molecular structure of PUDMUW^m.

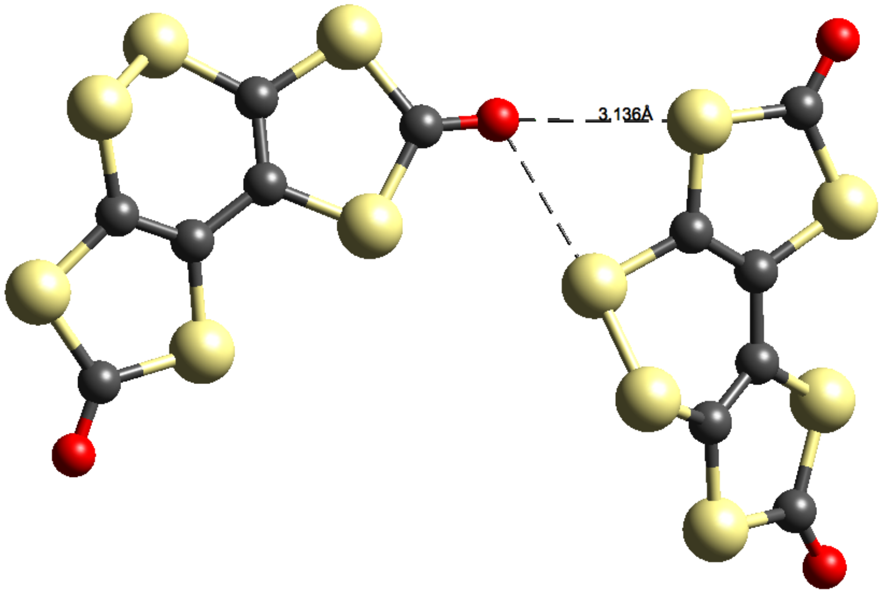

Figure S95: The molecular structure of PUDMUW^m.

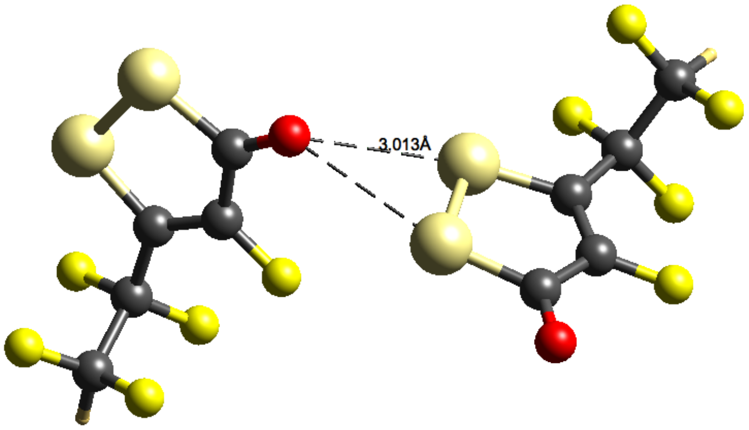

Figure S96: The molecular structure of QELQEE^m.

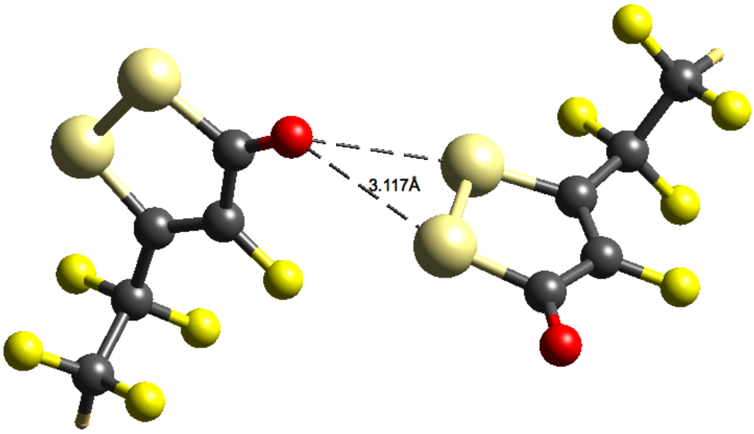

Figure S97: The molecular structure of QELQEE^m.

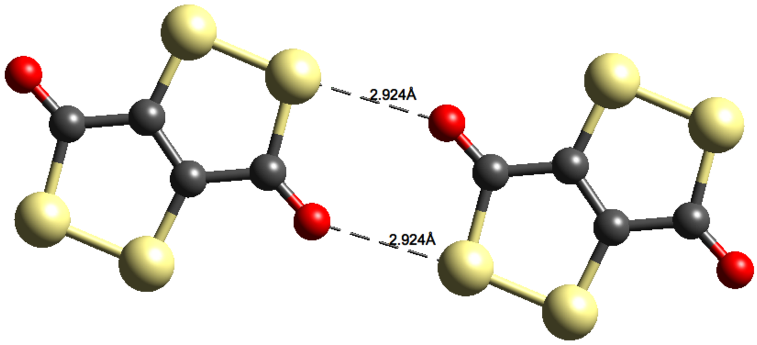

Figure S98: The molecular structure of ZAVHEJ^{m*}.

S8.3.3 Se···N interactions

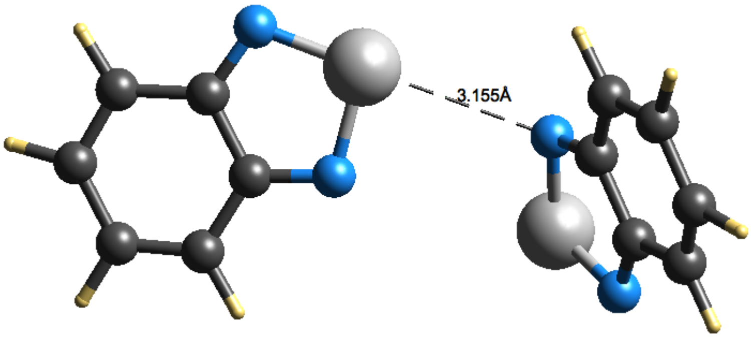

Figure S99: The molecular structure of BESEAZ01^{s}.

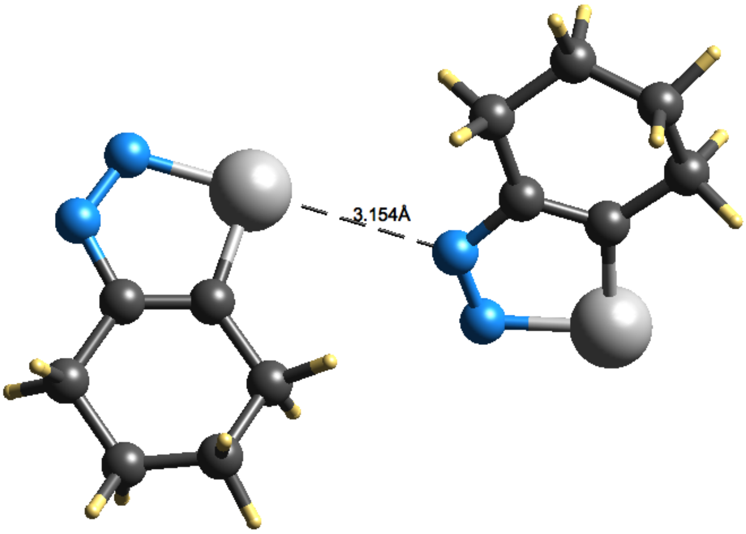

Figure S100: The molecular structure of FENFION^{s}.

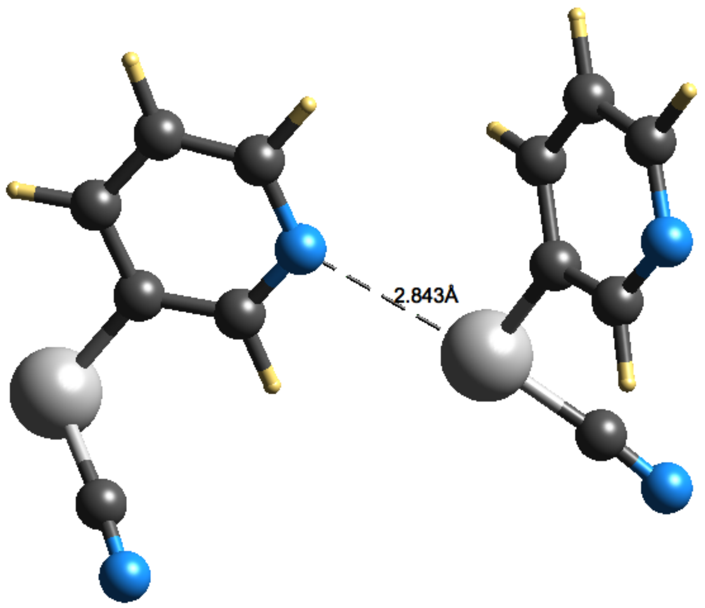

Figure S101: The molecular structure of WERYAT^{s}.

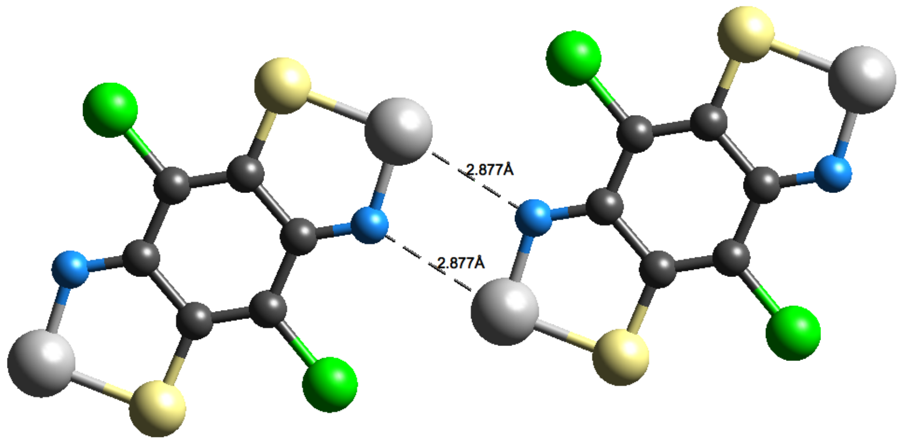

Figure S102: The molecular structure of NECZUQ^{m*}.

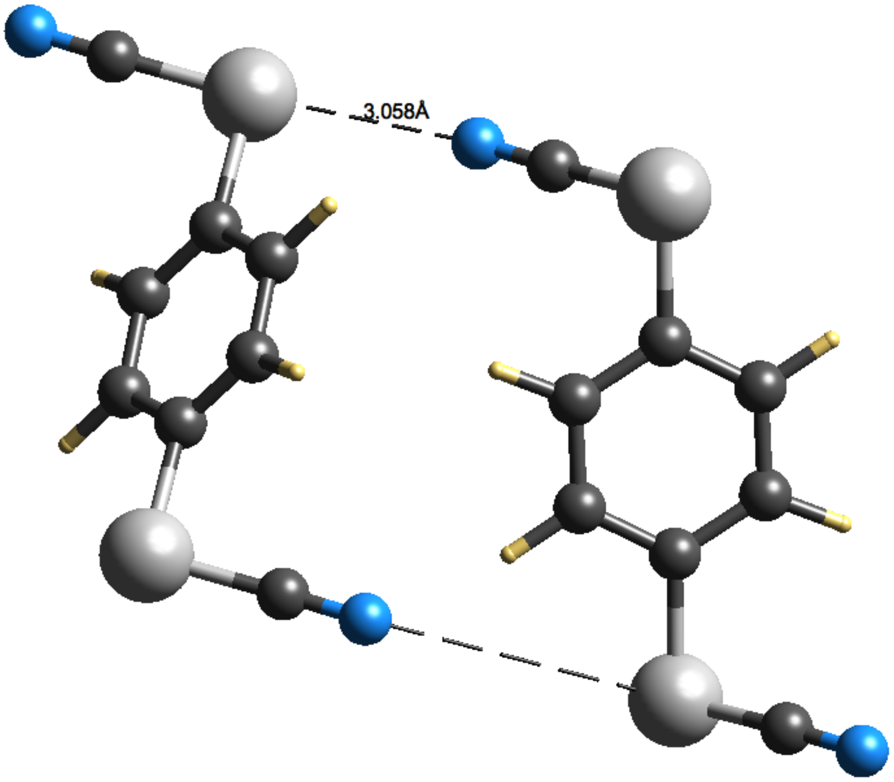

Figure S103: The molecular structure of SECNBZ^{m}.

S8.3.4 Se···O interactions

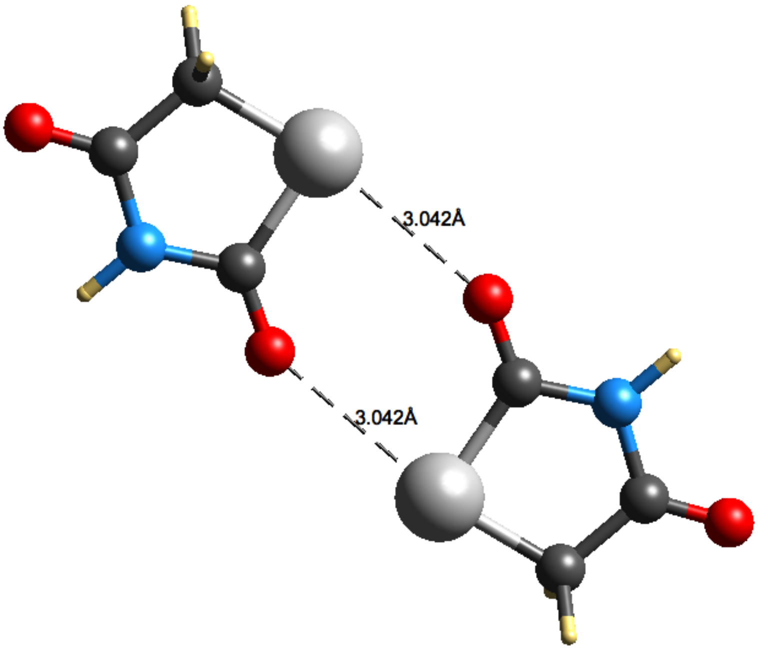

Figure S104: The molecular structure of BOJCOS^{m*}.

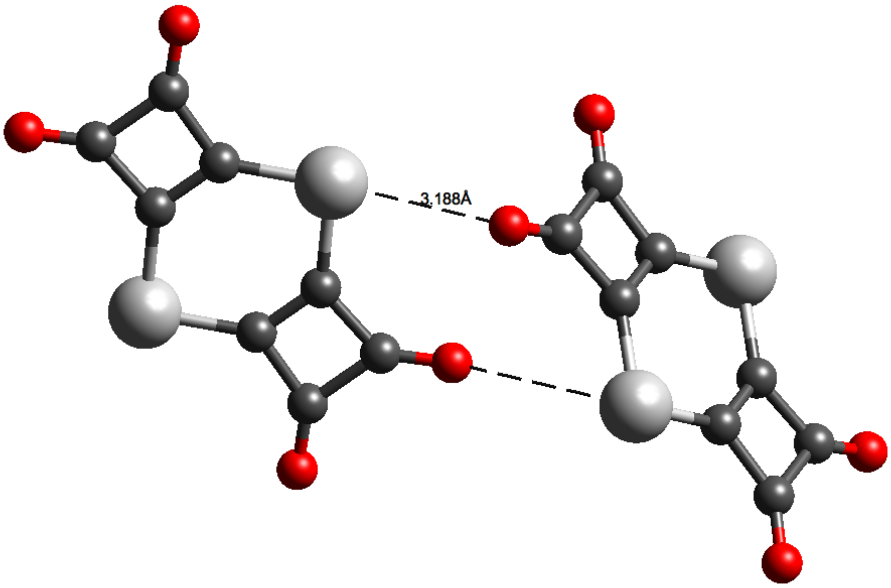

Figure S105: The molecular structure of LEDGAD^{m}.

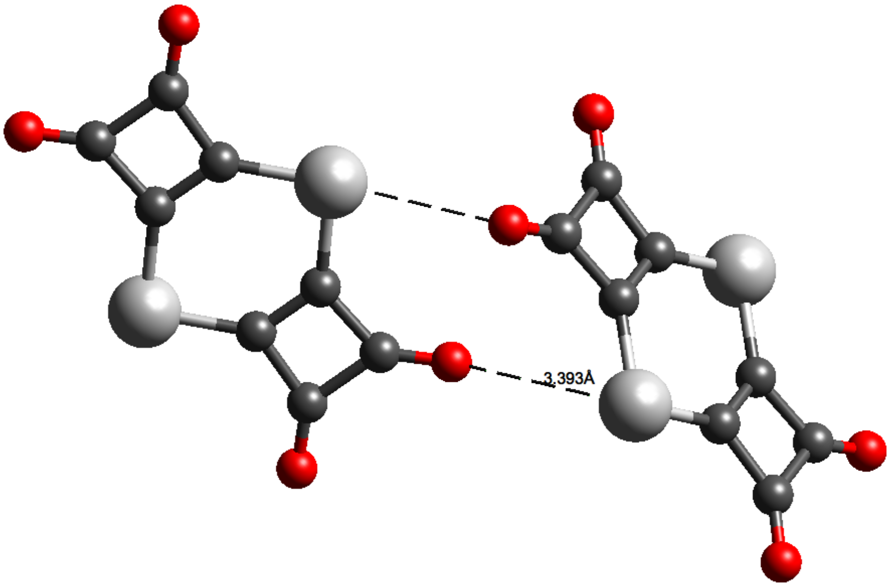

Figure S106: The molecular structure of LEDGAD^m.

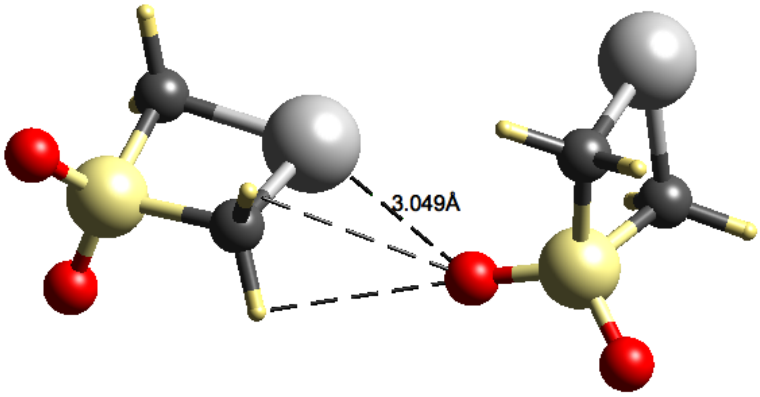

Figure S107: The molecular structure of LEVJOM^m.

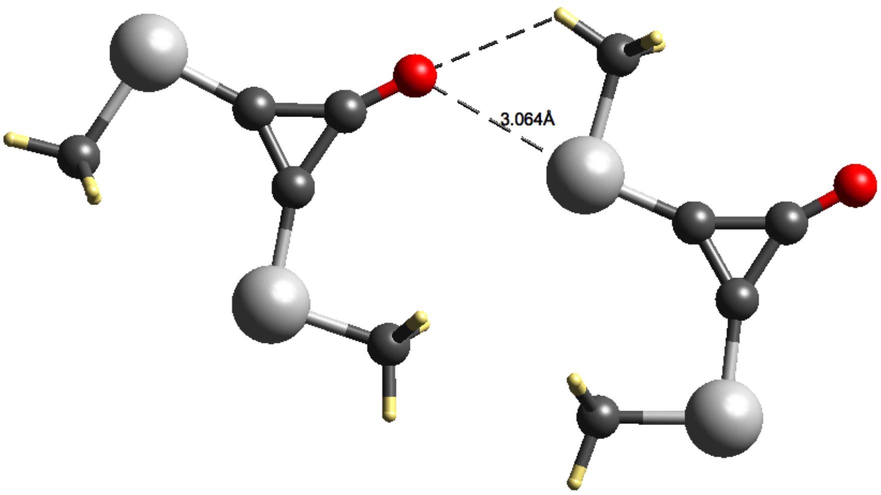

Figure S108: The molecular structure of MUSCIM^m.
